# A novel transcription factor *OsMYB73* affects grain size and chalkiness by regulating endosperm storage substances' accumulation‐mediated auxin biosynthesis signalling pathway in rice

**DOI:** 10.1111/pbi.14558

**Published:** 2024-12-26

**Authors:** Song Liu, Jiamin Wu, Amos Musyoki Mawia, Xiangjin Wei, Ruijie Cao, Guiai Jiao, Yawen Wu, Jian Zhang, Lihong Xie, Zhonghua Sheng, Shikai Hu, Sanfeng Li, Yusong Lv, Feifei Lu, Yujuan Chen, Sajid Fiaz, Javaria Tabassum, Zhimin Du, Fangyuan Gao, Guangjun Ren, Gaoneng Shao, Peisong Hu, Shaoqing Tang

**Affiliations:** ^1^ State Key Laboratory of Rice Biology (State Key Laboratory of Rice Biology and Breeding), China‐IRRI Joint Research Center on Rice Quality and Nutrition, Key Laboratory of Rice Biology and Genetics Breeding of Ministry of Agriculture, China National Center for Rice Improvement, China National Rice Research Institute Chinese Academy of Agricultural Sciences Hangzhou China; ^2^ Environment‐friendly Crop Germplasm Innovation and Genetic Improvement Key Laboratory of Sichuan Province, Key Laboratory of Tianfu Seed Industry Innovation (Co‐construction by Ministry and Province), Ministry of Agriculture and Rural Affairs, Crop Research Institute (Sichuan Provincial Germplasm Center) Sichuan Academy of Agricultural Sciences Chengdu China; ^3^ Institute of Molecular Biology and Biotechnology The University of Lahore Lahore Pakistan

**Keywords:** *OsMYB73*, endosperm starch and lipid biosynthesis, auxin biosynthesis, grain filling and endosperm development, grain size and chalkiness, yield and quality

## Abstract

Enhanced grain yield and quality traits are everlasting breeding goals. It is therefore of great significance to uncover more genetic resources associated with these two important agronomic traits. Plant MYB family transcription factors play important regulatory roles in diverse biological processes. However, studies on genetic functions of MYB in rice yield and quality are rarely to be reported. Here, we investigated a nucleus‐localized transcription factor *OsMYB73* which is preferentially expressed in the early developing pericarp and endosperm. We generated targeted mutagenesis of *OsMYB73* in rice, and the mutants had longer grains with obvious white‐belly chalky endosperm appearance phenotype. The mutants displayed various changes in starch physicochemical characteristics and lipid components. Transcriptome sequencing analysis showed that *OsMYB73* was chiefly involved in cell wall development and starch metabolism. *OsMYB73* mutation affects the expression of genes related to grain size, starch and lipid biosynthesis and auxin biosynthesis. Moreover, inactivation of *OsMYB73* triggers broad changes in secondary metabolites. We speculate that rice *OsMYB73* and *OsNF‐YB1* play synergistic pivotal role in simultaneously as transcription activators to regulate grain filling and storage compounds accumulation to affect endosperm development and grain chalkiness through binding *OsISA2*, *OsLTPL36* and *OsYUC11*. The study provides important germplasm resources and theoretical basis for genetic improvement of rice yield and quality. In addition, we enriches the potential biological functions of rice MYB family transcription factors.

## Introduction

Rice (*Oryza sativa* L.) is one of the most important staple food crops in the world that feeds more than half of the world's population (Jiang *et al*., 2012). Rice grain yield and quality are important agronomic traits and ultimate breeding goals. Despite the remarkable increase in yield during the green revolution era, agricultural scientists are still facing severe challenge in ensuring production of enough food to feed the ever‐increasing human population (Chen *et al*., 2022). Therefore, breeding and adoption of new cultivars with improved grain yield and quality is a top priority.

Grain size is one of the major determinants of rice yield and appearance quality (Xing and Zhang, 2010). Up to date, many genes related to grain size and weight have been identified and found to be involved in various signalling pathways, including G protein signalling pathway, mitogen activated protein kinase (MAPK) pathway, ubiquitin‐mediated proteasome degradation pathway, phytohormone biosynthesis pathway and transcriptional regulation (Zuo and Li, 2014; Li *et al*., 2019). The regulatory network contributing to rice grain size can provide an important germplasm resources and theoretical supports for breeding new varieties with high yield and quality.

Starch is a major component of rice endosperm which directly establishes grain yield and quality. Starch biosynthesis requires the participation of many key enzymes. Great progress has been made in the functional analysis of key starch synthesis‐related genes (SSRGs) in rice endosperm and their correlations with the physicochemical properties of starch with different components and structures. Rice starch typically consists of approximately 20% amylose and 80% amylopectin (Crofts *et al*., 2015; Tetlow, 2011). Amylose synthesis requires only two enzymes: ADP‐glucose pyrophosphorylase (AGPase) and granule‐bound starch synthase (GBSS). In contrast, the synthesis of amylopectin involves the combined activity of AGPase, soluble starch synthases (SSs), starch branching enzymes (SBEs), debranching enzymes (DBEs) and phosphorylases (PHOs), the latter forming a compound with disproportionating enzymes (DPEs) (Qu *et al*., 2018; Tian *et al*., 2009). Inactivation of rice starch branching enzyme OsBEIIb triggers broad and unexpected changes in starch metabolism and carbohydrate repartitioning by transcriptional reprogramming (Baysal *et al*., 2020; Chen *et al*., 2022). *Rice Like Early Starvation1 (OsLESV/flo9)* interacts with *Floury Endosperm 6 (flo6)* to modulate starch biosynthesis and endosperm development (Yan *et al*., 2024). *FLOURY ENDOSPERM 24*, a heat shock protein 101 (HSP101), is required for starch biosynthesis and endosperm development in rice (Wu *et al*., 2024b). Natural variation in *WHITE‐CORE RATE 1* (*WCR1*) regulates redox homeostasis in rice endosperm to affect grain quality (Wu *et al*., 2022). *OsTIR1* markedly influences rice endosperm starch biosynthesis with grain filling (Wu *et al*., 2024b). The *OsIAA29‐OsARF17* module regulates starch biosynthesis in rice seed at high temperature through auxin signalling (Chen *et al*., 2024). To study the regulatory mechanism of starch quality, researchers used gene co‐expression analysis to identify genes that may be involved in starch synthesis. For instance, *RSR1* negatively regulates the expression of Type I starch synthesis genes and its deficiency results in the improved expression of starch synthesis genes in rice grains (Fu and Xue, 2010). We focused on one of them rice *OsMYB73* transcription factor.

Several transcription factors (NF‐YB, NAC, bZIP, MADS and MYB) have been reported to directly regulate the expression of starch biosynthesis genes in rice endosperm. *OsMADS1* regulates networks of starch and storage protein metabolism (Liu *et al*., 2023). Both *OsbZIP58* and *RPBF* transcriptional factors, behave synergetically to regulate the mechanism of genes involved in starch and protein synthesis during endosperm development (Kawakatsu *et al*., 2009). *OsbZIP60*‐mediated unfolded protein regulates grain chalkiness (Yang *et al*., 2022) and endosperm storage protein and starch biosynthesis at high temperature (Cao *et al*., 2022). OsbZIP10 modulates rice grain quality by regulating *OsGIF1* and *OsAGPS1* (Jiang *et al*., 2024). *OsNAC20* and *OsNAC26* bind directly to key genes of starch and protein synthesis in endosperm, and the *osnac20/26* double mutant remarkably reduced starch and storage protein content (Wang *et al*., 2020). *OsNAC127* and *OsNAC129* form heterodimers that participate in apoplastic transport and the heat stress response to regulate starch accumulation and grain filling (Jin *et al*., 2022). The OsNAC24‐OsNAP protein complex activates *OsGBSSI* and *OsSBEI* expression to fine‐tune starch biosynthesis in rice endosperm (Jin *et al*., 2023). *OsNF‐YB1*, a member of the nuclear factor Y family, is predominantly specific expressed in the aleurone layer of rice endosperm development (5–7 DAF, days after fertilization), it binds with *OsNF‐YC12* and *OsbHLH144* to form the NF‐Y heterotrimer compound, and mutation of each gene in the compound can alter starch synthesis in rice endosperm (Bello *et al*., 2019). Rice grain quality is also being regulated by ABA growth regulator via *NF‐YB1*‐*SLRL2*‐*bHLH144* module (Wang *et al*., 2024c). *OsMADS14* and *OsNF‐YB1* cooperate in the direct activation of *OsAGPL2* and *Waxy* during starch synthesis (Feng *et al*., 2022). ChIP‐qPCR assays further confirmed the direct binding of *OsNF‐YB1* to selected genes, including sugar transporters (SUT) and MYB transcription factors (Xu *et al*., 2016) and *OsYUC11* (Xu *et al*., 2021). Further identification of target genes of MYB in endosperm cells will help to elucidate the complexity and diversity of the mechanisms that regulate starch biosynthesis.

Lipids in rice endosperm plays an important role in determining cooking and storage qualities. Four genes (*OsPAL6*, *OsLIN6*, *OsMYR2* and *OsFAE6*) found to contribute to natural variation in oil composition, these genes have the potential for facilitating marker‐based breeding of rice varieties with enhanced oil and grain quality (Zhou *et al*., 2021). Crude fat content of rice, including palmitic acid (16 : 0), stearic acid (18 : 0), oleic acid (18 : 1), linoleic acid (18 : 2, 18 : 3) and several other components, are important factors that determine rice appearance and eating quality (Ying *et al*., 2012). It was reported that the down‐regulation of *OsLTPL36* resulted in reduced fat acid content in rice seed, caused chalky endosperm, and decreased seed setting rate and 1000‐grain weight in transgenic plants (Wang *et al*., 2015). In addition, multi‐gene engineering of *AtDGAT1/OsAGPL2/OsMTSSB1* increased oil content in rice grains from 2.3% to 11.7% (Liu *et al*., 2024b).

Plant hormones are closely related to reproductive development. For instance, *ZmNAC128* and *ZmNAC130* transcriptional factors have been found to coordinate the biosynthesis of storage reserves and indole‐3‐acetic acid (IAA) in maize endosperm (Song *et al*., 2024). In rice plant, *OsARF18*‐*OsARF2*‐*OsSUT1* mediated auxin signalling cascade regulating carbohydrate partitioning and reproductive organ development between the source and sink tissues (Zhao *et al*., 2022). *OsSK41*‐*OsIAA10*‐*OsARF* regulates grain yield traits in rice by auxin signalling pathways (Ma *et al*., 2023). Endosperm development regulated by Aux/IAA‐OsARF also affects grain size (Li *et al*., 2019). The grain filling defect of auxin‐insensitive *ostir1* mutant is regulated by Aux/IAA‐OsARF, the elevated OsIAA1 protein levels in the *ostir1* inhibit *OsARF25* binding to the promoter of *OsSWEET11* that controls the endosperm filling process, causing defects in endosperm development and ultimately reducing grain size (Wu *et al*., 2024a). IAA may control sugar transport and unloading by regulating dorsal vascular bundle development, consequently affecting endosperm development (Yu *et al*., 2024). *OsMYB14* regulates plant height through auxin metabolism (Kim *et al*., 2024). However, the regulatory mechanism on how auxin regulates rice endosperm development and grain quality is still unclear.

Plant MYB family transcription factors play important regulatory roles in diverse biological processes such as cell morphogenesis, regulation of growth and development, biotic and abiotic stress responses, and secondary metabolite biosynthesis. For instance, *N‐mediated‐heading‐date* (*Nhd1*) encodes a MYB TF and regulates endosperm starch synthesis. Overexpression of *Nhd1* reduces amylose content through downregulation of *GBSSI*. Its overexpression also enhances amylopectin content through upregulation of *SSII‐3/SSIIa* and *FLO5* during the grain filling stage (Zhang *et al*., 2023). The *AtMYB118* regulates the specific compartmentalization of some stored compounds in embryo and endosperm, which regulates compounds biosynthesis at spatial level during maturation (Barthole *et al*., 2014). The maize MYB TF *ZmMYB14* and *ZmMYB71* bind to promoters of multiple starch‐synthesis‐related genes (SSRGs) to activate and suppress their expression, respectively (Han *et al*., 2024; Xiao *et al*., 2017). *ZmMYBR29* regulates starch synthesis and carbohydrate metabolism to affect the maize grain filling (Wu *et al*., 2024c). The preferentially expressed in the developing endosperm MYB family transcription *TuODORANT1* inhibits seed storage protein synthesis in wheat (Luo *et al*., 2021). *TaMYB72* negative regulates grain length in wheat (Wu *et al*., 2024d). However, rice MYB transcription factors involve endosperm starch and lipid biosynthesis to affect yield and quality traits are still rarely reported. Therefore, it is necessary to explore and enrich more potential biological functions of MYB family in rice. In addition, little is known about how MYB TFs maintain the balance between quality and yield through simultaneous regulation of grain size and weight, starch and lipid synthesis.

In the present study, we successfully generated *OsMYB73* mutants by using CRISPR/Cas9 genome editing system. The *myb73* mutants showed longer grains and white‐belly chalky endosperm phenotype. In addition, we investigated the effects of *OsMYB73* mutation on morphology and physicochemical characteristics of starch granules in mutants lines. Furthermore, we also analysed the expression of genes related to grain size and weight, starch and lipid biosynthesis, and auxin biosynthesis. Moreover, we evaluated the wider huge impact on many pathways by transcriptomics and metabolomics. Taken together, this study demonstrates that *OsMYB73* not only affects rice grain size and weight, but also endosperm starch and lipid biosynthesis, and auxin biosynthesis, primary and secondary metabolites. We speculate that rice *OsMYB73* and *OsNF‐YB1* paly synergistic pivotal role in simultaneously as transcription activators to regulate grain filling and storage compounds accumulation to affect endosperm development and grain chalkiness through binding *OsISA2*, *OsLTPL36* and *OsYUC11*. Our study provides more germplasm resources and theoretical basis that may be used to breed new cultivars with enhanced grain yield and quality.

## Results

### A phylogenetic analysis and protein domain prediction of rice OsMYB73

Rice endosperm starch biosynthesis is a critical factor in grain quality and nutrition, scientists found co‐expressed transcription factor coding genes correlated with type I starch synthesis genes expressed in seeds, including five MYB transcription factor family genes (Fu and Xue, 2010), we focused on one of them *OsMYB73* transcription factor. First, rice OsMYB73 protein phylogenetic tree was constructed and the results showed a close homology relationship with OsMYB118, OsMYB119, ZmMYB98 (*Zea mays*) and SbMYB80 (*Sorghum bicolor*) (Figure S1a). OsMYB73 does not have a close homology relationship with other several seed development MYB domain genes including *Osmyb1*, *Osmyb2*, *Osmyb3*, *Osmyb4*, *Osmyb5* and *ZmMYB14*. The structure of OsMYB73 protein was also predicted and the results revealed that it has two typical SANT domains between 115–164 and 167–215 amino acids, which may play a role in transcriptional activation or repression regulation of the downstream targeted genes (Figure S1b).

### Rice *OsMYB73* is preferentially expressed in early developing pericarp and endosperm which encoding protein localized in the cell nucleus

**Figure 1:**
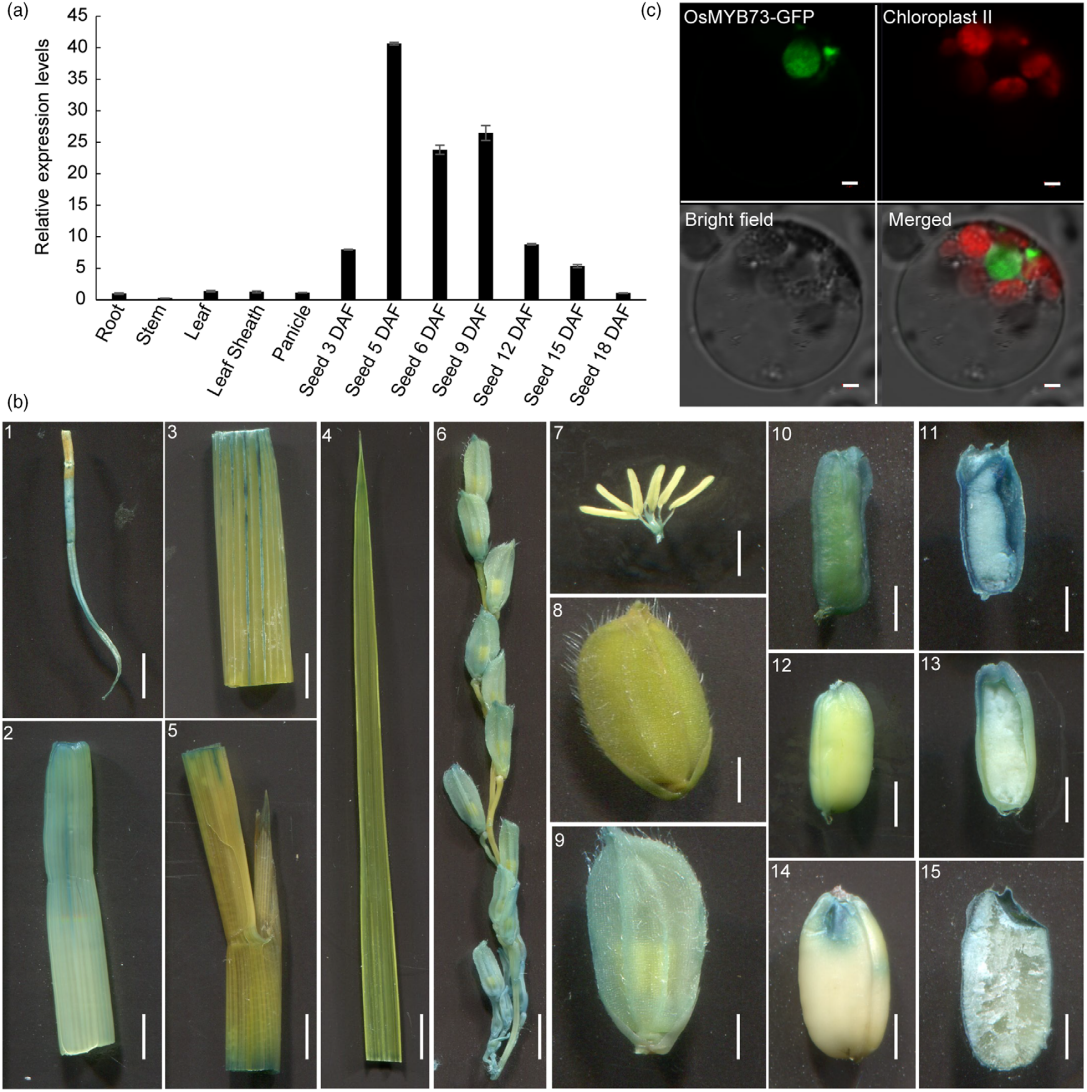
Rice *OsMYB73* gene expression at various tissues, histochemical GUS staining and subcellular localization of OsMYB73 protein. (a) Rice *OsMYB73* gene expression at various tissues. R, root; S, steam; L, leaf; LS, leaf sheath; P, panicle; 3D, 5D, 6D, 9D, 12D, 15D, 18D means 3, 5, 6, 9, 12, 15, 18 days after fertilization. Expression level at root in the wild‐type was set as reference value of 1. Data are mean ± SD (*n* = 3). (b) Histochemical GUS staining. Scale bars are 1.0 cm in B. (1): root; (2): steam; (3, 4): leaf; (5): leaf sheath; (6): branch; (7): floret; (8, 9): spikelet; (10, 11): Developing seed and transection of 5 days after fertilization; (12, 13): Developing seed and transection of 10 days after fertilization; (14, 15): Mature brown rice and transection. (c) Subcellular localization in rice protoplasts, bars are 2 μm.

Spatio‐temporal profile of rice *OsMYB73* shows that the transcripts could be mainly detected during rice grain filling development process (Figure S2). Quantitative real‐time PCR (qRT‐PCR) was used to detect the expression levels of *OsMYB73* in various tissues in different days after fertilization (DAF). The results showed that *OsMYB73* was highly expressed in developing endosperm, with the highest expression detected during early endosperm filling stage (5 DAF), while expression in other tissues was very low (Figure 1a). Histochemical GUS staining was performed on various tissues of *pOsMYB73:GUS*‐positive plants and the results showed that *OsMYB73* is highly expressed in seeds of 5 DAF and then gradually decreased along with the grains filling until maturity. However, the expression levels were extremely low in roots, stems, leaves, leaf sheaths and young panicle (Figure 1b), which was consistent with the qRT‐PCR results. In conclusion, rice *OsMYB73* is highly expressed in developing pericarp and endosperm. The GFP results revealed that encoding protein OsMYB73 is localized in the cell nucleus (Figure 1c).

### Knockout of rice *OsMYB73* displayed longer grains and white‐belly chalky endosperm appearance phenotype

**Figure 2:**
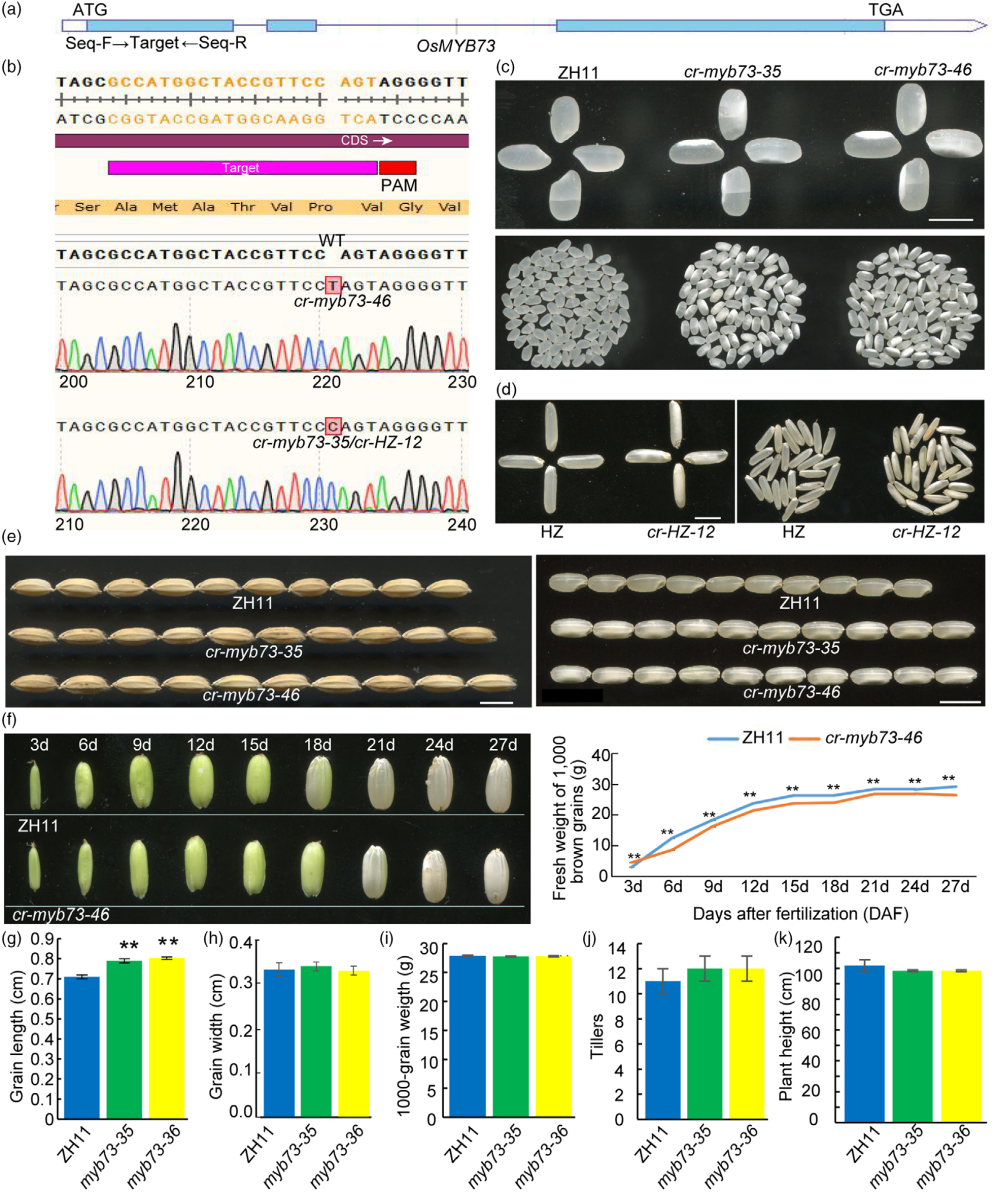
CRISPR/Cas9 mediated target mutagenesis of *OsMYB73* and genotype and phenotype identification. (a) The gene structure of *OsMYB73* in rice; (b) target site selection and genotype identification; (c) milled rice phenotype identification in T_1_ generation (CK: ZH11), scale bar is 1.5 cm; (d) dehusked rice phenotype identification in T_1_ generation (CK: HZ), scale bar is 1.0 cm; (e) grain length between wild‐type and mutants, scale bar are 1.0 and 1.5 cm; (f) grain filling rate between wild‐type and mutants; (g) grain length between wild‐type and mutant; (h) Grain width between wild‐type and mutants; (i) 1000‐grain weight between wild‐type and mutants; (j) tillers between wild‐type and mutants; (k) plant height between wild‐type and mutants.

To study the regulatory function of rice *OsMYB73*, we first analysed its structure. Its whole genome length is 2381 bp including 3 exons and 2 introns, with a CDS length of 1347 bp and encoding 448 amino acids (Figure 2a). On the first exon of *OsMYB73* gene, a specific 20 bp sequence was selected as knockout target site, and AGG was used as PAM sequence. The vectors were transformed into *japonica* rice variety Zhonghua 11 (ZH11) and *indica* rice variety Huazhan (HZ). We obtained different mutants in T_1_ generations. We obtained two alleles of ZH11 background, one containing a C insertion (*cr‐myb73‐35*) and another one containing T insertion (*cr‐myb73‐46*). In addition, we generated one allele (*cr‐HZ‐12*) containing C insertion under the background of HZ. Although the mutation position (92 amino acid) was not in the SANT domain, the frameshift mutations changed SANT domain structure, resulting in changes of amino acid sequence (Figure 2b) and three‐dimensional structure of encoding proteins (Figure S3). Finally, two independent homozygous lines *cr‐myb73‐35* and *cr‐myb73‐46* were selected for further studies.

Several mature seeds of wild‐type and mutants of T_1_ generation were randomly selected for phenotypic evaluation. Phenotypic analysis showed that the dehusked grains of ZH11 were transparent, while *cr‐myb73‐35* and *cr‐myb73‐46* displayed white‐belly chalky endosperm (Figure 2c). The milled grains of HZ were transparent, while the *cr‐HZ‐12* grains showed white‐belly chalky endosperm (Figure 2d). These results showed that loss of function in *OsMYB73* caused white‐belly chalky endosperm phenotype in both *indica* and *japonica* rice backgrounds. Morphologically, both wild‐type and mutant lines showed no significant differences in plant height and tiller number per plant (Figure 2j,k and Figure S4). Moreover, the mutation of *OsMYB73* significantly increased the grain length of the mutant lines compared to wild‐type (Figure 2e). From 3rd to 27th DAF, seeds were collected and photographed after every 3 days. The difference between wild‐type and mutant appeared about at 12–18 DAF. The grain filling rate of *cr‐myb73‐46* reduced significantly than that of wild‐type (ZH11), and the fresh weight of 1000 brown grains were also significantly lower than that of wild‐type (ZH11) (Figure 2f). In addition, due to the deficient filling process of abdominal endosperm of mutant seeds, an obvious white‐belly chalky endosperm phenotype occurred. Compared with wild‐type, the grain width and 1000‐grain weight of the mutants did not differ (Figure 2h,i).

### Effects of mutation in rice *OsMYB73* on compound starch granules and grain quality

To further analyse the structural changes in starch granules, we observed the transverse section of ZH11 and *cr‐myb73‐46* endosperm by scanning electron microscope (SEM) (Figure S5a,b). The structural arrangement of starch granules in the middle of *cr‐myb73‐46* endosperm cells were similar to those of wild‐type (homogeneous, compact and angular with few interstitial spaces) (Figure S5c,d). However, the abdominal starch granules of the mutants were loosely packed and separated by large gaps, and some starch granules became smaller, spherical or oval (Figure S5e,f). Meanwhile, the different amyloplasts structure were observed by transmission electron microscope (TEM). Compared with the wild‐type, the structure of amyloplasts in *cr‐myb73‐46* were scattered in the endosperm cells (Figure S5g,h) and the abdominal starch granules in *cr‐myb73‐46* mutant were much scattered in endosperm cells, that it could not form normal compound starch granules (Figure S5h). The above results indicated that *OsMYB73* may be involved in the formation of rice endosperm compound starch granules and its mutation changes morphological structure of starch granules and amyloplasts development. Loss function of rice *OsMYB73* reduced the uniformity of starch particles, potentially causing the grain obvious chalkiness (Figure S6).

**Figure 3:**
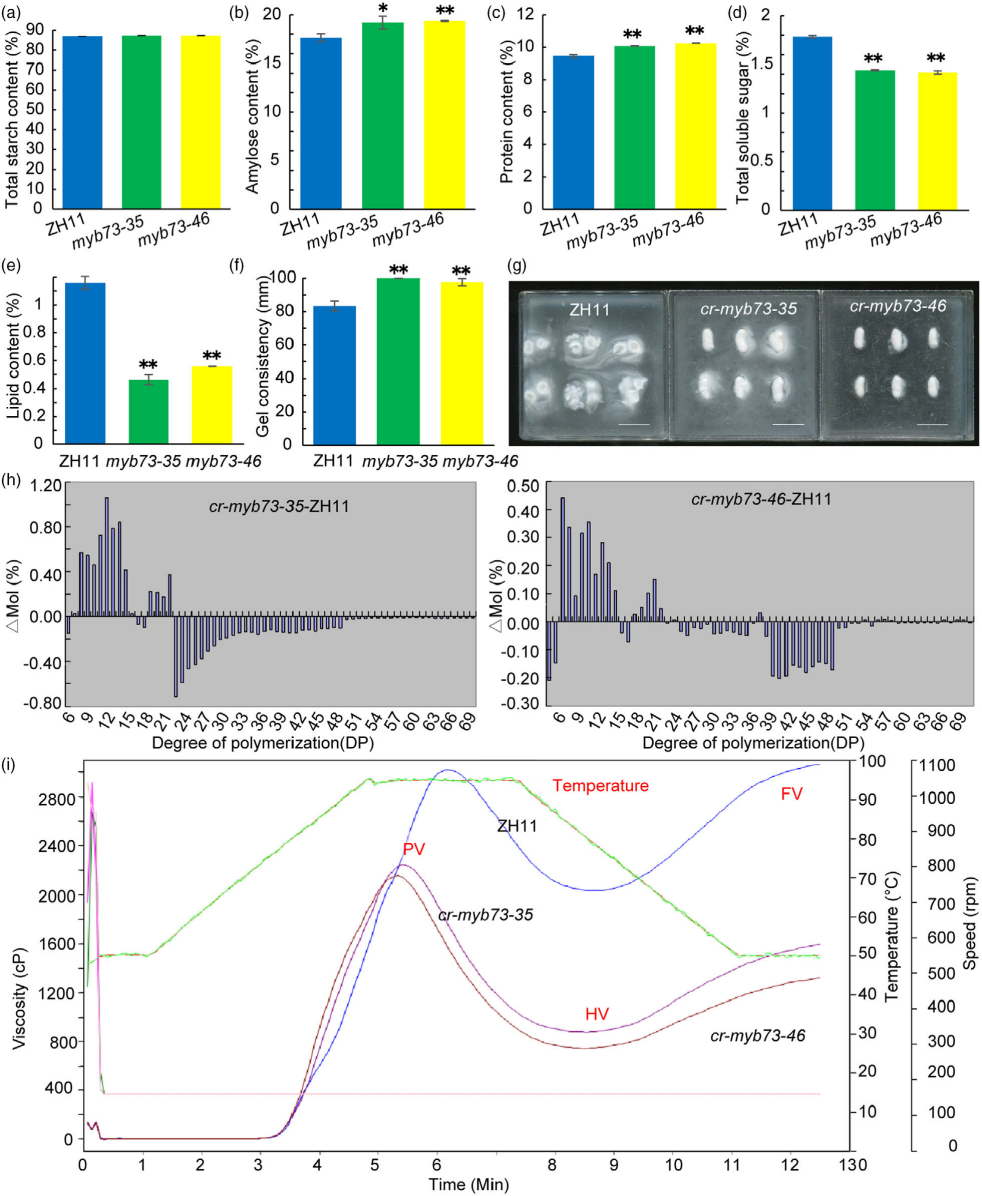
Grain starch physicochemical characteristics comparison of ZH11 and *cr‐myb73* in T_1_ generation. (a) Total starch content; (b) amylose content; (c) total protein content; (d) total soluble sugar content; (e) total lipid content; (f) gel consistency; (g) starch solubility in 1.7% KOH solution, scale bars are 1.0 cm; (h) chain length distributions of amylopectin in ZH11 and *cr‐myb73*; (i) pasting properties rapid visco analyser (RVA) of endosperm starch of ZH11 and *cr‐myb73*. FV, final viscosity; HV, hold through viscosity; PV, peak viscosity. The viscosity value at each temperature is the average of three replicates. The green line indicates the temperature changes during the measurements. Asterisks indicate statistical significance, as determined by a Student's *t‐*test (*P < 0.05, **P < 0.01).

Compared with ZH11, the total starch content in the seeds of two homozygous mutants *cr‐myb73‐35* and *cr‐myb73‐46* were not significantly different (Figure 3a). In addition, the amylose content and protein content of the *cr‐myb73‐35* and *cr‐myb73‐46* mutants were significantly higher compared with the wild‐type (Figure 3b,c). Furthermore, the soluble sugar content of two mutants were significantly lower compared with the wild‐type (Figure 3d). The total crude lipid content of *cr‐myb73‐35* and *cr‐myb73‐46* mutants were extremely low than that of wild‐type by 0.70% and 0.60%, respectively (Figure 3e), including the mentioned components (Figure S7 and Table S2).

The wild‐type and the two mutants were having soft gel consistency (≥61 mm), and the two mutants (*cr‐myb73‐35* and *cr‐myb73‐46*) mutants had a significant increase of about 16.67 mm and 14.33 mm compared to ZH11 (Figure 3f). Compared with wild‐type, the two mutants were more difficult to gelatinize in 1.7% KOH solution (Figure 3g) and ZH11 was estimated to be rated as Grade 6, and the mutants were rated as Grade 3. The *cr‐myb73‐35* and *cr‐myb73‐46* mutants amylopectin proportion of short and medium chains of DP 8–16 were increased, and the proportion of medium and long chains with DP ≥ 23 was decreased (Figure 3h). The starch viscosity characteristics were measured with a rapid viscosity analyser (RVA) and the results showed that *cr‐myb73‐35* and *cr‐myb73‐46* mutants had a similar viscosity curve trend to that of ZH11, but the viscosity value of mutants had a relatively low level, the peak viscosity of *cr‐myb73‐35* and *cr‐myb73‐46* mutants were only 74.18% and 71.15% of that of ZH11 (Figure 3i). The gelatinization temperature of wild‐type and mutants endosperm starch was compared, the results showed that To and Tp of endosperm starch of *cr‐myb73‐35* and *cr‐myb73‐46* were higher than that of ZH11, but Tc was not significantly different from that of ZH11 (Figure S8j), the gelatinization enthalpy (ΔH) of mutants starch were extremely significantly higher than that of ZH11 (Figure S8k). The solubility of starch granules in urea solution also reflects physicochemical properties, the white rice grain flour of ZH11 and *cr‐myb73‐35* were dissolved with different concentrations of urea (0–9 m), the results showed that they were difficult to dissolve in 0–4 m urea solution, but easy to dissolve in 5–9 m urea solution. The white rice flour of ZH11 began to dissolve at 5 m, but *cr‐myb73‐35* was difficult to dissolve, hence the significant difference in solubility appeared in 5–7 m concentration urea and ZH11 was easier to gelatinize than *cr‐myb73‐35* (Figure S8l,m). The above results indicated that rice *OsMYB73* mutation affects endosperm starch biosynthesis and physicochemical properties.

### Expression of rice grain size, endosperm starch biosynthesis, lipid transfer proteins and auxin biosynthesis‐related genes were affected in *cr‐myb73‐46* mutant

Analysis of transcriptomics data of ZH11 and *cr‐myb73‐46* seeds at 5 DAF showed that 124 genes were up‐regulated, and 575 genes were down‐regulated. A total of 496 transcripts were up‐regulated and 832 transcripts were down‐regulated. These genes were mainly involved in processes such as DNA binding and transcriptional regulation (Table S3). The results of scatter plot analysis of GO enrichment showed that *OsMYB73* was mainly involved in cell wall development (Figure S24), which related to rice cell division or proliferation to affect grain size. Besides, *OsMYB73* is also involved in responsing to heat (Figure S9). KEGG enrichment scatter plot analysis results showed that *OsMYB73* was predominantly involved in starch and sucrose metabolic pathways and endocytosis (Figure S10). The above results indicated that rice *OsMYB73* may play a transcriptional regulation role in starch metabolic and fatty acid degradation metabolic pathways (Figures S11 and S12) by combining with the promoters of downstream target genes. The qRT‐PCR method was used to detect the expression levels of 9 reported genes related to grain size, 7 genes related to lipid transport proteins and 27 genes related to endosperm starch synthesis. Compared with ZH11, the expression levels of 2 genes (*OsPPKL3* and *GSK2*) related to grain size were increased significantly in *cr‐myb73‐46* (Figure S13a), and the expression levels of 6 genes (*LTPL29*, *LTPL31*, *LTPL32*, *LTPL33*, *LTPL36 and LTPL147*) related to lipid transporters were significantly decreased significantly (Figure S13b). In addition, the expression level of small subunits of pyrophosphorylase *AGPS2a* and *AGPS2b* were significantly higher than those of the ZH11 (Figure S13c). Also, the expression level of auxin biosynthesis‐related genes (*OsYUC9*, *OsYUC10*, *OsYUC13* and *OsIAA29*) were significantly higher than those of the ZH11 (Figure S14). In summary, the *OsMYB73* mutation influenced the expression of genes related to grain size, starch and lipid biosynthesis and auxin biosynthesis.

### Loss of rice *OsMYB73* activity triggers changes in endosperm secondary metabolites

After finding out that loss of *OsMYB73* activity affects starch and lipid biosynthesis related genes expression in rice endosperm, the polar and nonpolar metabolites were extracted from the endosperm of wild‐type (ZH11) and mutant (*myb73‐35*) in order to compare broader metabolic profiles. Principal component analysis (PCA) revealed clear genotype‐specific differences in metabolic composition between wild‐type and mutants of three biological replicates. The first principal component (PC1) which is related to metabolic differences mostly in lipid, fatty acids, amino acids and derivatives, flavonoids, phenolic, organic acids, alkaloids, nucleotides and derivatives, terpenoids, quinones, tannins, lignans and coumarins identified 59.14% of total variance among genotypes, while PC2 highlighted 15.24% of total variance between genotypes (Figure S16). Venn diagram has showed differentially metabolites between wild‐type (ZH11) and mutant (*myb73‐35*) (Figure S17). The comparison of extracts measured by gas chromatography/mass spectrometry (GC/MS) revealed 12 classes of metabolites in endosperm tissue (Tables S4 and S5), which showed genotype‐specific differences in heat map and pathway analysis including purine and nicotinate metabolism (Figures S18–S20). Using wild‐type and mutants rice grain flour for extensive targeted metabolome analysis, the results showed that contents of main metabolites (lipids, amino acids and derivatives, flavonoids, phenolic acids, organic acids, alkaloids, nucleotides and derivatives, terpenoids, quinones, tannins, lignins and coumarins) in mutants endosperm were significantly lower than in the wild‐type (Figure S15). In conclusion, *OsMYB73* mutation triggered an extensive range of secondary metabolic processes in rice endosperm.

### Rice *OsMYB73* interacts with *OsNF‐YB1* are involved in transcriptional activation regulation of *OsISA2*, *OsLTPL36* and *OsYUC11* during endosperm storage substances accumulation

**Figure 4:**
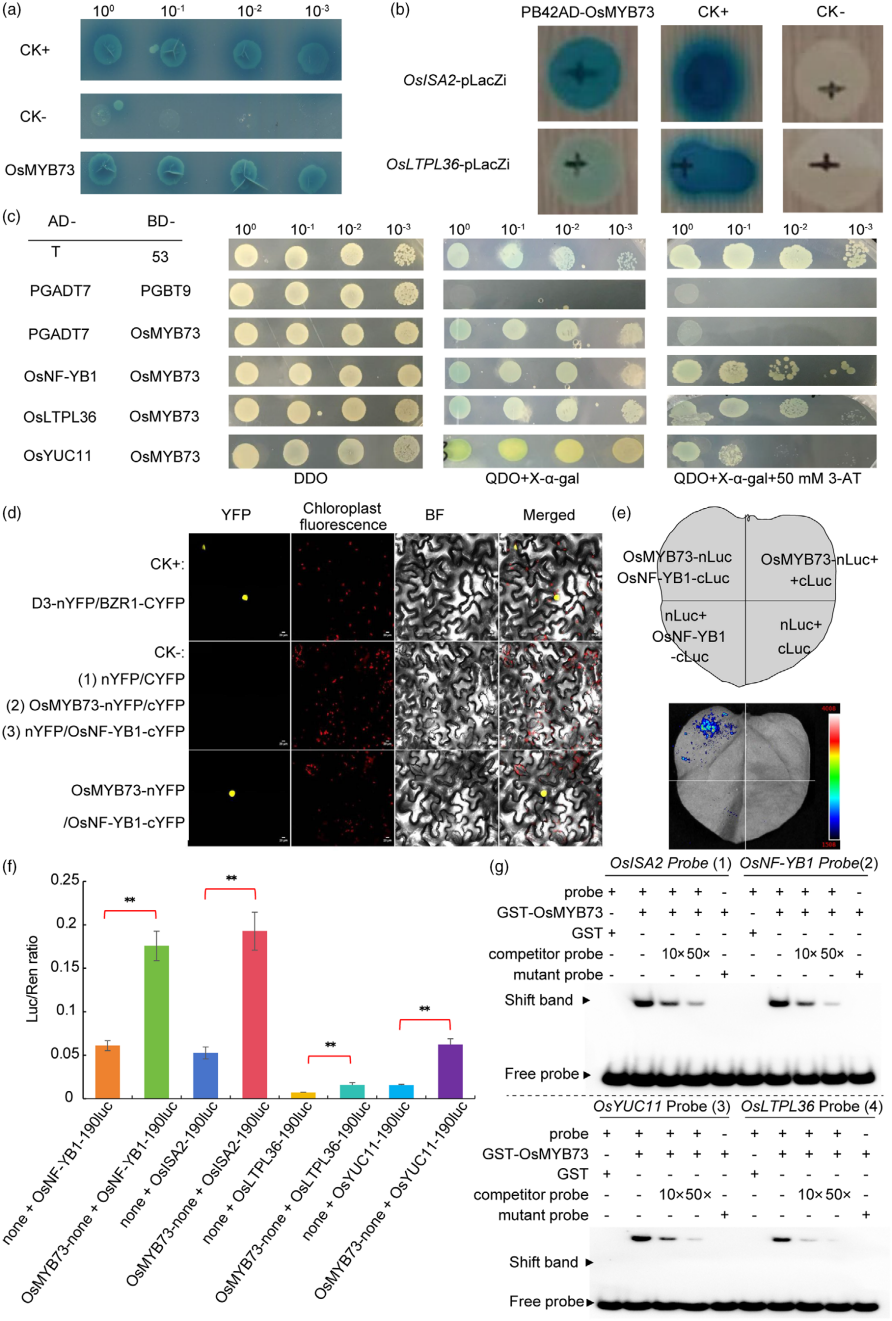
Yeast two‐hybrid and yeast one‐hybrid assays of rice OsMYB73. (a) Yeast two‐hybrid self‐activating phenomenon assay (scale bar is 2.0 cm); (b) yeast one‐hybrid assay showing *OsMYB73* binding *OsISA2* and *OsLTPL36* promoters; (c) yeast two‐hybrid assay showing the interaction between OsMYB73 and OsNF‐YB1, OsLTPL36 and OsYUC11 proteins in yeast cells; (d) bimolecular fluorescence complementation assay showing the interaction between OsMYB73 and OsNF‐YB1 proteins in tobacco leaf cells; (e) luciferase complementation imaging (LCI) assay showing that OsMYB73‐nLuc and OsNF‐YB1‐cLuc interact to form a functional LUC protein in Nicotiana benthamiana leaf cells. The combinations of nLuc and cLuc, nLuc and OsNF‐YB1‐cLuc and OsMYB73‐nLuc and cLuc were used as negative controls. (f) Analysis of the relationships between *OsMYB73* and *OsNF‐YB1*, *OsMYB73* and *OsISA2*, *OsMYB73* and *OsLTPL36*, *OsMYB73* and *OsYUC11* through luciferase reporter assay in the rice protoplasts. (g) EMSA of *OsMYB73* binding to *OsISA2*, *OsNF‐YB1*, *OsYUC11* and *OsLTPL36* promoters motifs.

In order to determine whether OsMYB73 has self‐activation in yeast, both the PGBT9‐*OsMYB73* and empty PGADT7 plasmid were transferred into yeast cells using two‐hybrid system. They were then observed in SD/‐Leu/‐Trp(DDO) + Kana and SD/‐Leu/‐Trp/‐Ade/‐His(QDO) + Kana + X‐α‐gal growth. PGADT7‐T plasmid and PGBKT7‐53 plasmid were co‐transformed as a positive control (CK+) and PGADT7 plasmid and PGBT9 plasmid were co‐transformed as a negative control (CK‐). It was found that the positive control grew not only on two deficient (DDO) + Kana yeast medium, but also grew normally on four‐deficient (QDO) + Kana + X‐α‐gal yeast medium; negative control grew on two‐deficient yeast medium (DDO) + Kana, but did not grow normally on four‐deficient (QDO) + Kana + X‐α‐gal yeast medium. The *OsMYB73* experimental group not only grew on two‐deficient yeast medium, but also observed normally on four‐deficient yeast medium (Figure 4a). The above results indicated that *OsMYB73* transcription factor has obvious self‐activation phenomenon in yeast. Futher, to find the downstream target genes regulated by *OsMYB73*, the yeast one‐hybrid (Y1H) system was used to determine the full‐length CDS of *OsMYB73* gene constructed on PB42AD vector. The *Wx (waxy)*, *OsAGPL2*, *OsAGPL3*, *SSIIa*, *SSIIIa*, *SSIVb*, *SBEI*, *SBEIIb*, *OsISA2 and OsLTPL36* promoters regions were constructed on pLacZi vector, and then co‐transformed to yeast cells. They were being cultured in SD‐Trp‐Ura + X‐gal two‐deficient medium to observe their growth. It was observed that only *OsMYB73*‐PB42AD and the co‐transformed *OsISA2*‐pLacZi and *OsLTPL36*‐pLacZi yeast cells grew on SD‐Trp‐Ura + X‐gal two‐deficient medium (Figure 4b), which indicated that *OsMYB73* could bind to *OsISA2* and *OsLTPL36* promoter regions to regulate rice endosperm amylopectin and lipid biosynthesis. Yeast two‐hybrid (Y2H), bimolecular fluorescence complementation (BIFC) and luciferase complementation imaging (LCI) assays showed that *OsMYB73* could interact with *OsNF‐YB1*, *OsLTPL36* and *OsYUC11* (Figure 4c *–*e), which indicated both *OsMYB73* and *OsNF‐YB1* may assist together to regulate rice endosperm starch and auxin biosynthesis. In summary, *OsMYB73* affects rice endosperm reserve substance accumulation by different metabolic pathways. Rice protoplasts were transformed with transcription factor *OsMYB73* and *proOsNF‐YB1::Luc*, *OsMYB73* and *proOsISA2::Luc*, *OsMYB73* and *proOsLTPL36::Luc*, *OsMYB73* and *proOsYUC11::Luc*, showing that *OsMYB73* may regulate target genes expression in rice by transcriptional activation (Figure 4f). Electrophoresis mobility shift (EMSA) assay showing that *OsMYB73* binds downstream target genes (*OsNF‐YB1*, *OsISA2*, *OsLTPL36* and *OsYUC11*) promoters GGTAGGT and CCGTTA motifs (Figure 4g).

### Rice *OsMYB73* double mutants display longer and chalky grains

**Figure 5:**
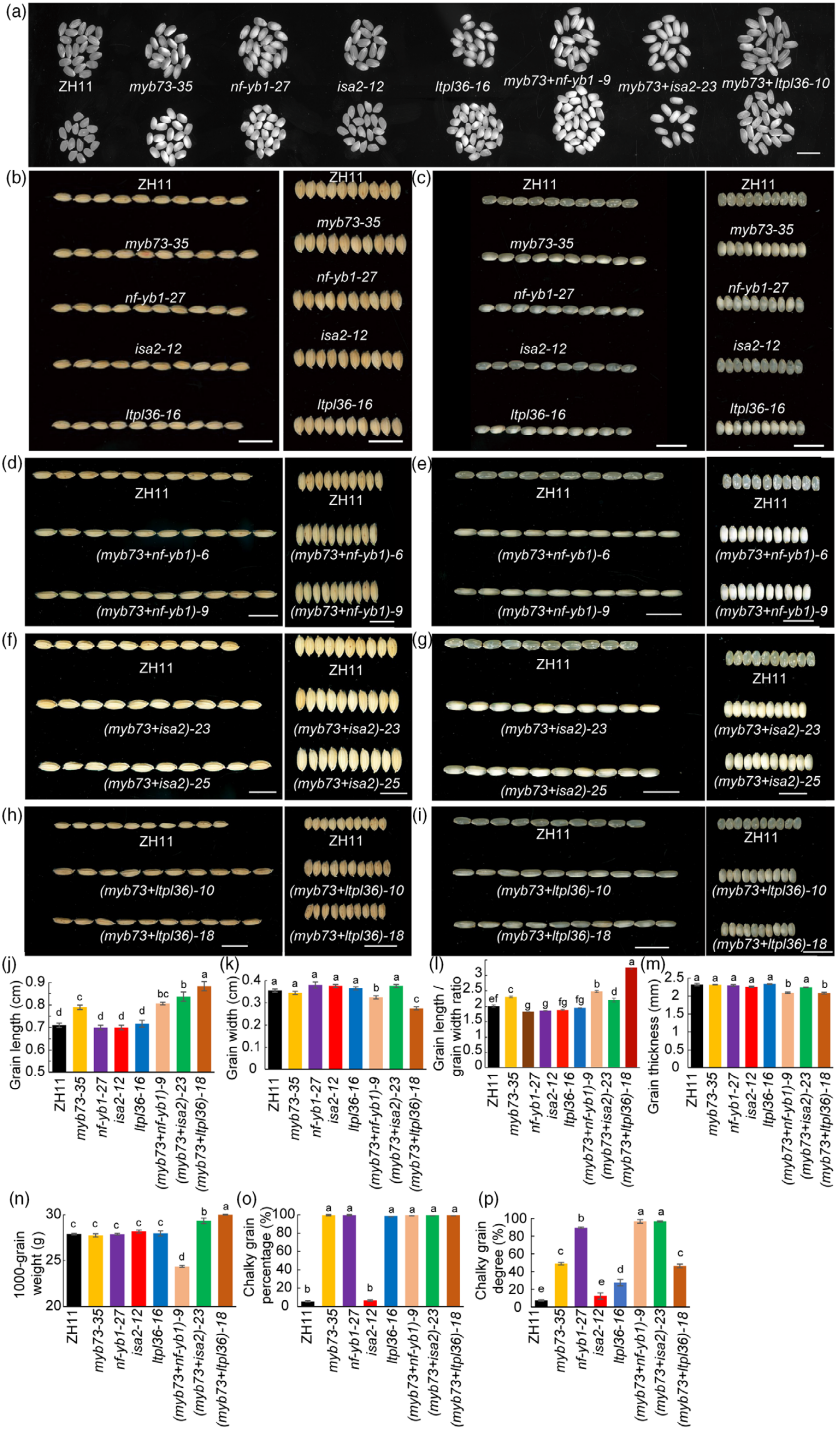
CRISPR/Cas9 mediated target mutagenesis of rice *OsMYB73*, *OsNF‐YB1*, *OsISA2*, *OsLTPL36*, *OsMYB73 + OsNF‐YB1*, *OsMYB73 + OsISA2* and *OsMYB73 + OsLTPL36* mutants grains phenotypic evaluation. Bar, 1.0 cm. Lowercase letters indicate statistically significant differences at *P <* 0.05 by one‐way ANOVA test with Tukey correction.

Rice *OsISA2* and *OsLTPL36* have similar expression pattern in the endosperm 5 DAF high co‐expression (Figure S21a), speculating that they have synergistic relationships in modulating endosperm development and storage substance accumulation. The *OsNF‐YB1* and *OsLTPL36* mutants were also observed portraying distinct chalky endosperm phenotype (Figure 5a), while *OsISA2* mutants showed transparent grain appearance (Figure 5a and Figure S21c). Moreover, we also obtained *OsMYB73* and other interacted proteins double mutants (*OsMYB73* + *OsNF‐YB1 and OsMYB73* + *OsISA2*, *OsMYB73* + *OsLTPL36*) to further investigate their seeds phenotype, *myb73 + nf‐yb1*, *myb73 + isa2* double mutants seeds displayed more severe chalky endosperm with longer grains (Figure 5d–g). *myb73 + ltpl36* double mutants seeds displayed belly chalky endosperm with longer grains (Figure 5h,i). The results showing that rice *OsMYB73* distinctly affects grain length and chalky grain degree (Figure 5j,p). *myb73 + nf‐yb1* double mutant severely reduced 1000‐grain weight which showed these are important to rice yield (Figure 5d,e,n).

### Screening of rice *OsMYB73* binding new motifs

Firstly, we used chip‐Seq to find other binding motifs and target genes, unfortunately, we did not find identified motif occuring in the promoters of *OsISA2*, *OsLTPL36* and *OsYUC11*, only to find 10 target genes of unknown function (Appendix S2). Besides, using *OsMYB73* gene constructed on pGADT7 vector as a bait, the yeast one‐hybrid promoter library was screened and the promoters that interacted with pGADT7‐*OsMYB73* were determined through gene detection, DNA sequencing and screening of positive clones. The results showed that some colonies at concentration of 40 mm 3‐AT; however, no colony was observed at concentration of 50 mm (Figure S22a), so 50 mm 3‐AT was then selected for subsequent screening. A total of 96 positive yeast clones were selected and amplified by PCR. Out of the 96 clones, only 49 produced bands and 20 were sequenced successfully. After sequence alignment, three promoter motifs with different sequences were finally obtained (GGTCAGCT, GCTCGGCA and GCTCGGTA). The three promoters screened by rotary validation were able to grow on SD‐TL, SD‐TLH and 50 mm 3‐AT SD‐TLH plates (Figure S22b). We did not find identified new motifs occurring in the promoters of *OsISA2*, *OsLTPL36* and *OsYUC11*, showing *OsMYB73* may involve in other biological pathways by binding different motifs.

### Overexpression of rice *OsMYB73* causes smaller and chalky grains

**Figure 6:**
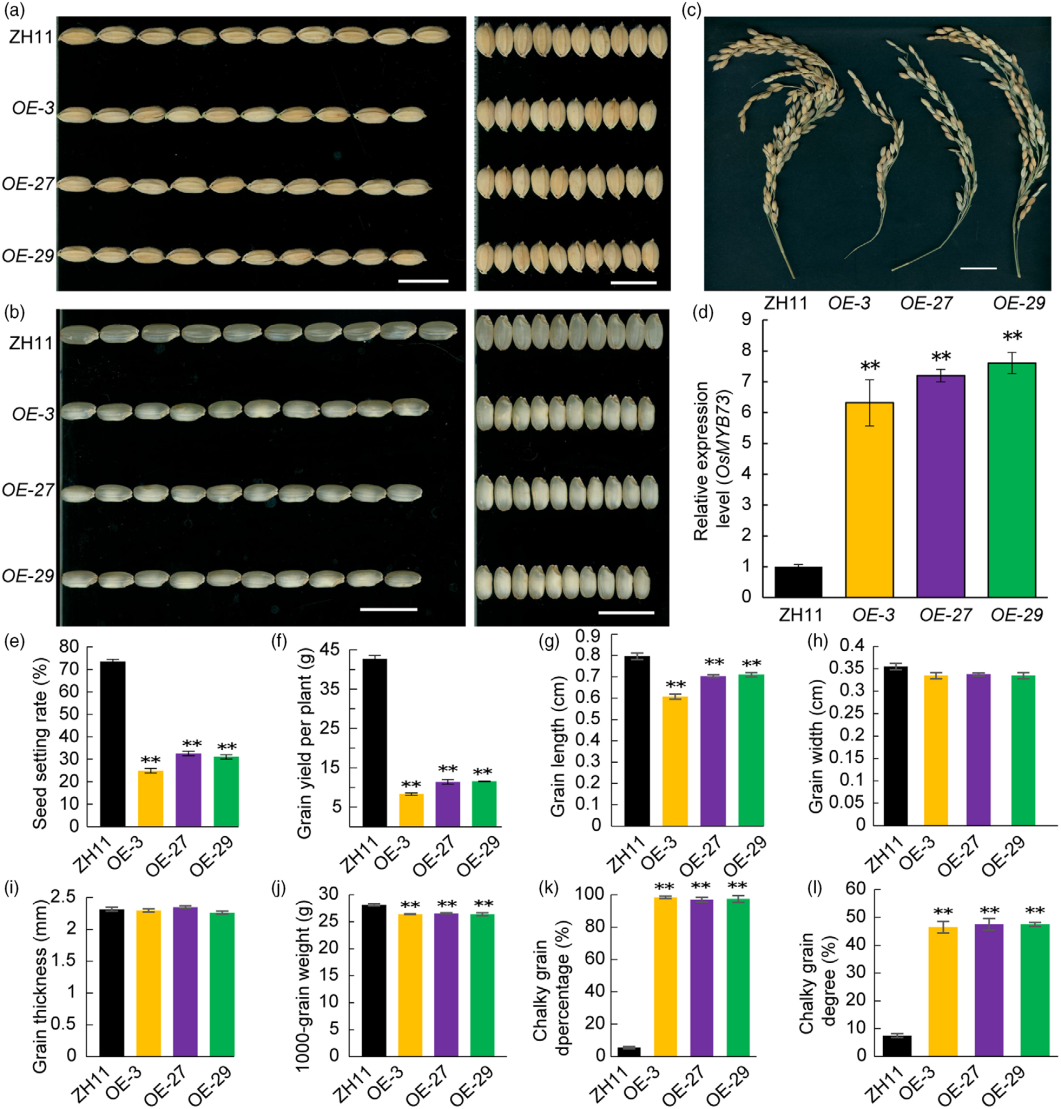
Overexpression of rice *OsMYB73* causes smaller and chalky grains. (a, b) Morphology of hulled (a) and dehulled (b) grains of Zhonghua11 (ZH11) and *OsMYB73* overexpression lines. Bars are 1.0 cm. (c) Panicle architecture of ZH11 and *OsMYB73* overexpression lines. Bar, 10 cm. (d) qRT‐PCR analysis of *OsMYB73* in ZH11 and transgenic lines endosperm at 5 DAF. (e) Seed setting rate of ZH11 and *OsMYB73* overexpression lines. (f) Grain yield per plant of ZH11 and *OsMYB73* overexpression lines. (g–i) Statistical analysis of grain length (g), grain width (h), grain thickness (i) of ZH11 and *OsMYB73* overexpression lines. (j) 1000‐grain weight of ZH11 and *OsMYB73* overexpression lines. Values are means ± SD, *n* = 3. *P < 0.05; **P < 0.01, determined by Student's *t*‐test.

In order to determine rice *OsMYB73* overexpression phenotype, the constructed vector was transformed into japonica rice variety Zhonghua 11 (ZH11) and qRT‐PCR was used to detect the expression levels of *OsMYB73* in both the wild‐type and transgenic plants. The results showed that expression level of *OsMYB73* in overexpressing transgenic lines *OE‐3*, *OE‐27* and *OE‐29* was significantly higher than that of ZH11 (Figure 6d). The seed setting rate of overexpression lines were significantly lower than that of ZH11 (Figure 6c), and the mature seeds showed smaller and chalky grains phenotype (Figure 6a,b), the chalky grain percentage and chalky grain degree of overexpression lines were significantly increased than that of ZH11 (Figure 6k,l). As reported earlier, chalkiness rate and chalkiness degree may increase significantly along with decrease in grain length‐width ratio, it was therefore speculated that *OsMYB73* overexpression seeds also showed core‐white endosperm phenotype, which may be caused by grain length decrease. The increased grain chalkiness may be resulted from changed grain length and grain width ratio (because of the change of grain length), *cr‐myb73‐35* knockout mutant line significantly increased and *OE‐3* overexpression line significantly decreased than wild‐type ZH11, respectively. We found that the grain length of overexpression transgenic lines decreased significantly as that of wild‐type because of smaller cells (Figure 6g and Figure S24), but showed no significant differences in grain width and thickness (Figure 6h,i). The 1000‐grain weight and yield of per plant in overexpression lines were reduced significantly compared with the wild‐type (Figure 6j,f). In a word, rice *OsMYB73* overexpression caused negative effects in many key agronomic traits.

### Multiple roles of rice *OsMYB73* in affecting grain size and chalkiness by regulating endosperm storage substances accumulation mediated auxin biosynthesis signalling pathways

SNP‐seek was performed on the 2381 bp *OsMYB73* DNA sequence using the rice 3K genome database (https://snpseek.irri.org). Only 10 SNPs were identified and a total of three haplotypes based on these variations were generated. Among these variations, four SNPs were located in the intron regions, four SNPs were located in the non‐genic regions and two SNPs occurred on exons of synonymous mutations (Appendix S2 and Table S6). Those non‐synonymous mutations were found in fewer varieties, and their functions and effects on yield and quality traits need to be further explored.

**FIGURE 7:**
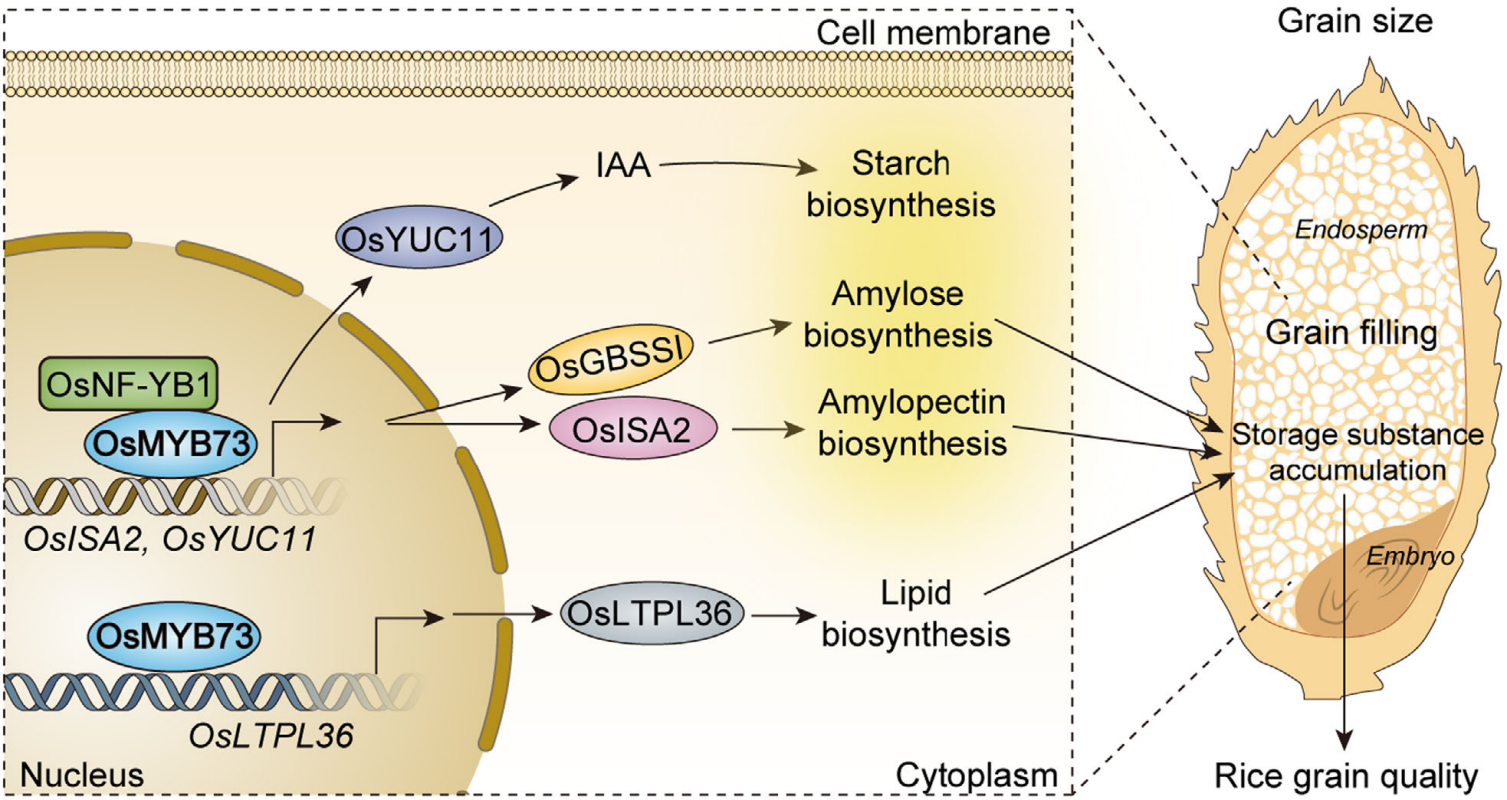
A schematic model of pleiotropic biological metabolic pathways of rice *OsMYB73. OsMYB73* may interact with nuclear factor *OsNF‐YB1* and bind to *OsISA2*, *OsLTPL36* and *OsYUC11* to regulate endosperm storage substances accumulation to affect rice grain quality.

Based on the above findings, we hypothesized a working model that rice *OsMYB73* is involved in a variety of important biological metabolic pathways. It not only affects rice grain size, but also regulates isoamylase *OsISA2* by interacting with *OsNF‐YB1* transcription factor in nucleus, thus affecting endosperm amylose and amylopectin biosynthesis. In addition, *OsMYB73* transcription factor affects endosperm lipid and auxin biosynthesis by binding to the promoter of lipid transporter gene *OsLTPL36* and *OsYUC11*. Rice *OsNF‐YB1* can bind the *OsYUC11* promoter to induce gene expression in vivo, both *osyuc11* and *osnf‐yb1* mutants exhibited reduced seed size and increased chalkiness, accompanied by reduction in indole‐3‐acetic acid biosynthesis (Xu *et al*., 2021). In summary, we speculate that rice *OsMYB73* and *OsNF‐YB1* paly synergistic pivotal role in simultaneously as a transcription activators to regulate grain filling and storage compounds accumulation to affect endosperm development and grain chalkiness through binding *OsISA2*, *OsLTPL36* and *OsYUC11* (Figure 7). This study revealed a key transcription factor regulatory mechanism that can be further utilized in rice yield and quality improvement.

## Discussion

Plant MYB is a large class of transcription factors which play crucial roles in many physiological and biochemical processes. Grain size and endosperm starch biosynthesis are important agronomic and appearance traits which affect yield and quality. It is necessary to identify more genes and quantitative trait loci (QTLs) controlling these traits. In this study, we used CRISPR/Cas9 gene editing technology to knock out a nucleus‐located transcription factor *OsMYB73* in rice. The *myb73* mutant seeds displayed a white‐belly chalky endosperm with increased grain length. In addition, knock‐out of *OsMYB73* negatively regulated grain length whereby the mutants showed longer grains compared to the wild‐type. Transcriptome sequencing and qRT‐PCR analysis showed that *OsMYB73* is mainly involved in cell wall development and its mutation altered the expression of some genes related to grain size, while overexpression significantly decreased the grain length but not grain chalkiness, according to RNA‐Seq data, *OsMYB73* is mainly involved in cell wall development, which related to cell division or proliferation to affect grain size. Also, the increased grain chalkiness may be resulted from changed grain length and grain width ratio (because of the change of grain length), *cr‐myb73‐35* knockout mutant line significantly increased and *OE‐3* overexpression line significantly decreased than wild‐type ZH11, respectively. To sum up, the change of grain size affecting seed development is the main reason, the change of endosperm storage substances accumulation affecting grain quality is the result. The *OsMAPK5*‐*OsWRKY72*‐*OsARF6* module negatively regulates grain length and grain weight‐mediated auxin signalling pathway in rice (Wang *et al*., 2024a). In further research, we will focus on grain size regulation mechanism research and *OsMYB73* may involve in other important biological processes.

In *OsMYB73* mutants, the grain filling rate decreased compared with the wild‐type, however, the increased grain length compensated for weight loss. Unluckily, mutants displayed chalky grains appearance quality. Balancing the trade‐off between rice yield and grain quality is crucial in rice breeding. In this study, the editing of *OsMYB73* resulted in increased grain length, however, the mutant grains showed enhanced chalkiness which compromised the grain quality. Yield is determined by a variety of agronomic traits and can improve individual traits to achieve high yield, while quality is determined by multiple traits influenced by complex and unstable climate environment, scientists are trying to elucidate grain size genetic molecular mechanism, combining with classical traditional breeding methods and gene editing technology to improve rice varieties with both high yield and superior quality (Ren *et al*., 2023). Further, intensive may uncover more genes modulating storage protein and starch synthesis to overcome the trade‐off effect between grain yield and quality (Cao *et al*., 2024). The mutant's abdominal chalky endosperm cells have small, spherical and ellipsoidal starch granules which are loosely arranged with large gap, some obvious defective starch granules become smaller in size and round, showing that *OsMYB73* may involve in rice endosperm development and compound starch granules formation. The physicochemical properties of endosperm starch of mutant seeds were significantly altered. Transcriptome sequencing analysis showed that it was involved in starch biosynthesis processes. Furthermore, *OsMYB73* mutation also affects the expression of genes related to lipid biosynthesis and fatty acid components. *OsMYB73* may affect the accumulation of stored substances within the grains by regulating the gene expression of lipid transporter *OsLTPL36*, thereby ultimately impacting the rice grain size. *OsOSC10* affects the development of rice grains by lipid biosynthesis pathways (Ma *et al*., 2024). Inactivation of *OsMYB73* triggers broad changes in secondary metabolites, which identifies its link to cooking quality of rice grain, or biotic and abiotic stress, providing some clues to improve rice nutritional qualities and resistance to stresses. Taken together, the rice *OsMYB73* transcription factor has pleiotropic effects on multiple important biological metabolic processes which regulate grain size and weight, endosperm starch, lipid, auxin and other metabolites biosynthesis to affect yield and quality in rice. By coincidence, scientists recently reported that *RGA1* is not only involved in grain size through G‐protein pathway but also in the regulation of rice quality and seed germination (Yang *et al*., 2024b). We therefore speculate that *OsMYB73* and *RGA1* may be involved in the same starch biosynthesis pathways ultimately the grain size.

In addition, more evidences are needed to prove *OsMYB73* binding downstream target genes new motifs (GGTCAGCT, GCTCGGCA and GCTCGGTA). Following the mutation of *OsMYB73*, *OsNF‐YB1* transcription factor and *Wx* genes expression levels increased, causing an increase in amylose content, thus indicating that *OsMYB73* transcription factor may negatively regulate amylose biosynthesis in rice endosperm. The kinase *OsSK41/OsGSK5* negatively regulates amylose content in rice endosperm by affecting the interaction between *OsEBP89* and *OsBP5* (Hu *et al*., 2023), speculating that *OsMYB73* and *OsSK41* may be involved in the same endosperm amylose synthesis metabolic pathway. Y2H and BIFC experiments confirmed *OsMYB73* and *OsNF‐YB1* transcription factors may cooperate in the direct regulation rice endosperm amylose biosynthesis. Our findings may provide more possible gene resources for regulating rice grain endosperm amylose by gene editing technology. Moreover, *OsNF‐YB1* can bind the *OsYUC11* promoter to induce auxin biosynthesis gene expression during endosperm development in rice, both *yuc11* and *nf‐yb1* mutants exhibited reduced seed size and increased chalkiness (Xu *et al*., 2021). Also, *RGB1* regulates grain development and starch accumulation through its effect on *OsYUC11*‐mediated auxin biosynthesis in rice endosperm cells (Zhang *et al*., 2021). In this study, we found that *OsMYB73* interacted with *OsYUC11* to affect rice grain filling and storage compounds accumulation (Figure 4). Previous studies suggested that *OsNF‐YB1* regulates endosperm development through multiple pathways, including transcriptional activation of sucrose transporter genes such as *OsSUT1*, *OsSUT3* and *OsSUT4* (Bai *et al*., 2016) and the endosperm amylose biosynthesis *wx* gene (Feng *et al*., 2022), OsNF‐YB1 function usually involves coordination with other TFs binding CCAAT motif (E *et al*., 2018; Xiong *et al*., 2019), we implied that *OsMYB73* is regulated by *OsNF‐YB1*. To test the hypothesis that *OsNF‐YB1* regulates the expression of *OsMYB73* in rice, we performed an electrophoretic mobility shift assay (EMSA) to detect the binding ability of *OsNF‐YB1* to the *OsISA2*, *OsMYB73* and *OsLTPL36* promoters in vitro. However, by surveying five probes spread across the promoter region, we failed to detect this binding ability, considering that OsNF‐YB1 usually form heterotrimers for their function (Figure S23a,b). We suppose that *OsNF‐YB1* transcription factor perform biological function need complex formation by binding CCAAA‐box of downstream target genes promoters, for example, *OsNF‐YB1* + *OsNF‐YC12* + *OsbHLH144* and *OsNF‐YB1* + *OsMADS14* to regulate *wx* and *OsAGPL2* gene during starch synthesis (Bello *et al*., 2019; Feng *et al*., 2022), *OsNF‐YB1* + *OsNF‐YC11/12* + *OsERF115* to regulate downstream target genes (Xu *et al*., 2016). *OsMADS1* + *OsNF‐YB1* + *OsNF‐YC12* affects rice grain thickness and quality through influencing monosaccharide loading (*OsMST4*) to endosperm (Liu *et al*., 2024a). Meanwhile, we also knocked out *OsMYB73* and *OsLTPL36* promoters CCAAT‐motif respectively, obtained *OsMYB73* mutants of defective grains phenotype, especially insertion C homozygous mutation (Figure S23c), *OsLTPL36* mutants of transparent grain appearance (Figure S23d), future studies should investigate agronomic traits influenced by *OsMYB73* and *OsLTPL36* promoters key motif mutation. Also, *OsMYB73* may bind *OsISA2* promoter by transcriptional activation to regulate amylopectin synthesis in rice endosperm and mutation of *OsMYB73* triggers an increase in expression levels of *OsISA2* gene further indicating that *OsMYB73* transcription factor may regulate amylopectin biosynthesis indirectly. *ISA2*‐suppressed mutant lines have no effect on grain appearance, but overexpression of *ISA2* causes shrivelled grains (Utsumi *et al*., 2011). The OsISA1 and OsISA2 hetero‐oligomer plays a predominant role in the amylopectin biosynthesis in rice endosperm although the homo‐oligomer can complement the function of the hetero‐oligomer at least to some extent (Utsumi and Nakamura, 2006). Although suppression of *OsISA2* gene expression caused the endosperm to have only the homo‐oligomer, no significant effects were detected on the starch phenotypes (Utsumi *et al*., 2011). In contrast, *OsISA2* overexpression led to endosperm having only the hetero‐oligomer, and starch synthesis in the endosperm was drastically impaired, both quantitatively and qualitatively (Utsumi *et al*., 2011). In this study, even although knock out *osisa2* mutants were performed, no chalky endosperm phenotype was observed in Figure 5a and Figure S21C, *OsMYB73* + *OsISA2* double mutants displayed severe chalky endosperm, showing that they have close interaction relationship to regulate endosperm amylopectin biosynthesis. We speculate other transcription factors may regulate *OsISA2* to affect rice endosperm amylopectin biosynthesis except *OsMYB73*. In the future research, we need more evidences to confirm this idea. Recently, OsLESV(FLO9)‐FLO6 as a non‐enzymatic molecular module responsible for OsISA1 localization on starch granules to affect rice endosperm amylopectin biosynthesis (Yan *et al*., 2024), we also enriched the genetic resources of rice endosperm amylopectin content regulation.

Moreover, *OsMYB73* regulates rice endosperm lipid synthesis by interacting with *OsLTPL36*. Previous studies revealed that down‐regulation expression of *OsLTPL36* caused chalky endosperm and resulted in reduced fat acid content. Therefore, the results of our study demonstrates that *OsMYB73*‐*OsLTPL36* interaction is involved in lipid biosynthesis pathway. Although, *OsMYB73* is involved in different biological metabolic pathways binding downstream target genes promoters motifs need further investigation. Interestingly, *OsMYB73*, *OsNF‐YB1*, *OsISA2* and *OsLTPL36* have similar expression pattern of 5 DAF rice endosperm highly co‐expression, speculating they have synergistic relationships in modulating endosperm development and storage substance accumulation. Moreover, we also obtained *OsMYB73* and other interaction proteins double mutants (*myb73 + nf‐yb1*, *myb73 + isa2 and myb73 + ltpl36*) seeds that displayed chalky endosperm with longer grains (Figure 5) to further investigate phenotype. Rice endosperm is a sensitive tissue, and deficiency or overexpression of key genes is not conducive to seed development. The promotion and inhibition mechanism between transcription factors *OsNAC25* and *OsNAC20/26* is crucial for maintaining stable expression of genes related to endosperm starch biosynthesis and normal starch accumulation (Wang *et al*., 2024b). OsSGL homeostasis is essential for starch synthesis and quality, both *OsSGL* RNAi and overexpression disrupts the starch biosynthetic pathway resulting in a chalky endosperm phenotype (Liu *et al*., 2022). Both *LCG1* knockdown and overexpression caused grain chalkiness in rice (Tu *et al*., 2024). In this study, both *OsMYB73* knockdown and overexpression resulted in a chalky endosperm phenotype, suggesting that it may keep balance in maintaining grain transparency in wild‐type rice.

Grain size and chalkiness influence appearance quality of rice grain. Reducing chalkiness is an important strategy towards the production of improved quality. Our study findings not only provide a novel genetic resource for molecular improvement of rice yield and quality but also reveals new genetic roles of rice MYB transcription factor family in regulation of seed development. In a recent study, improving rice grain shape and quality through upstream open reading frame editing‐mediated *GW7‐uORF* and *GLW7‐uORF* translation regulation (Yang *et al*., 2024a). Also, we try to generate targeted mutagenesis of rice *OsMYB73* in non‐coding regions to regulate downstream genes expression subtly, expecting keeping the longer grains and less chalkiness. Every coin has two sides, Japanese modern rice breeding has developed two different types of rice, eating and sake‐brewing rice, with different grain characteristics. Increases the chalkiness frequency in the endosperm, a desirable trait for sake brewing but decreases the grain appearance quality (Yoshida *et al*., 2024). Inactivation of *OsMYB73* triggers broad changes in secondary metabolites, we suppose that *OsMYB73* overexpression may increase secondary metabolites to affect rice taste quality, next, we also will measure rice taste quality of wild‐type and mutants. Lastly, we predicted *OsMYB73* interacted proteins through STRING website, showing that rice *OsMYB73* may play a role in other biotic and abiotic biological process (heat, cold, salt, insect, heavy metal and so on) (Figures S25–S33), *OS01T0855400‐00* is *OsMYB73*. RNA‐Seq data showing rice *OsMYB73* responsing to heat (Figure S9). Although we established that *OsMYB73* modulates rice starch and lipid biosynthesis, and auxin biosynthesis, further studies on molecular regulation mechanism of seed development and other biotic and abiotic biological processes need to be carried out.

## Materials and methods

### Plant materials and growth conditions

T_0_ and T_1_ generations of *cr‐myb73* transgenic lines generated using the CRISPR/Cas9 system and wild‐type were grown under natural conditions in the fields (See Appendix S1 for detailed steps).

### RNA extraction and quantitative real‐time PCR (qRT‐PCR) analysis

To investigate the expression of genes associated to grain shape, starch and lipid biosynthesis and auxin biosynthesis total RNA was extracted from 5 DAF seeds by using the Trizol reagent. The gene‐specific primers related to starch synthesis were those described by She *et al* (She *et al*., 2010). The *Ubiquitin* gene was used as an internal control, and the relative expression level was calculated by the $2^{-\Delta \Delta C_{T}}$ method (see Appendix S1 for detailed steps).

### Histochemical GUS staining

The putative promoter region of *OsMYB73* (~2 kb upstream of ATG) was amplified by PCR and cloned into the *EcoR*I/*Nco*I sites of pCAMBIA1305. Tissues were submerged in histochemical GUS staining solution (see Appendix S1 for detailed steps).

### Subcellular localization

The rice *OsMYB73* ORF without a termination codon was constructed into the PAN580‐GFP vector. The fusion constructs and an empty control vector were transformed into rice protoplasts separately. GFP fluorescence signals was detected using a confocal laser scanning microscope (see Appendix S1 for detailed steps).

### Knockout rice *OsMYB73* through CRISPR/Cas9 gene editing system

The target site (GCCATGGCTACCGTTCCAGT) in the first exon of rice *OsMYB73* was designed via CRISPR direct website (Naito *et al*., 2015). T_0_ generation transgenic plants were determined the positive plants by testing hygromycin gene (Table S1) (see Appendix S1 for detailed steps).

### Microscopy observation

The brown rice of ZH11 wild‐type and the *cr‐myb73* mutants were cut transversely and vertically with the a sharp blade, and the ruptured transverse surface was coated with gold to prepare samples according to Kang *et al*. (2005) (see Appendix S1 for detailed steps).

### Analysis of starch physicochemical characteristics in mature rice grains

The total starch content of the milled rice powder was measured using a starch assay kit. Amylose content was determined following the method depicted by Liu *et al*. (2009). Lipid and protein contents in the grains were measured according to the method described by Kang *et al*. (2005). The swelling and gelatinization properties of endosperm starch in urea solution were measured according to the method described by Nishi *et al*. (2001). Total soluble sugar content was quantified using the phenol‐sulphuric acid method (Nishi *et al*., 2001). Thermal characteristics were measured with a modulated differential scanning calorimeter as described by Kweon *et al*. (2000) with minor modifications. All assays were done with three biological replicates (see Appendix S1 for detailed steps).

RNA‐Seq (see Appendix S1 for detailed steps).
Metabonomics analysis (see Appendix S1 for detailed steps).
Yeast two‐hybrid assays (see Appendix S1 for detailed steps).
Yeast one‐hybrid assays (see Appendix S1 for detailed steps).
Dual‐luciferase reporter assay for transactivation analysis (see Appendix S1 for detailed steps).
ChIP experiment and high‐throughput sequencing and data analysis (see Appendix S1 for detailed steps).
Transcription factor (TF)‐centred yeast one‐hybrid (Y1H) assay (see Appendix S1 for detailed steps).

### Determination of the novel motifs recognized by rice *OsMYB73*

A 7 bp random motif library from yeast strain Y187 was purchased from Nanjing Ruiyuan Biotechnology Co., Ltd. (Nanjing, China) (See Appendix S1 for detailed steps).

### Bimolecular fluorescence complementation (BiFC) assay

Two pairs of constructs, *OsMYB73*‐VN173 and *OsNF‐YB1*‐VC155, were transformed into tobacco leaf cells. The mixture of modified pUC‐SPYNE and pUC‐SPYCE vector was used as a negative control. After incubation overnight in the dark at 25 °C, the fluorescence was observed using a FV1000 MP two‐photon laser scanning fluorescence microscope (Olympus, Tokyo, Japan) (see Appendix S1 for detailed steps).

### Promoters analysis and motifs prediction

The promoters sequences 2.0 kb upstream of the translationstart sites of candidate genes were downloaded from the EnsemblPlants website (http://plants.ensembl.org/). The *OsNF‐YB1* binding sites (CCAAT‐box) were identified using PLANTPAN v.3.0 (http://plantpan.itps.ncku.edu.tw/) and NEW PLACE (http://www.dna.affrc.go.jp/PLACE/). The *OsMYB73* binding sites (GGTAGGT and CCGTTA) were identified using JASPAR website (https://jaspar.elixir.no/).

### Purification of tag‐fused proteins

For the recombinant protein expression, the CDS of *OsNF‐YB1* and *OsMYB73* were amplified and cloned into pET28a and pGEX‐4T‐1, respectively. HIS‐OsNF‐YB1 and GST‐OsMYB73 recombinant proteins were induced in *E. coli* strain Rossetta and purified by Glutathione‐Sepharose Resin Protein Purification Kit and 6 X His‐Tagged Protein Purification Kit, respectively.

### Electrophoresis mobility shift (EMSA) assay

Electrophoresis mobility shift assay according to EMSA Kit (see Appendix S1 for detailed steps).

## Accession numbers

The name and ID for the rice genes mainly mentioned in this study are as follows: *OsMYB73* (*LOC_Os01g63680/Os01g0855400*), *OsNF‐YB1* (*LOC_Os02g49410/Os02g0725900*), *OsISA2* (*LOC_Os05g32710/Os05g0393700*), *OsLTPL36* (*LOC_Os03g25350/Os03g0369100*) and *OsYUC11* (*LOC_Os12g08780*/*Os12g0189500*).

## Conflict of interest

The authors declare no competing interest.

## Author contributions

S.T., P.H., S.L., G.S., X.W., G.R. and F.G. designed research. S.L., J.W., R.C., Y.C, S.F. and Z.D. performed research. G.S., S.T., P.H., G.R., F.G., X.W., J.W., G.J., J.Z., Y.W., R.C., L.X., Z.S., S.H., S.L., Y.L., F.L., Y.C., S.F., A.M.M, J.T. and Z.D. provided technical assistance and analysed data. S.L., G.S., A.M.M, P.H., S.T., G.R., F.G., X.W., J.W., R.C., S.F. and J.T. wrote and revised the manuscript.

## Supporting information


**Figure S1** Phylogenetic analysis and protein domain prediction of rice OsMYB73.
**Figure S2** The expression profile of rice OsMYB73.
**Figure S3** Comparison of the amino acid sequence and three‐dimensional models between ZH11 and the mutants' version in lines cr‐myb73‐35 and cr‐myb73‐46.
**Figure S4** Comparison of rice plant morphology between wild‐type (ZH11) and mutants (cr‐myb73‐35 and cr‐myb73‐46).
**Figure S5** Scanning and transmission electron microscopy images of wild‐type (ZH11) and mutant (cr‐myb73‐46) in T1 generation.
**Figure S6** The rice starch particles between ZH11 and mutants (cr‐myb73‐35 and cr‐myb73‐46).
**Figure S7** Composition of medium‐ and long‐chain fatty acid in rice mature grains between wild‐type (ZH11) and mutants (cr‐myb73‐35 and cr‐myb73‐46).
**Figure S8** Rice grain starch physicochemical characteristics comparison of wild‐type (ZH11) and mutants (cr‐myb73‐35 and cr‐myb73‐46) in T1 generation (Continued Figure 3).
**Figure S9** RNA‐sequencing transcriptomic analysis of ZH11 and cr‐myb73‐46 (cr‐myb73‐46 vs. ZH11 GO enrichment scatterplot).
**Figure S10** RNA‐sequencing transcriptomic analysis of ZH11 and cr‐myb73‐46 (cr‐myb73‐46 vs. ZH11 KEGG enrichment scatterplot).
**Figure S11** RNA‐sequencing transcriptomic analysis of ZH11 and cr‐myb73‐46 (cr‐myb73‐46 vs. ZH11 starch and sucrose metabolic pathway).
**Figure S12** RNA‐sequencing transcriptomic analysis of ZH11 and cr‐myb73‐46 (cr‐myb73‐46 vs. ZH11 fatty acid degradation pathway).
**Figure S13** The relative expression level of rice grain shape, lipid transport protein and endosperm starch synthesis‐related genes of wild‐type (ZH11) and mutant (cr‐myb73‐46) at 5 DAF in the seeds.
**Figure S14** The relative expression level of rice auxin biosynthesis, trehalose‐6‐phosphate synthase (TPS) and sucrose synthase (SUS)‐related genes of wild‐type (ZH11) and mutant (cr‐myb73‐46) at 5 DAF in the seeds.
**Figure S15** Heat map of metabolites comparation in rice endosperm between wild‐type (ZH11) and mutants (myb73‐35). ‐1, −2 and − 3 are three biological replicates of wild‐type and mutants.
**Figure S16** Principal component analysis (PCA) of polar and non‐polar metabolites identified in rice endosperm.
**Figure S17** Venn diagram showing differential metabolites.
**Figure S18** ZH11_vs_myb73‐35 KEGG barplot of differentially metabolites classification.
**Figure S19** ZH11_vs_myb73‐35 KEGG enrichment of differentially metabolites.
**Figure S20** Peak‐related genes annotation of GO and KEGG.
**Figure S21** Rice OsISA2 spatiotemporal expression pattern and CRISPR/Cas9‐mediated target mutagenesis of OsISA2‐mutant brown rice grains phenotypic evaluation in T1 generation.
**Figure S22** Screening binding motifs of rice OsMYB73 (TF‐centred Y1H).
**Figure S23** In vitro analysis of binding and activation ability of rice OsNF‐YB1 to OsMYB73, OsISA2 and OsLTPL36 and knock out OsMYB73 and OsLTPL36 promoters CCAAT‐motif brown rice phenotypic evaluation.
**Figure S24** Microscope scanning of the glume outer surfaces of ZH11 and cr‐myb73‐46, OE‐myb73‐3 transgenic rice mature seeds.
**Figures S25–S33** The prediction of rice OsMYB73 interacted proteins and involved in other biotic and abiotic biological processes.

**Table S1** The primers used in this study.

**Table S2** Absolute values of all detected fatty acid components (μg/g, dry weight) in seeds from OsMYB73 mutants lines (myb73‐35, myb73‐46) and wild‐type (ZH11).

**Table S3** myb73 VS ZH11_Gene_differential_expression.

**Table S4** List of differentially metabolites in ZH11_vs_myb73‐35_filter.

**Table S5** List of differentially metabolites in ZH11_vs_myb73‐46_filter.

**Table S6** Rice OsMYB73 haplotype analysis (Excel).

**Appendix S1** The detailed steps of the methods involved in this study.

**Appendix S2** Rice OsMYB73‐chip‐Seq data and haplotype analysis (PDF file).

## Data Availability

The data that support the findings of this study are available in the supplementary and appendix material of this article.
