# Supplementary material for: A novel transcription factor OsMYB73 affects grain size and chalkiness by regulating endosperm storage substances' accumulation‐mediated auxin biosynthesis signalling pathway in rice
Source: Plant Biotechnol J. 2024 Dec 26;23(4):1021–38. doi: 10.1111/pbi.14558 (PMC11933829; doi:10.1111/pbi.14558)
Supplement: Supplementary file 1 — Figure S1 Phylogenetic analysis and protein domain prediction of rice OsMYB73. Figure S2 The expression profile of rice OsMYB73. Figure S3 Comparison of the amino acid sequence and three‐dimensional models between ZH11 and the mutants' version in lines cr‐myb73‐35 and cr‐myb73‐46. Figure S4 Comparison of rice plant morphology between wild‐type (ZH11) and mutants (cr‐myb73‐35 and cr‐myb73‐46). Figure S5 Scanning and transmission electron microscopy images of wild‐type (ZH11) and mutant (cr‐myb73‐46) in T1 generation. Figure S6 The rice starch particles between ZH11 and mutants (cr‐myb73‐35 and cr‐myb73‐46). Figure S7 Composition of medium‐ and long‐chain fatty acid in rice mature grains between wild‐type (ZH11) and mutants (cr‐myb73‐35 and cr‐myb73‐46). Figure S8 Rice grain starch physicochemical characteristics comparison of wild‐type (ZH11) and mutants (cr‐myb73‐35 and cr‐myb73‐46) in T1 generation (Continued Figure 3). Figure S9 RNA‐sequencing transcriptomic analysis of ZH11 and cr‐myb73‐46 (cr‐myb73‐46 vs. ZH11 GO enrichment scatterplot). Figure S10 RNA‐sequencing transcriptomic analysis of ZH11 and cr‐myb73‐46 (cr‐myb73‐46 vs. ZH11 KEGG enrichment scatterplot). Figure S11 RNA‐sequencing transcriptomic analysis of ZH11 and cr‐myb73‐46 (cr‐myb73‐46 vs. ZH11 starch and sucrose metabolic pathway). Figure S12 RNA‐sequencing transcriptomic analysis of ZH11 and cr‐myb73‐46 (cr‐myb73‐46 vs. ZH11 fatty acid degradation pathway). Figure S13 The relative expression level of rice grain shape, lipid transport protein and endosperm starch synthesis‐related genes of wild‐type (ZH11) and mutant (cr‐myb73‐46) at 5 DAF in the seeds. Figure S14 The relative expression level of rice auxin biosynthesis, trehalose‐6‐phosphate synthase (TPS) and sucrose synthase (SUS)‐related genes of wild‐type (ZH11) and mutant (cr‐myb73‐46) at 5 DAF in the seeds. Figure S15 Heat map of metabolites comparation in rice endosperm between wild‐type (ZH11) and mutants (myb73‐35). ‐1, −2 and − 3 are three [file PBI-23-1021-s009.doc]

**Supporting Information for**

**A transcription factor *OsMYB73* affects grain quality by regulating endosperm storage substances accumulation mediated auxin biosynthesis signaling pathway in rice**

Song Liu^a,b,1^, Gaoneng Shao^a,1^, Amos Musyoki Mawia^a,1^, Xiangjin Wei^a,1^, Jiamin Wu^a,1^, Ruijie Cao^a,1^, Guiai Jiao^a^, Yawen Wu^a^, Jian Zhang^a^, Lihong Xie^a^, Zhonghua Sheng^a^, Shikai Hu^a^, Sanfeng Li^a^, Yusong Lv^a^, Feifei Lu^a^, Yujuan Chen^a^, Sajid Fiaz^a^, Javaria Tabassum^a^, Zhimin Du^a^, Fangyuan Gao^b^,*, Guangjun Ren^b^,*, Gaoneng Shao^a^,*, Peisong Hu^a^,*, Shaoqing Tang^a^,*

^a^State Key Laboratory of Rice Biology (State Key Laboratory of Rice Biology and Breeding), China-IRRI Joint Research Center on Rice Quality and Nutrition, Key Laboratory of Rice Biology and Genetics Breeding of [Ministry](../../../../../Program%20Files%20(x86)/Youdao/Dict/8.5.3.0/resultui/html/index.html#/javascript:;) [of](../../../../../Program%20Files%20(x86)/Youdao/Dict/8.5.3.0/resultui/html/index.html#/javascript:;) [Agriculture](../../../../../Program%20Files%20(x86)/Youdao/Dict/8.5.3.0/resultui/html/index.html#/javascript:;), China National Center for Rice Improvement, China National Rice Research Institute, Chinese Academy of Agricultural Sciences, Hangzhou 311401, China

^b^Environment-friendly Crop Germplasm Innovation and Genetic Improvement Key Laboratory of Sichuan Province, Key Laboratory of Tianfu Seed Industry Innovation (Co-construction by [Ministry](../../../../../Program%20Files%20(x86)/Youdao/Dict/8.5.3.0/resultui/html/index.html#/javascript:;) and Province), [Ministry](../../../../../Program%20Files%20(x86)/Youdao/Dict/8.5.3.0/resultui/html/index.html#/javascript:;) [of](../../../../../Program%20Files%20(x86)/Youdao/Dict/8.5.3.0/resultui/html/index.html#/javascript:;) [Agriculture](../../../../../Program%20Files%20(x86)/Youdao/Dict/8.5.3.0/resultui/html/index.html#/javascript:;) and Rural Affairs, Crop Research Institute (Sichuan Provincial Germplasm Center), Sichuan Academy of Agricultural Sciences, Chengdu 610066, China

^1^These authors (S.L., G.S., A.M.M, X.W., J.W., and R.C.) contributed equally to this work

*To whom correspondence may be addressed.

*Shaoqing Tang

**Email:** [tangshaoqing@caas.cn;](mailto:tangshaoqing@caas.cn;) shaoqingtangcnrri@126.com.

*Peisong Hu

**Email:** [hupeisong@caas.cn](mailto:hupeisong@caas.cn;); peisonghu@126.com.

*Gaoneng Shao

**Email:** shaogaoneng@caas.cn.

*Guangjun Ren

**Email:** guangjun61@sina.com.

*Fangyuan Gao

**Email:**gfy246@163.com; gaofangyuan@scsaas.cn.

**Supplementary Figures and Tables**


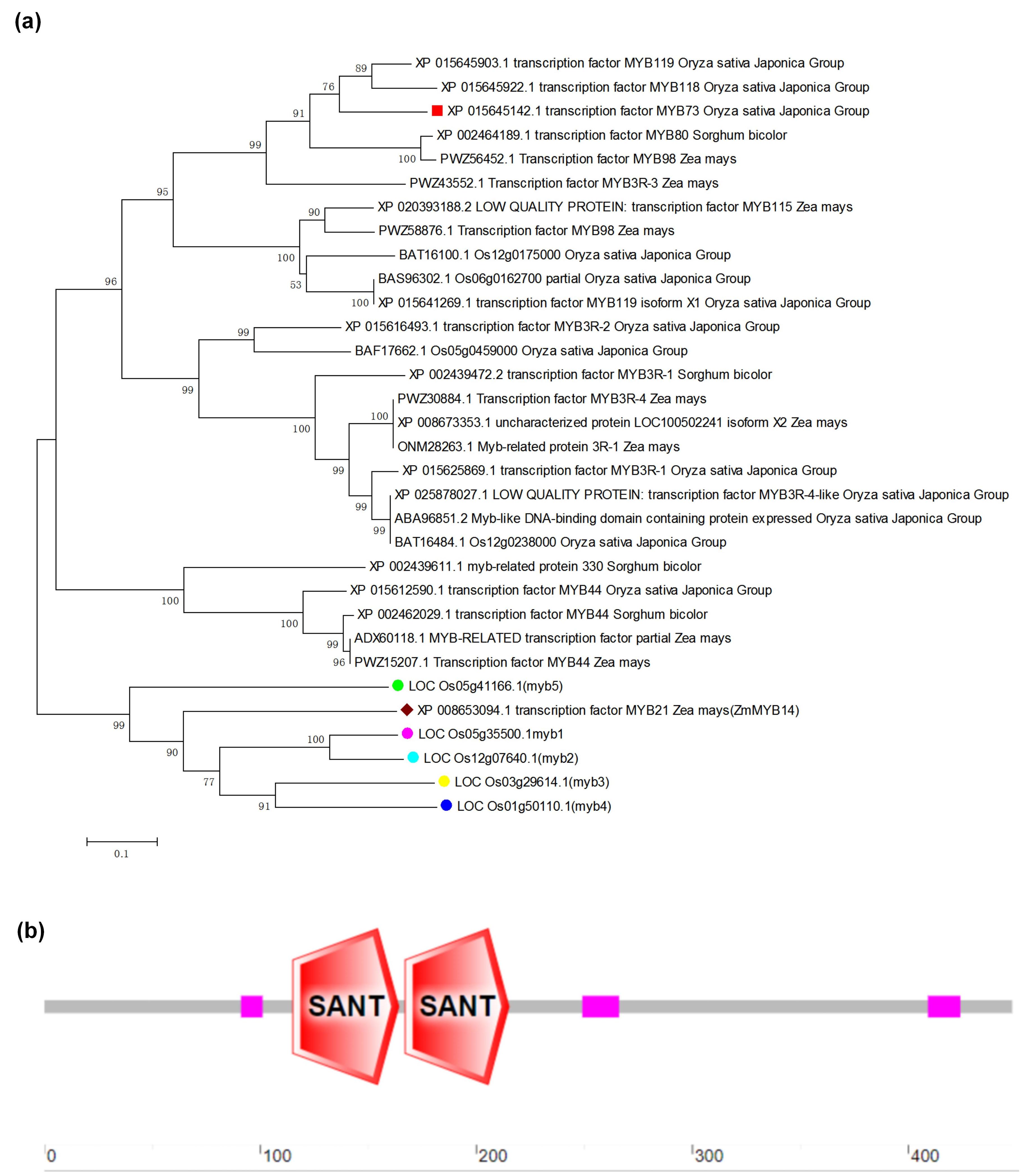


**Figure S1:** **Phylogenetic analysis and protein domain prediction of OsMYB73.**

**
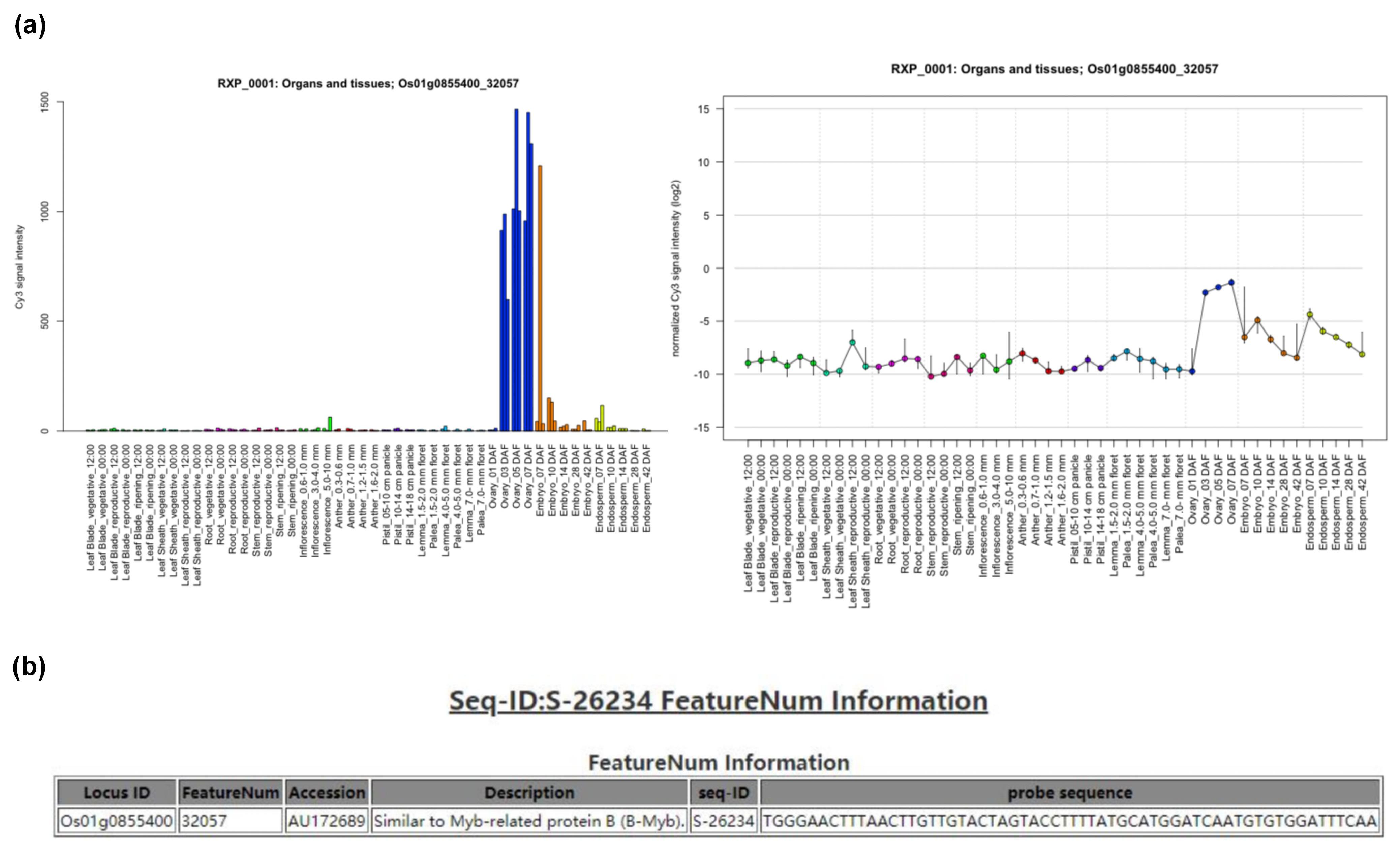
**

**Figure S2: The expression profile of *OsMYB73*.**

The data for spatio-temporal expression pattern of *OsMYB73* (*LOC_Os01g63680*/*Os01g0855400*) was downloaded from RiceXPro (http://ricexpro.dna.affrc.go.jp) database.

***
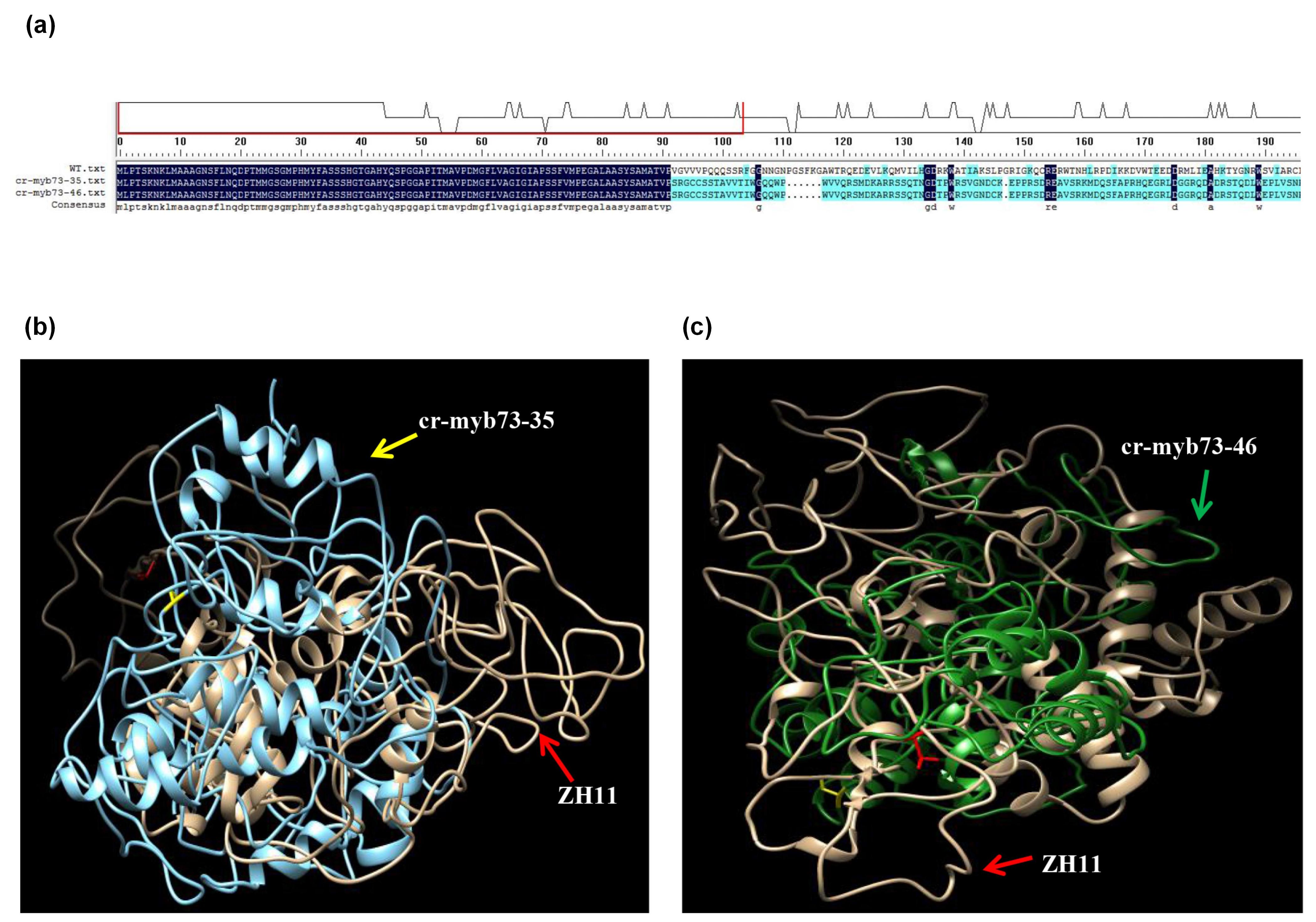
***

**Figure S3:** **Comparision of the amino acid sequence and three-dimensional models between ZH11 and the mutants version in lines *cr-myb73-35*、*cr-myb73-46*.**

1. Alignment of the ZH11 amino acid with the mutants version from lines *cr-myb73-35*, *cr-myb73-46*. (b) Predicted three-dimensional (3D) protein structure comparison of ZH11 and cr-myb73-35, cr-myb73-46.


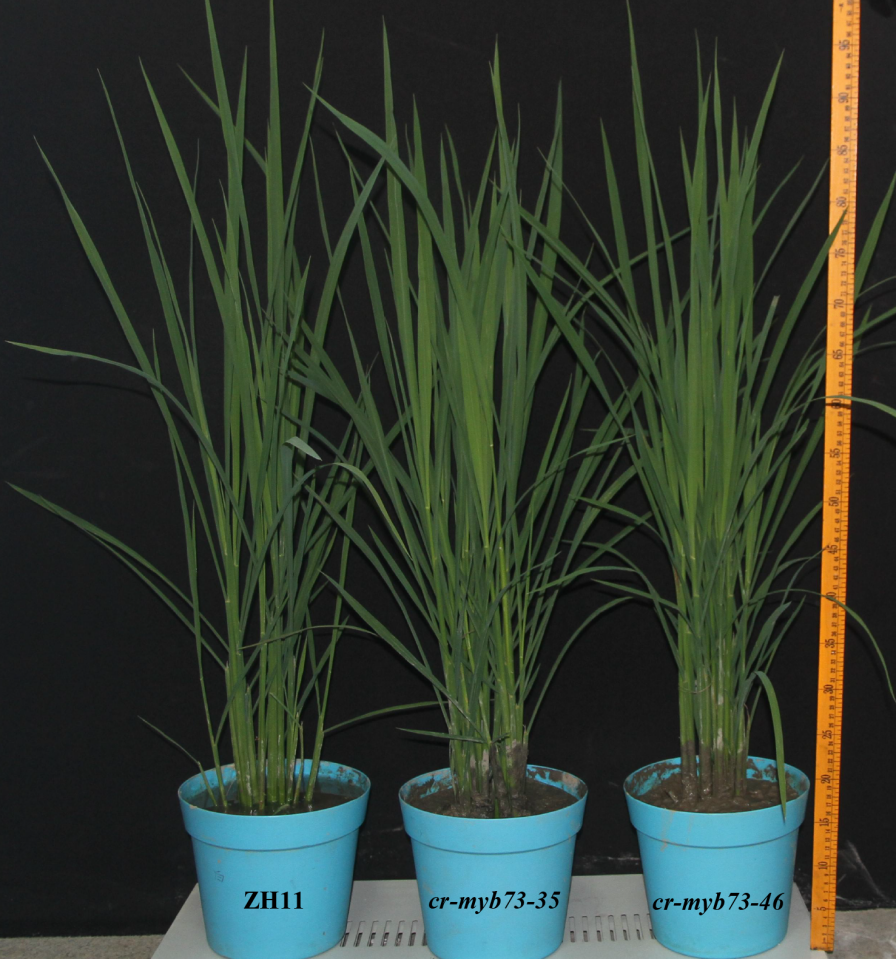


**Figure S4: Comparison of plant morphology between wild-type (ZH11) and mutants (*****cr-myb73-35*, *cr-myb73-46*).**

**
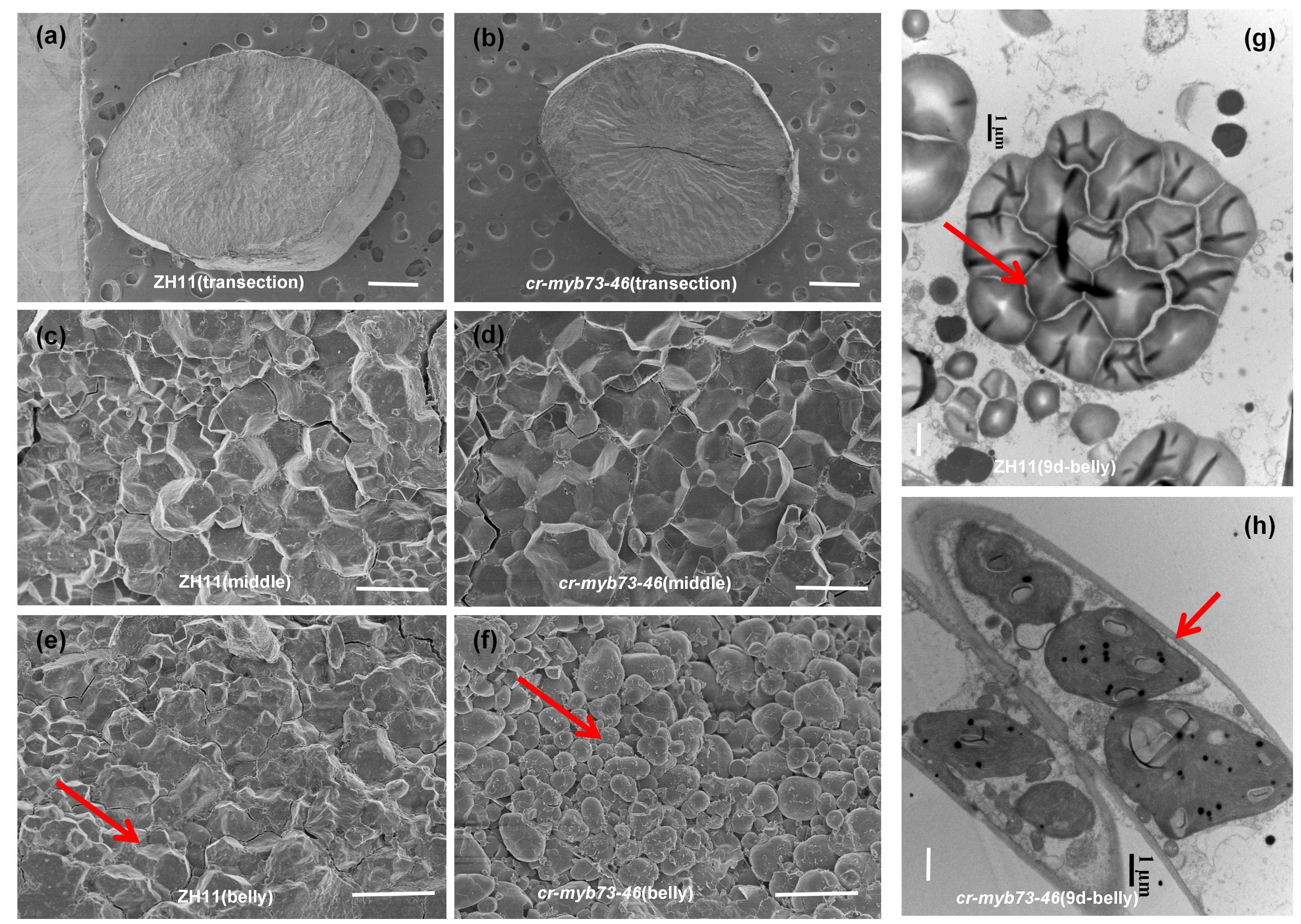
**

**Figure S5:** **Scanning and transmission electron microscopy images of wild-type (ZH11) and** **mutant (*cr-myb73-46*) in T_1_ generation****.**

(a) ZH11 transection picture; (b) *cr-myb73-46* transection picture; (c) Scanning electron microscopy (SEM) analysis of ZH11 starch granule in the middle of endosperm; (d) Scanning electron microscopy (SEM) analysis of *cr-myb73-46* starch granule in the middle of endosperm; (e) Scanning electron microscopy (SEM) analysis of ZH11 starch granule in the belly endosperm; (f) Scanning electron microscopy (SEM) analysis of *cr-myb73-46* starch granule in the belly endosperm; (g) ZH11 amyloplast structure of 9 days after fertilization; (h) *cr-myb73-46* amyloplast structure of 9 days after fertilization. Scale bars are 0.5 mm in transection pictures a, b, 5 μm in c-f , and 1 μm in g, h.


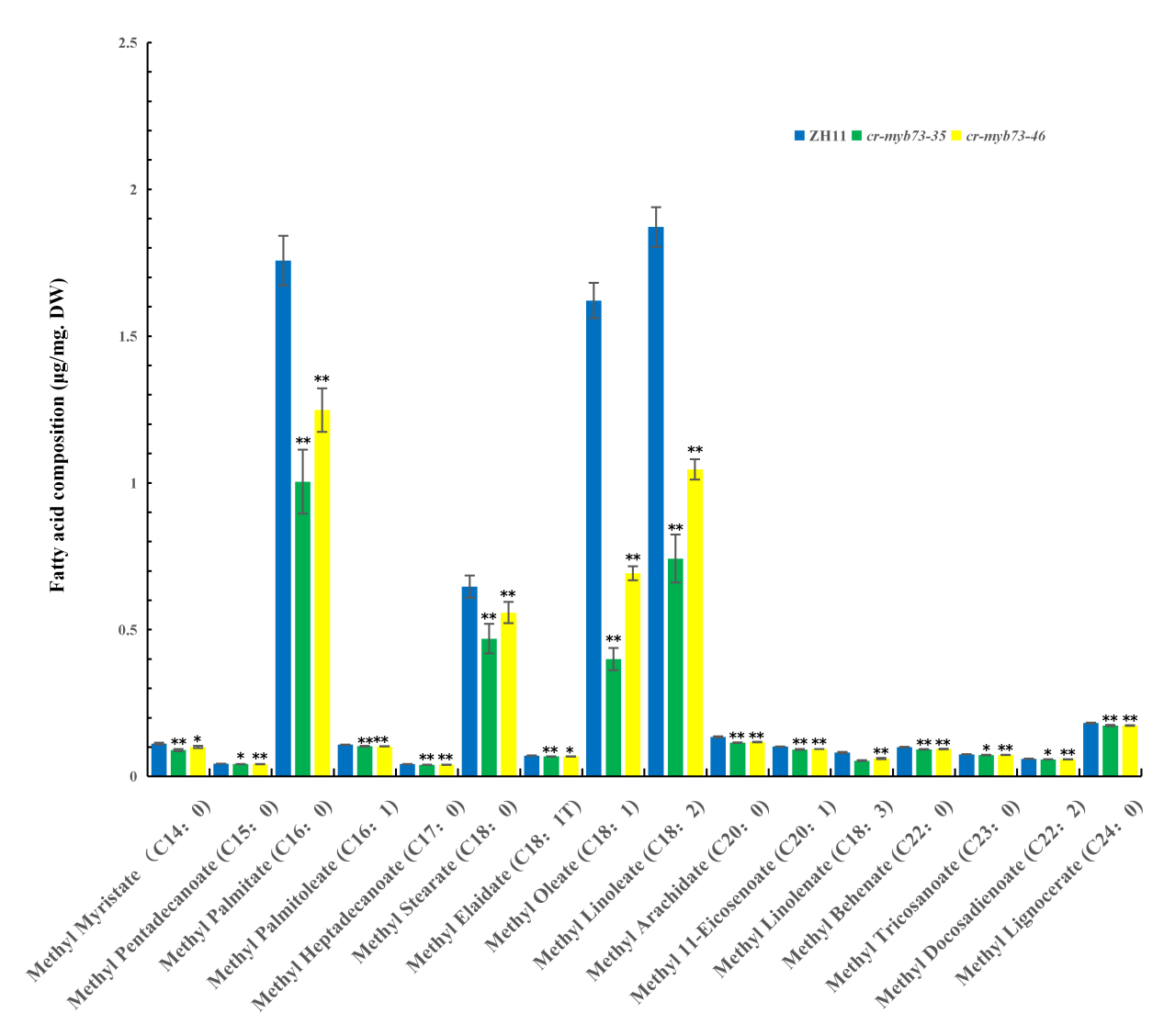


**Figure S6:** **Composition of medium and long chain fatty acid in mature grains between wild-type (ZH11) and mutants (*cr-myb73-35*, *cr-myb73-46*).**

Values are means ± SD form three biological replicates. Asterisks indicate statistical significance, as determined by a Student’s *t-*test (* *P* < 0.05, ** P < 0.01).


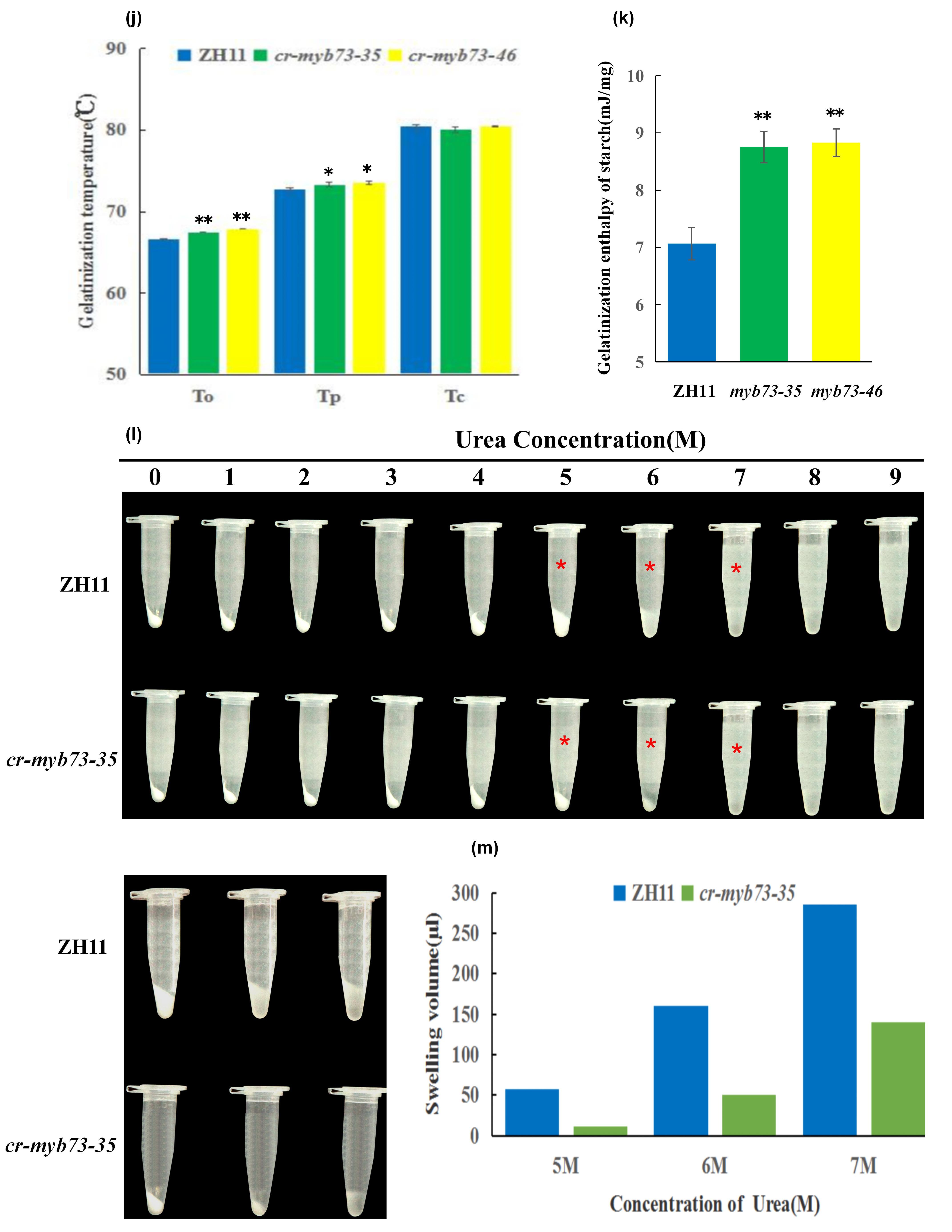


**Figure S7:** **Grain starch physicochemical characteristics comparison of wild-type (ZH11) and mutants (*cr-myb73-35*, *cr-myb73-46*) in T_1_ generation (Continued Figure 3).**

(j) Thermal characteristics presented by modulated differential scanning calorimetry (MDSC) curves. To, Onset temperature; Tp, Peak temperature; Tc, Conclusion temperature; (k) Gelatinization enthalpy of starch ΔH (mJ/mg); (l, m) Gelatinization characteristics of starch from *cr-myb73* mutant seeds. Starch powder was mixed with different concentrations (0-9 M) of urea solution. Asterisks indicate statistical significance, as determined by a Student’s *t-*test (* *P* < 0.05, ** *P* < 0.01).


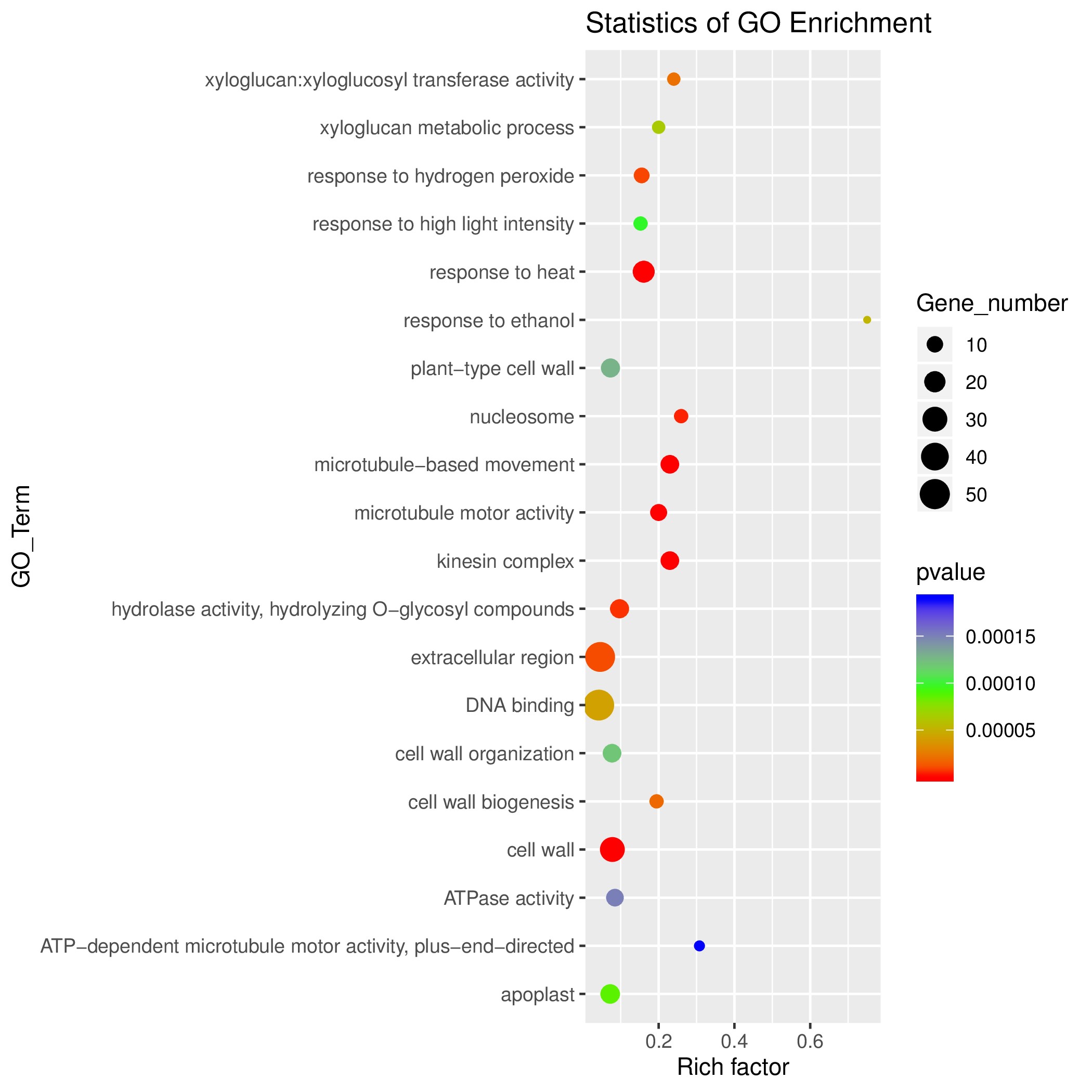


**Figure S8:** **RNA-sequencing transcriptomic analysis of ZH11 and *cr-myb73-46* (*cr-myb73-46* VS ZH11 GO enrichment scatterplot).**


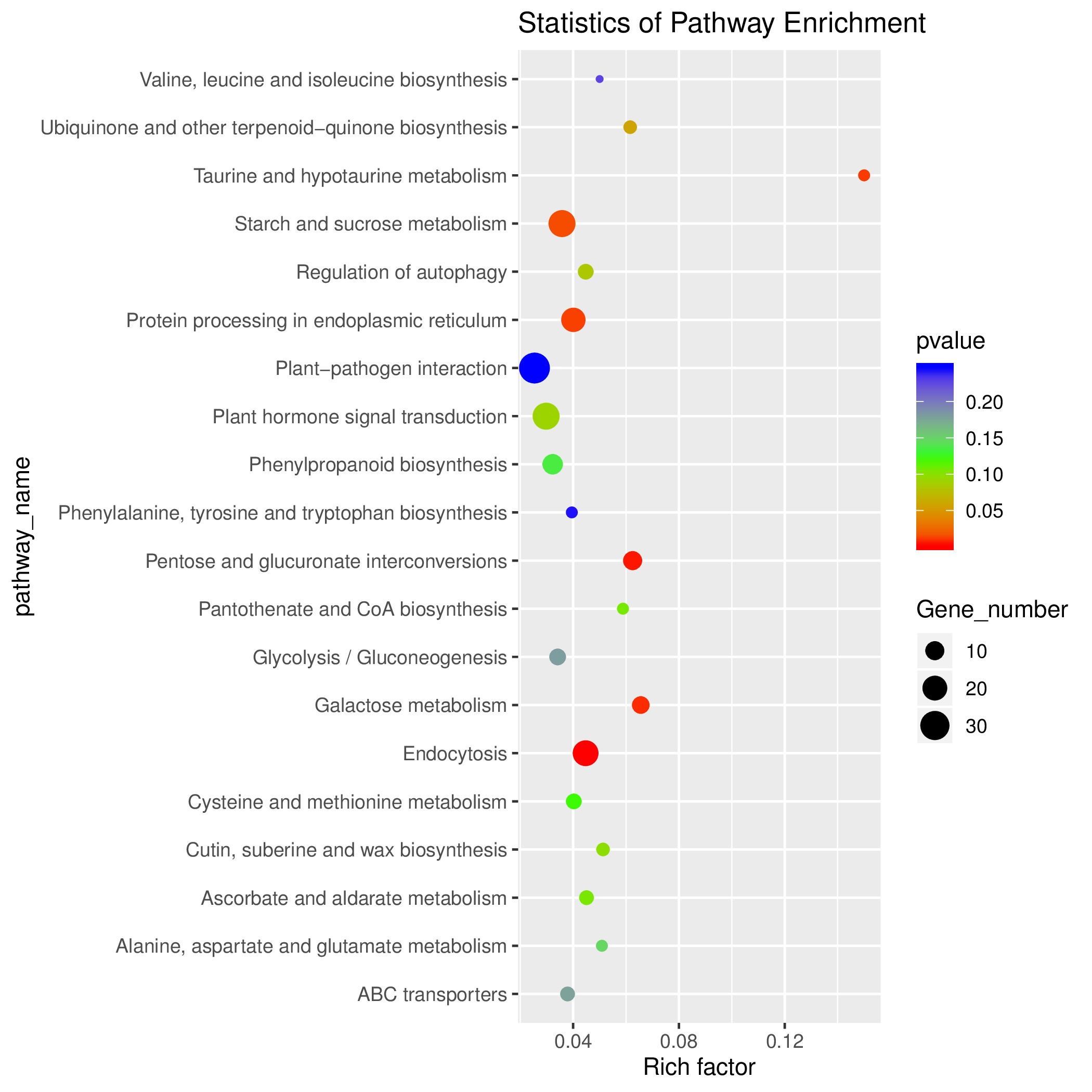


**Figure S9:** **RNA-sequencing transcriptomic analysis of ZH11 and *cr-myb73-46* (*cr-myb73-46* VS ZH11 KEGG enrichment scatterplot)*.***


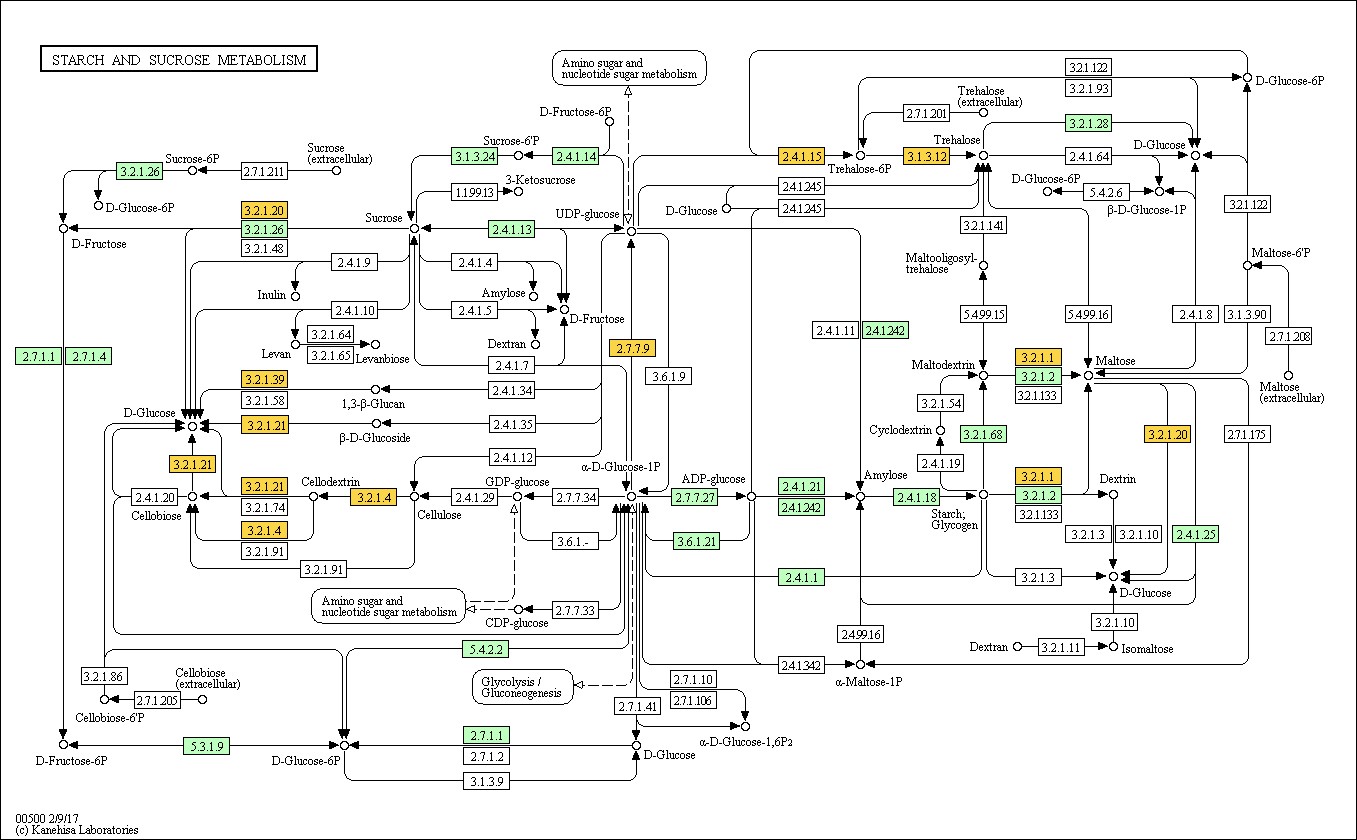


**Figure S10:** **RNA-sequencing transcriptomic analysis of ZH11 and *cr-myb73-46* (*cr-myb73-46* VS ZH11 starch and sucrose metabolic pathway).**

Pathway diagram: Red represents significantly differentially up-regulated expressed transcripts annotated to a ko node and; Blue represents significantly differentially down-regulated expressed transcripts annotated to a ko node and; Orange represents significantly differentially both up-regulated and down-regulated expressed transcripts annotated to a ko node. The four digit figure in the box indicate the EC number of each enzyme; Hollow circles indicate small molecule compounds; Solid arrows indicate the direction of biochemical reactions; The dashed arrows connect other related metabolic pathways.


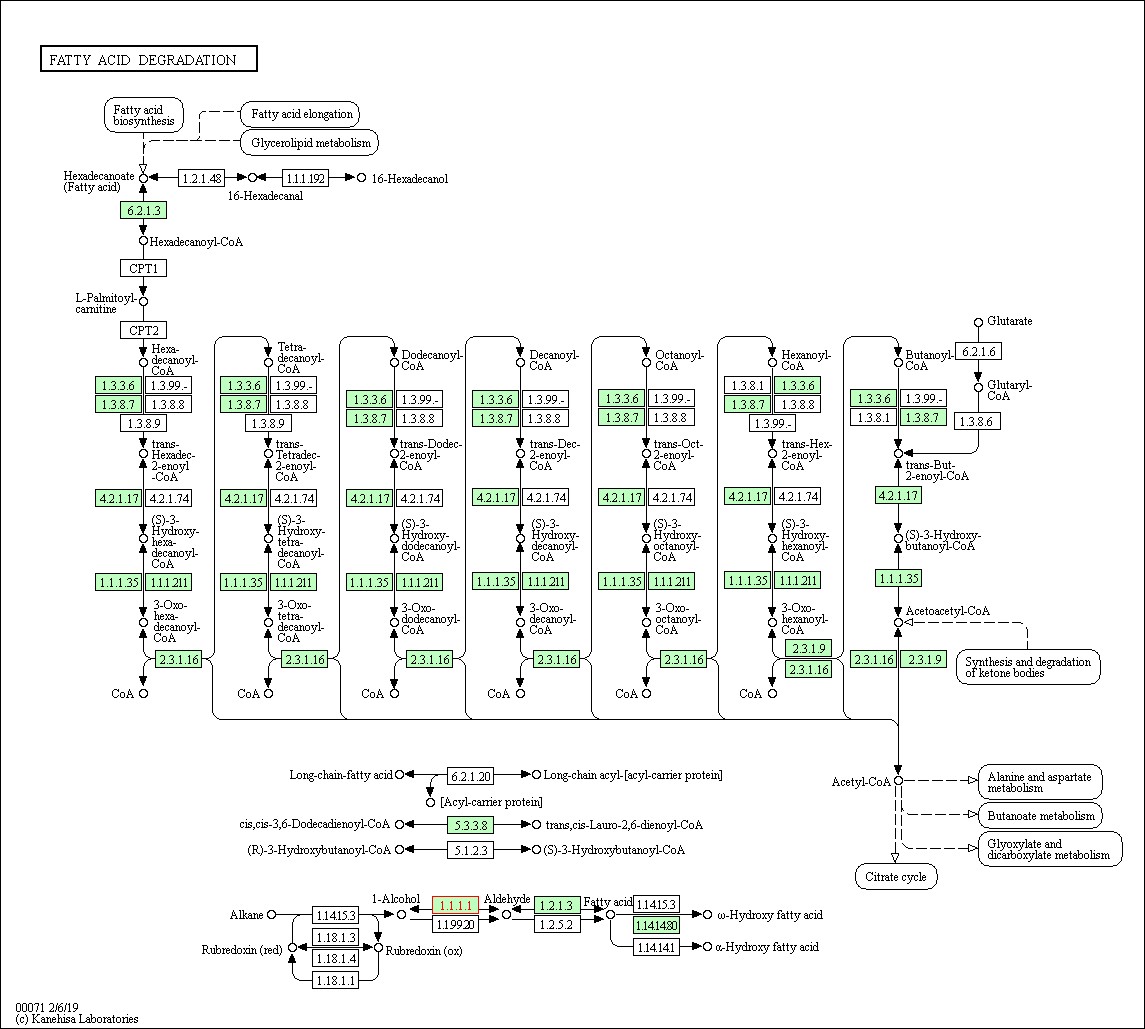
**Figure S11:** **RNA-sequencing transcriptomic analysis of ZH11 and *cr-myb73-46* (*cr-myb73-46* VS ZH11** **fatty acid degradation pathway).**

Pathway diagram: Red represents significantly differentially up-regulated expressed transcripts annotated to a ko node and; Blue represents significantly differentially down-regulated expressed transcripts annotated to a ko node and; Orange represents significantly differentially both up-regulated and down-regulated expressed transcripts annotated to a ko node. The four digit figure in the box indicate the EC number of each enzyme; Hollow circles indicate small molecule compounds; Solid arrows indicate the direction of biochemical reactions; The dashed arrows connect other related metabolic pathways.

**
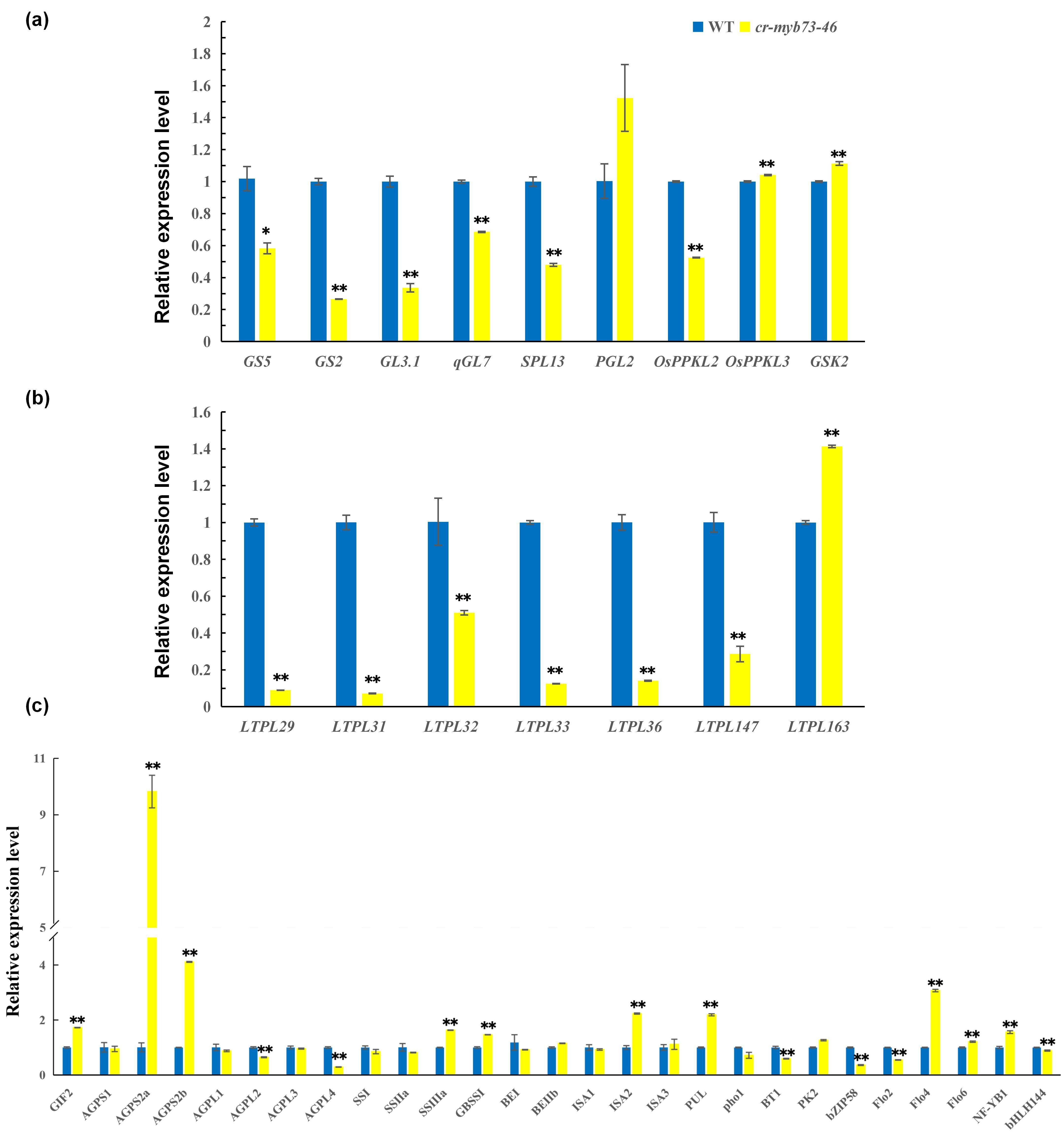
**

**Figure S12:** **The relative expression level of** **grain shape, lipid transport protein and** **endosperm starch synthesis related genes of wild-type (ZH11) and mutant (*cr-myb73-46*) at 5 DAF in the seeds.**

(a) Relative expression level of grain shape related genes of WT and *cr-myb73-46*; (b) Relative expression level of lipid transport protein related genes of WT and *cr-myb73-46*; (c) Relative expression level of starch biosynthesis related genes of WT and *cr-myb73-46*. Each value is the mean SD of at least three independent measurements with SEs, and significant differences were determined using Student’s *t-*test (* *P* < 0.05, ** *P* < 0.01).


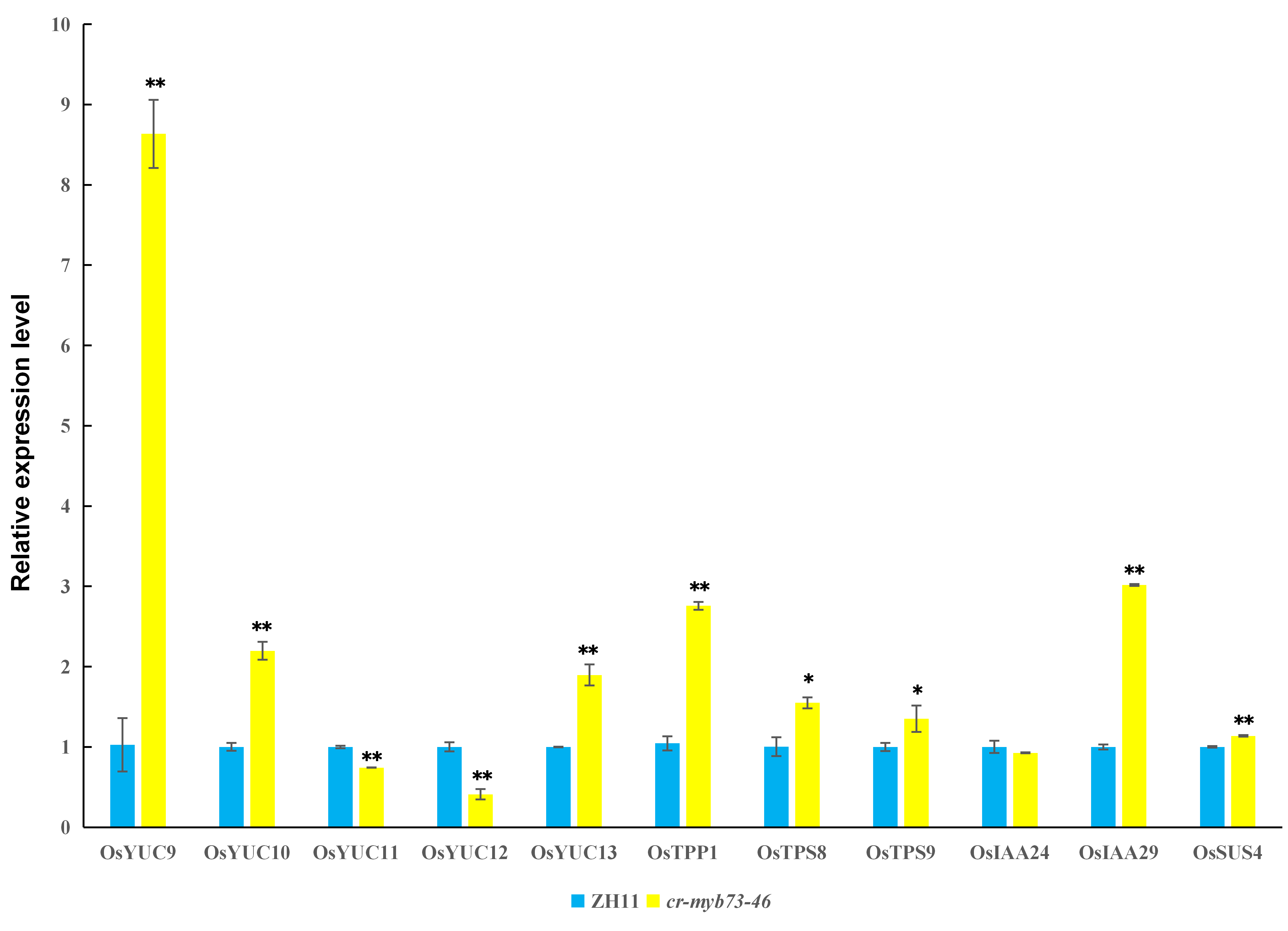


**Figure S13: The relative expression level of auxin biosynthesis, trehalose-6-phosphate synthase (*TPS*) and sucrose synthase (*SUS*) related genes of wild-type (ZH11) and mutant (*cr-myb73-46*) at 5 DAF in the seeds.**

Each value is the mean SD of at least three independent measurements with SEs, and significant differences were determined using Student’s t-test (* P < 0.05, ** P < 0.01).


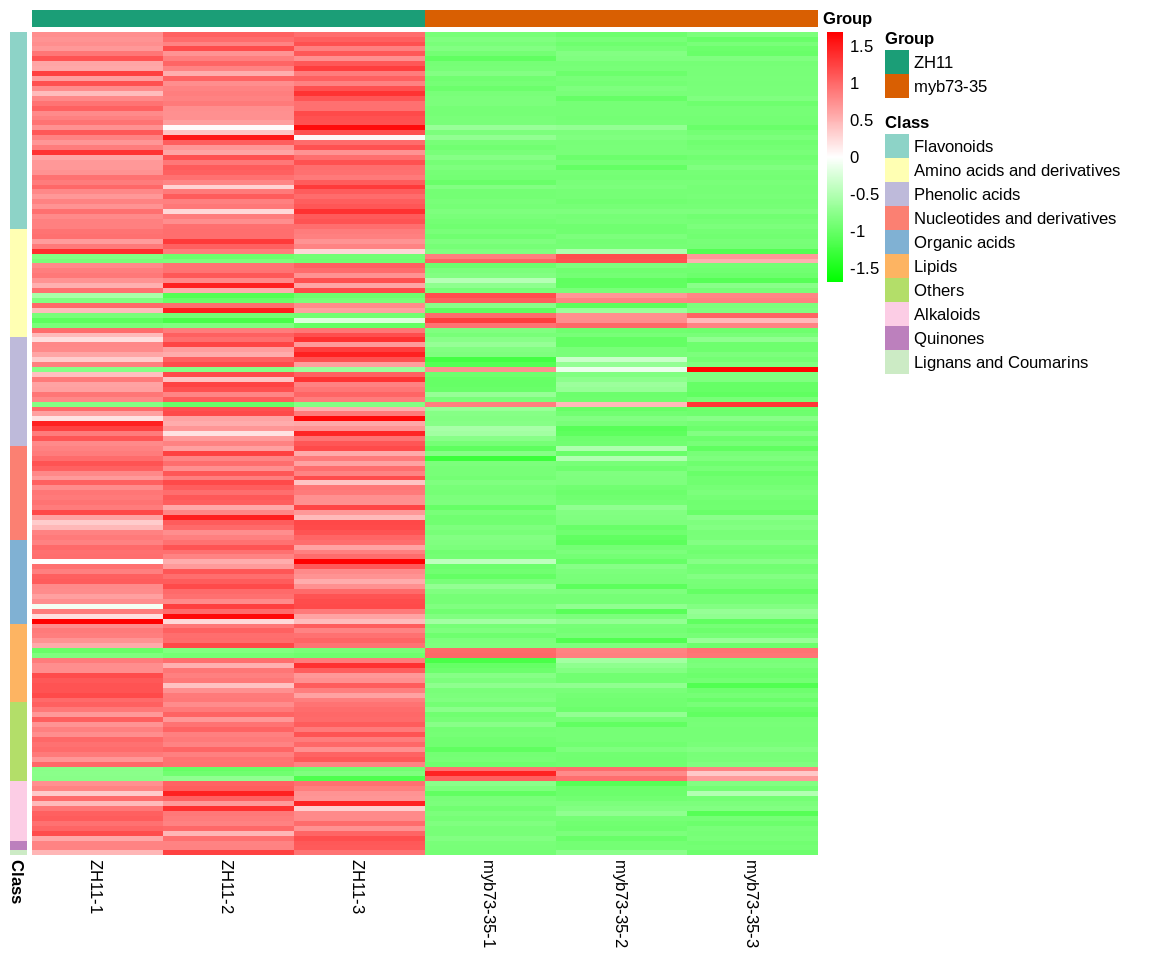


**Figure S14:** **Heat map of metabolites comparation in rice endosperm between wild-type (ZH11) and mutants (*myb73-35*). -1, -2 and -3 are three biological replicates of wild-type and mutants.**

Fold changes are indicated by varying shades of green (decrease) and red (increase) compared to wild-type seeds. Note: Horizontal refers to sample name, vertical refers to metabolite information, Group refers to grouping, Class refers to substance classification, and different colors refer to values obtained after standardized treatment of relative content (red represents high content, green represents low content).


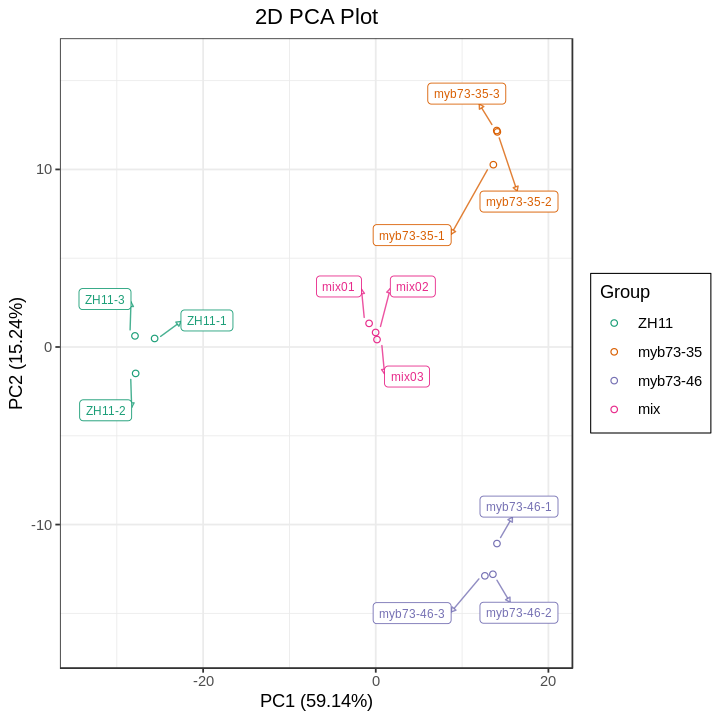


**Figure S15:** **Principal component analysis (PCA) of polar and non-polar metabolites identified in rice endosperm.**

The wild-type seeds (green circles) are compared to three biological replicates of mutants (*myb73-35*, *myb73-46*) seeds (yellow and purple circles). The score plots display the average of three technical replicates.


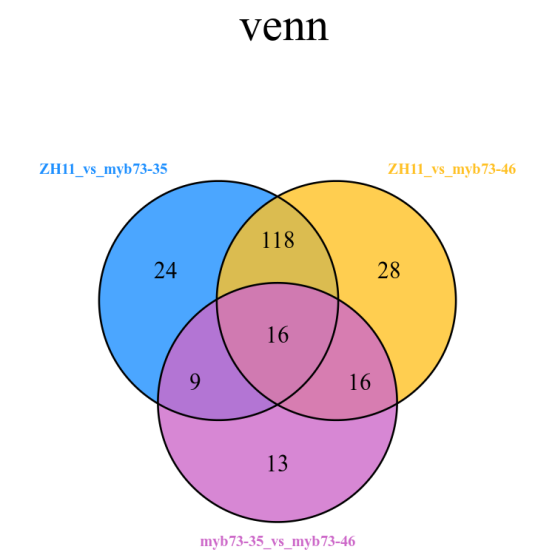


**Figure S16: Venn diagram showing** **differentially metabolites.**


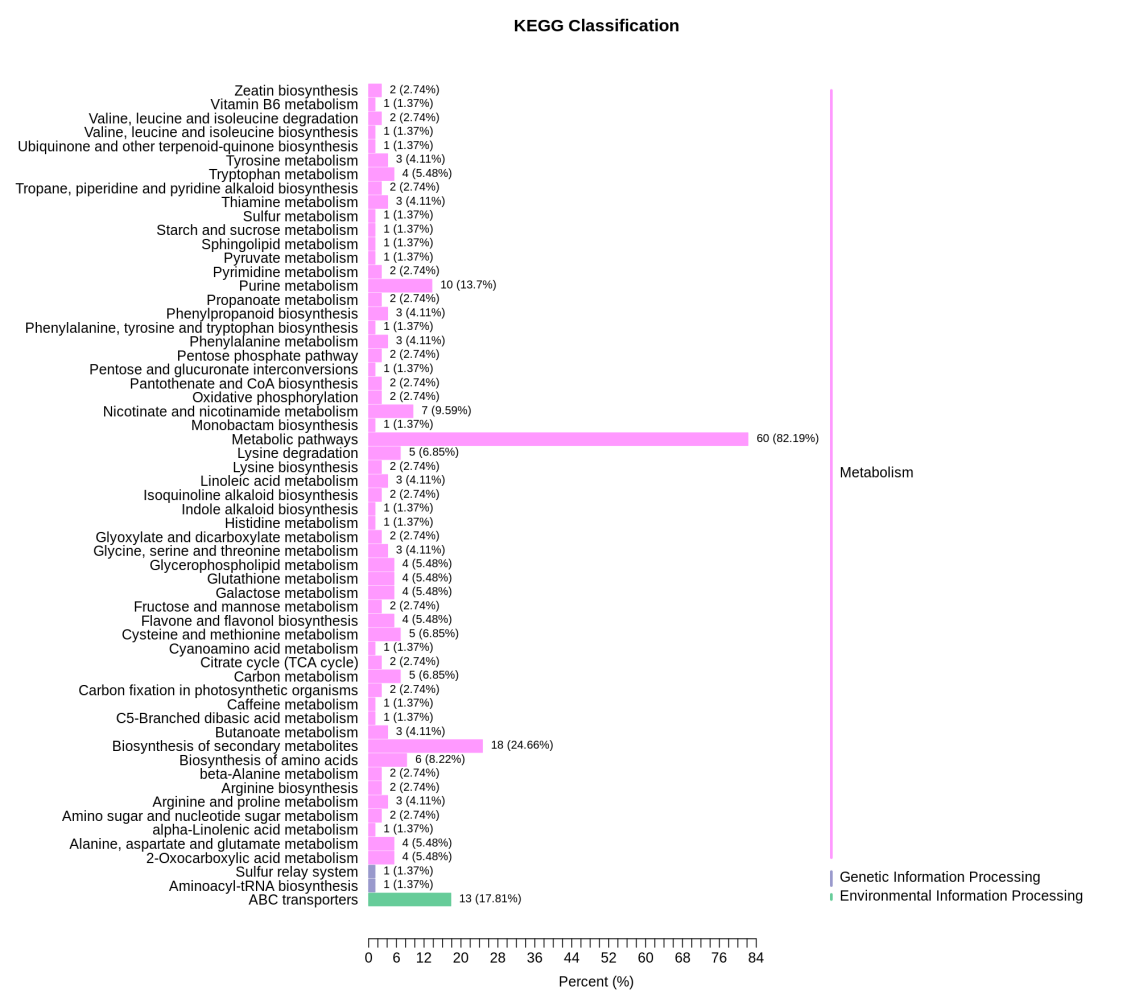


**Figure S17: ZH11_vs_*myb73-35* KEGG barplot** **of differentially metabolites classification.**


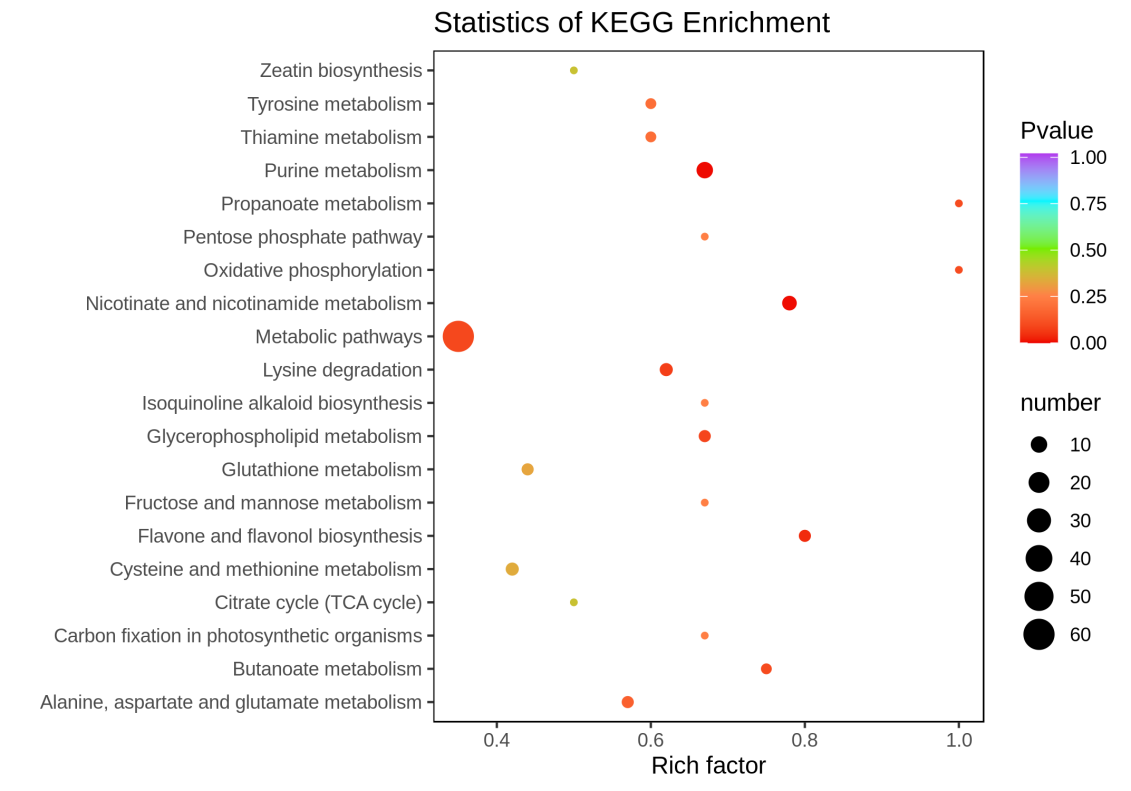


**Figure S18:** **ZH11_vs_*myb73-35* KEGG enrichment of differentially metabolites.**


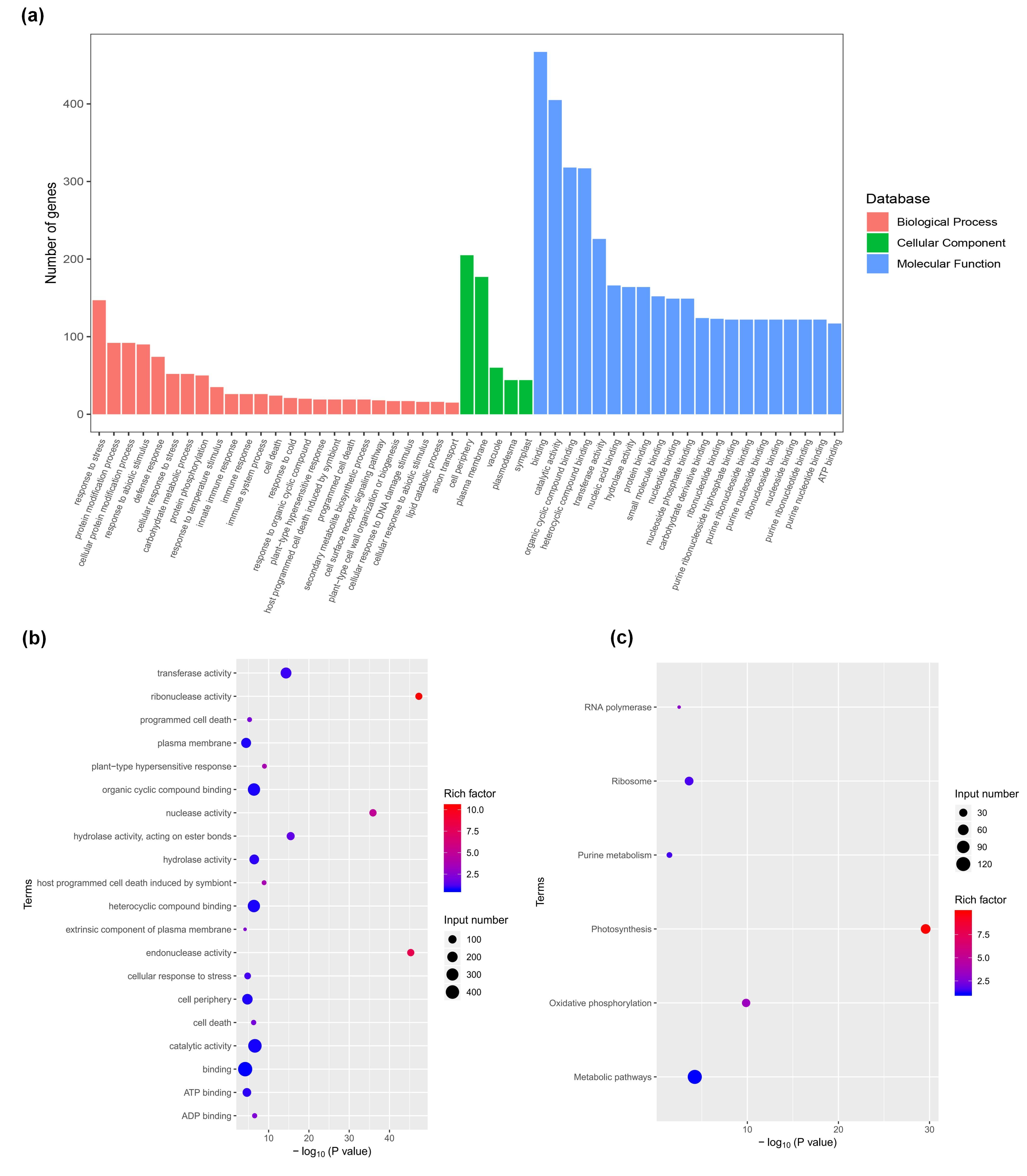


**Figure S19:** **Peak related genes annotation of GO and KEGG.**

(a) Peak related genes annotation of GO enrichment; (b) Peak related genes annotation of GO; (c) Peak related genes annotation of KEGG pathways.


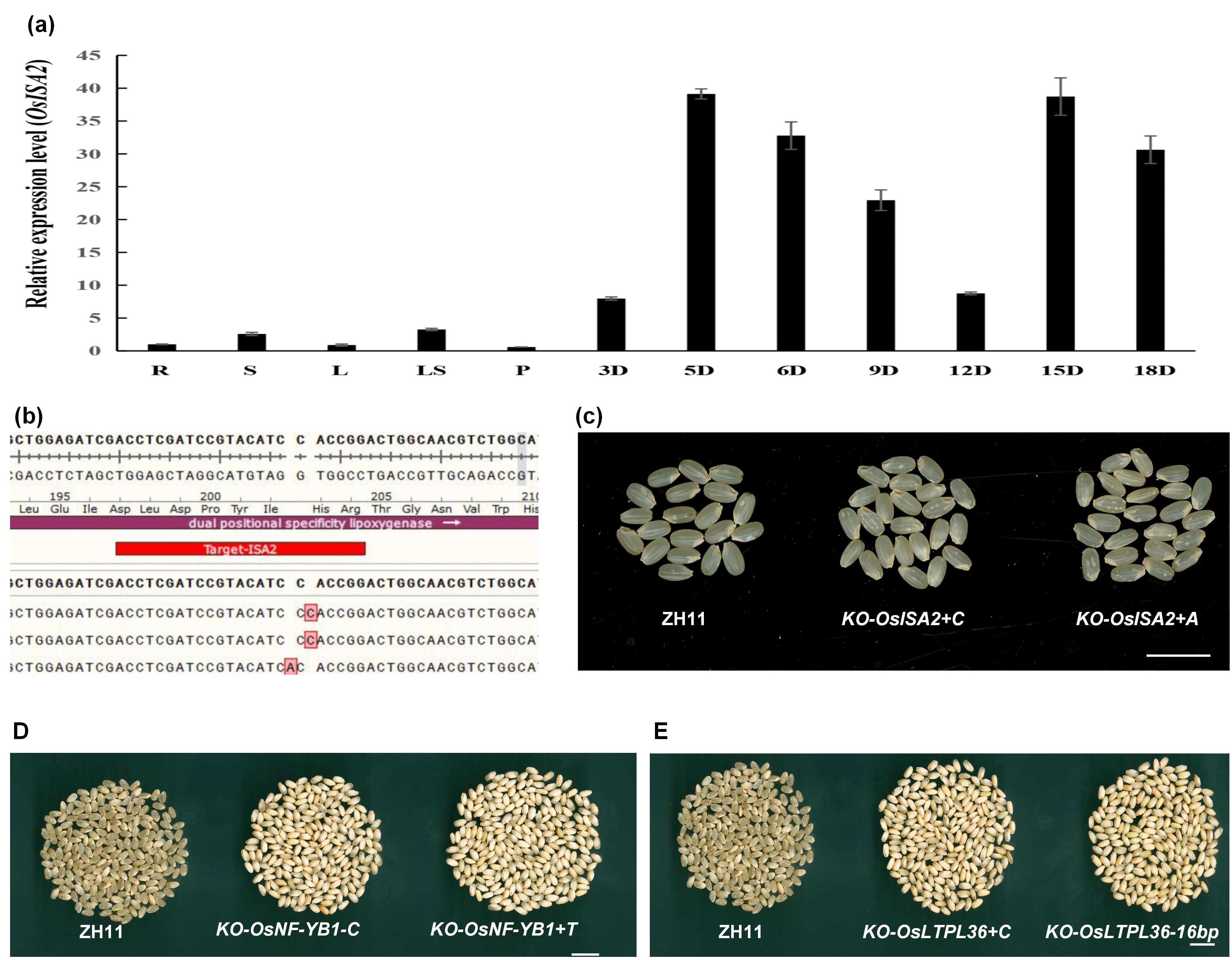


**Figure S20: Rice *OsISA2* spatiotemporal expression pattern and CRISPR/Cas9 mediated target mutagenesis of *OsISA2* mutant brown rice grains phenotypic evaluation in T1 generation.**

R, Root; S, Steam; L, Leaf; LS, Leaf sheath; P, Panicle; 3D, 5D, 6D, 9D, 12D, 15D, 18D means 3, 5, 6, 9, 12, 15, 18 days after fertilization. Bar, 1.0 cm.


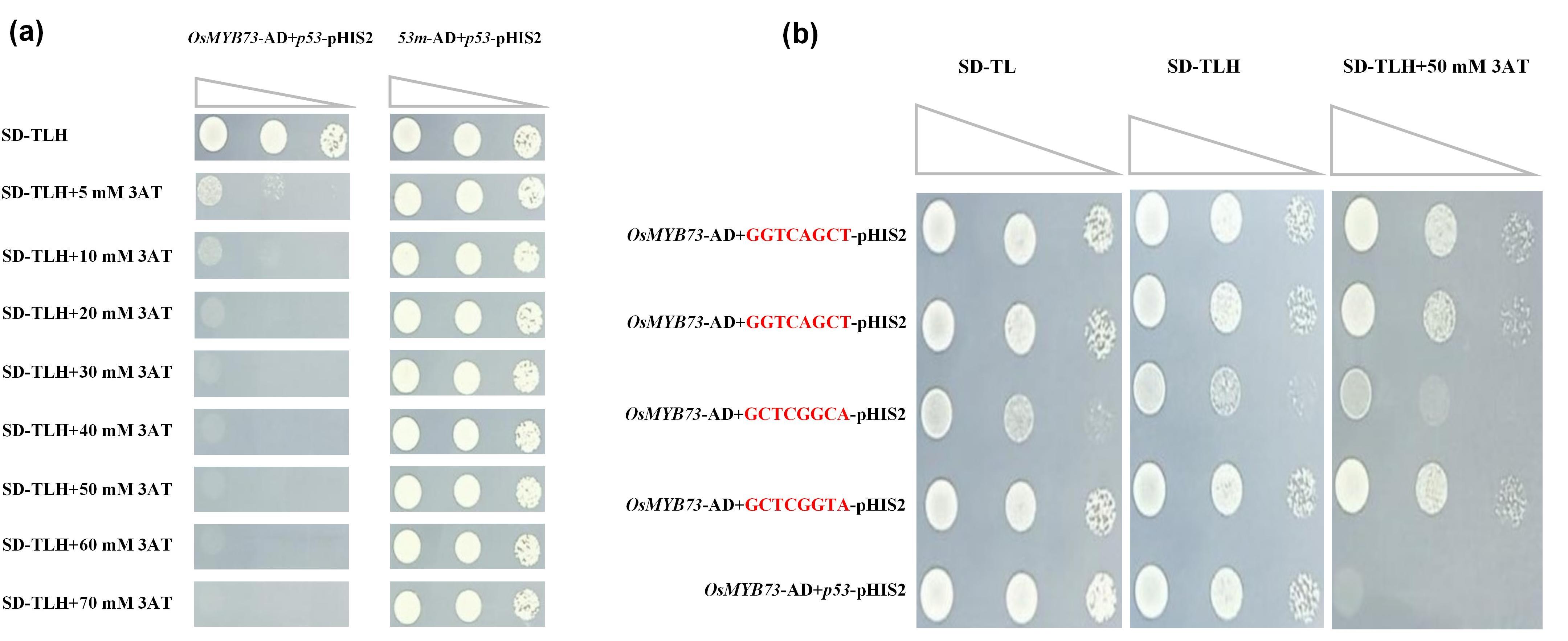


**Figure S21:** **Screening binding motifs of OsMYB73 (TF-centered Y1H).**

(a) 3-AT concentration screening; (b) Binding motifs screening.

**
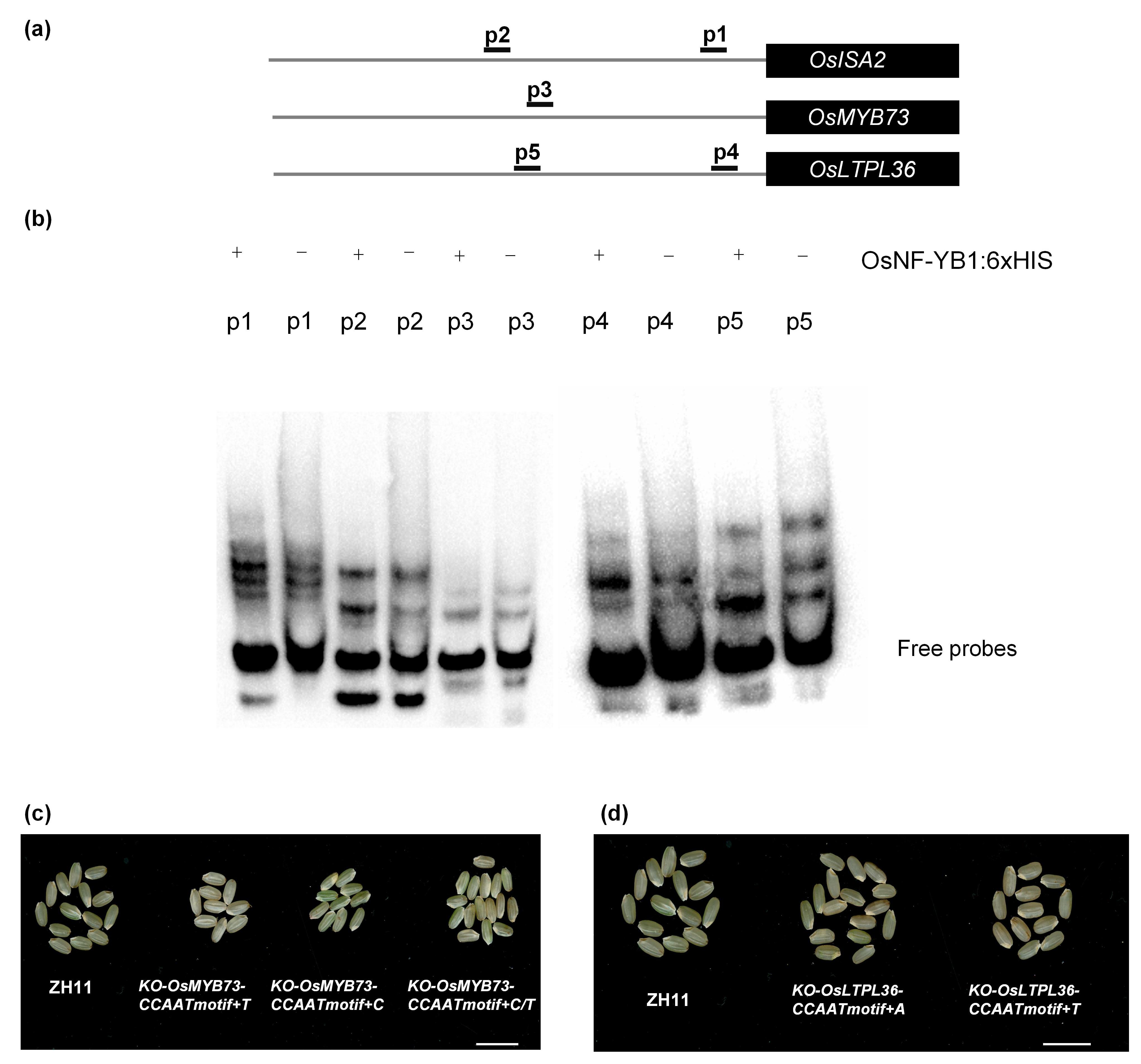
**

**Figure S22: In vitro analyses of binding and activation ability of OsNF-YB1 to *OsMYB73*, *OsISA2* and *OsLTPL36,* and knock out *OsMYB73* and *OsLTPL36* promoters CCAAT-motif brown rice phenotypic evaluation.**

(a). Illustration of the probes that contain putative CCAAT motifs and were used for electrophoretic mobility shift assay (EMSA); (b). The EMSA assay failed to detect binding ability of OsNF-YB1 to the *OsMYB73*, *OsISA2* and *OsLTPL36* promoters, we showed the result for probe 1, probe 2, probe 3, probe 4 and probe 5 (p1, p2, p3, p4, p5). (c). *OsMYB73* promoter CCAAT-motif brown rice phenotypic evaluation; (d). *OsLTPL36* promoter CCAAT-motif brown rice phenotypic evaluation. Bar, 1.0 cm.
