# Supplementary material for: A novel transcription factor OsMYB73 affects grain size and chalkiness by regulating endosperm storage substances' accumulation‐mediated auxin biosynthesis signalling pathway in rice
Source: Plant Biotechnol J. 2024 Dec 26;23(4):1021–38. doi: 10.1111/pbi.14558 (PMC11933829; doi:10.1111/pbi.14558)
Supplement: Supplementary file 2 — Table S1 The primers used in this study. [file PBI-23-1021-s007.docx]

**Supplementary Table 1.** The primers used in this study.

| Primer name | Forward (5′–3′) | Reverse (5′–3′) |
| --- | --- | --- |
| Target | GCCATGGCTACCGTTCCAGT | ACTGGAACGGTAGCCATGGC |
| Seq | AGAACAAGTTGATGGCGGCTGCA | ATCTTCTTGCCTTGTCCATGCTCCT |
| Hyg | GCTGTTATGCGGCCATTGTC | GACGTCTGTCGAGAAGTTTC |
| Sq-primer（SQ） | CTCCTTCCTTCCGTCCACTTCATC |  |
| Histochemical GUS | CCATGATTACGAATTCTTGCATGCGGGGTCAGTTAT | CTCAGATCTACCATGGTGCATCAGCCGTTCATCTGT |
| PAN580 | GCCCAGATCAACTAGTATGCTTCCAACCTCAAAGAA | TCGAGACGTCTCTAGACTTGAACCTGGTCAGGTCGA |
| Overexpression | TTACTTCTGCACTAGGTACC  ATGCTTCCAACCTCAAAGAA | TGGCTAGCGTTAACACTAGT TCACTTGAACCTGGTCAGGT |
| PGADT7  +OsMYB73 | GGAGGCCAGTGAATTCATGCTTCCAACCTCAAAGA | CGAGCTCGATGGATCCTCACTTGAACCTGGTCAGG |
| PGADT7  +OsNF-YB1 | GGAGGCCAGTGAATTCATGGCAGGGAACAAAAAGC | CGAGCTCGATGGATCCTCACATATTTTTTCCATAG |
| PGADT7  +OsLTPL36 | GGAGGCCAGTGAATTCATGGCGAGACAACAACTCC | CGAGCTCGATGGATCCTTATTGCCCTAGGCTCGAT |
| PGBT9  +OsMYB73 | TGTATCGCCGGAATTCATGCTTCCAACCTCAAAGA | GCAGGTCGACGGATCCTCACTTGAACCTGGTCAGG |
| PB42AD | TGCCTCTCCCGAATTCATGCTTCCAACCTCAAAGA | CGAGTCGGCCGAATTCTCACTTGAACCTGGTCAGG |
| PLaczi  +OsISA2 | TGCCTCTCCCGAATTCTTTGTTCCTCTTACTATTC | CGAGTCGGCCGAATTCGCGTGCGTGCGTGACGTTC |
| PLaczi  +OsLTPL36 | ATCTGTCGACCTCGAGGAGCCCAAGTAGTCTTCCG | GAGCACATGCCTCGAGAGTTGGTAGGCAGTTTG |
| OsMYB73  +nYFP | ATCGAGGACGCCGGCGGATCATGCTTCCAACCTCAAAGAACAAGTTGATGG | GCTCTGCAGGTCGACTCTAGTCACTTGAACCTGGTCAGGTCGAG |
| OsNF-YB1  +cYFP | ATTACAGGTACCCGGGGATCATGGCAGGGAACAAAAAGCGTGGTG | GCCACCGCCGTCGACTCTAGCATATTTTTTCCATAGCCATATCCATAGCCATAGC |
| OsMYB73  +nLuc | ACGGGGGACGAGCTCGGTACATGCTTCCAACCTCAAAGAACAAGTTGATGG | CGCGTACGAGATCTGGTCGACTTGAACCTGGTCAGGTCGAGAG |
| OsNF-YB1  +cLuc | TACGCGTCCCGGGGCGGTACATGGCAGGGAACAAAAAGCGTGGTG | ACGAAAGCTCTGCAGGTCGATCACATATTTTTTCCATAGCCATATCCATAGCCATAG |
| Ubiquitin | GCTCCGTGGCGGTATCAT | CGGCAGTTGACAGCCCTAG |
| OsMYB73 | CTACAACGATCAGCGAGAGG | ACATGCATCCCATAGTGCAG |
| GS5 | AGTGGACTGCTTCCAGGGAAG | CACGCAGTACCGAGAACTGA |
| GS2 | ATTCCAAGTACTGCGAGCGC | GGCGACCAGCTGCGTTT |
| GL3.1 | ACAACTCCCAGGATAGGAAGA | CCTCCTCGTAGACCTCCATAAA |
| qGL7 | GGGACACCGGAGGCCTTA | TGCCCATTCCTCCTTGCAT |
| SPL13 | AACCCGCCGTTCCAGATCAG | AAGAAGGGACGTAGGTGGTG |
| PGL2 | ATGTCGAGCAGAAGGTCGTC | TCAGGAGCGGAGGATGCTGC |
| OsPPKL2 | TACTTTGCGGTGGAAGGGAT | CAGTAACATGCAACCGAGCA |
| OsPPKL3 | CAAACAATGCGGGTGCAATC | TTCCTGCATCCATGTGTCCT |
| GSK2 | CTCCTTGGTCAGCCATTGTT | GCGAGGTCTATTGCTTCAGG |
| LTPL29 | ACGCATGCTGTAGCGTAATC | CGTGTAGCTTCCACACTTGG |
| LTPL31 | ATTGCTGCCAAACAGTGGAG | CGTGTAACGGATTGCCACAT |
| LTPL32 | TGCGAGAAGTTCATCAGGGT | TCACCCTCTGCTTGTCCAAT |
| LTPL33 | ATTCTGCATGTTGCGATGCT | GATCCTGTTGGCGTTGTACC |
| LTPL36 | AAGGTCGTCTATGTCGCCAA | TATTGCCCTAGGCTCGATGG |
| LTPL147 | GACCTGATAAAGGCGAAGG | GTTGAGGATGAGGCTGAGGT |
| LTPL163 | GGTGCAACCTGCAGCACA | AATCCATGCCACCACCAAAC |
| GIF2 | AAGAGGTGCTTTGGTGATG | AAGAGGTGCTTTGGTGATG |
| OsAGPS1 | GTGCCACTTAAAGGCACCATT | CCCACATTTCAGACACGGTTT |
| OsAGPS2a | ACTCCAAGAGCTCGCAGACC | GCCTGTAGTTGGCACCCAGA |
| OsAGPS2b | AACAATCGAAGCGCGAGAAA | GCCTGTAGTTGGCACCCAGA |
| OsAGPL1 | GGAAGACGGATGATCGAGAAAG | CACATGAGATGCACCAACGA |
| OsAGPL2 | TACACAAATGCCTGACGAAC | CTCCACACAAGATTACAACG |
| OsAGPL3 | AAGCCAGCCATGACCATTTG | CACACGGTAGATTCACGAGACAA |
| OsAGPL4 | TCAACGTCGATGCAGCAAAT | ATCCCTCAGTTCCTAGCCTCATT |
| OsSSI | GGGCCTTCATGGATCAACC | CCGCTTCAAGCATCCTCATC |
| OsSSIIa | GCTTCCGGTTTGTGTGTTCA | CTTAATACTCCCTCAACTCCACCAT |
| OsSSIIIa | GGCCAAGTACCAATGGTAAA | GCATGATGCATCTGAAACAAAGC |
| OsGBSSI | AACGTGGCTGCTCCTTGAA | TTGGCAATAAGCCACACACA |
| OsBEI | TGGCCATGGAAGAGTTGGC | CAGAAGCAACTGCTCCACC |
| OsBEIIb | ATGCTAGAGTTTGACCGC | AGTGTGATGGATCCTGCC |
| OsIAS1 | TGCTCAGCTACTCCTCCATCATC | AGGACCGCACAACTTCAACATA |
| OsIAS2 | TAGAGGTCCTCTTGGAGG | AATCAGCTTCTGAGTCACCG |
| OsIAS3 | ACAGCTTGAGACACTGGGTTGAG | GCATCAAGAGGACAACCATCTG |
| OsPUL | ACCTTTCTTCCATGCTGG | CAAAGGTCTGAAAGATGGG |
| pho1 | TTGGCAGGAAGGTTTCGCT | TTGGCAGGAAGGTTTCGCT |
| OsBT1 | GGCATCTCCTTCATGTGCTA | CGCAACCTTCTCCTTGATCT |
| OsPK2 | CATGAGGAACTGGAACTTAAA | GACGAAAACGGCATCTAC |
| bZIP58 | GACCTGGAGGAGCAGGTATC | TAGGGCTTCAATGTCTGCCA |
| Flo2 | CAAGAGTCCGTCAGGTCG | GTGCTGCTTGCTTCTTCTAC |
| Flo4 | CTTGGTTTCCGTGGGTGC | AGCCTGCTCTGCTATCTCAT |
| Flo6 | GCTTTAATGGGTCAGAGTGG | AATGATTTCCTGCTGTTAGTTG |
| NF-YB1 | ATGGTTACCGTGAGTTTGAGAG | CAGCTCTGCGTCAGTGAAT |
| bHLH144 | AACATGGCCGTCAAGAGAGA | CAAATATGCAACGCGTGCTC |
| OsYUC9 | CCTGGTGAGCAAGGAGATATG | AGCCATTGTTGTGGGTAGG |
| OsYUC10 | AGGTTGTGCTGCTCATGT | CGGCGTGGTCATCTTCAT |
| OsYUC11 | GTCGGTCAGCAGTAATAGA | CTTGTGTAGCCTGTAGCA |
| OsYUC12 | GACAGCTACACTGACCGATTT | CACAGCCCAATGCTTCTTTC |
| OsYUC13 | AGAGCCTATACCGGGAAGAA | ATCGGGCTACGAACAACTATG |
| OsTPP1 | GGAGACGACAGAACTGACGAAGAC | CTCTCAGCGAGTAGAAGGCTTCAG |
| OsTPS8 | ACGGCTACTTCACAAGGTGG | ATTACAGGCCCTGCGATGTT |
| OsTPS9 | AGCTTTGCTCCGACCCTAAC | CGTCCCTGCTCCACCTAATG |
| OsIAA24 | TGTGCCCTGGGAAATGTTCA | GTGGTGTGTCCTAGCCTGTG |
| OsIAA29 | ATGGTGCAATAGACAGCGACT | CCATGGGACATCACCAAGGA |
| OsSUS4 | AAGTTCCGTGAACTGGCGAA | ACCATTCCCACTGACAGCAG |
| P1 | CTGACCGACTCCTCTTCACCAATGACTTCCCCACGGCGGT | ACCGCCGTGGGGAAGTCATTGGTGAAGAGGAGTCGGTCAG |
| P2 | CGTGCAGGCTCGAGCGGCACATTGGGCGGACGATAAATCT | AGATTTATCGTCCGCCCAATGTGCCGCTCGAGCCTGCACG |
| P3 | AGGACCAATTAGCTCCATTGGTCATTCTTCATCCAATGGC | GCCATTGGATGAAGAATGACCAATGGAGCTAATTGGTCCT |
| P4 | GGAGATCGGTAGTGGCCACTAATTGGGAAGGGGAGGGAGC | GCTCCCTCCCCTTCCCAATTAGTGGCCACTACCGATCTCC |
| P5 | GGTACTCCCTCTGTCCCAGATTGGAGGGCGAGTATTTTTT | AAAAAATACTCGCCCTCCAATCTGGGACAGAGGGAGTACC |
| Cas9-OsMYB73-CCAAT-motif | CAGTGAGGACCAATTAGCTCCAT | AACATGGAGCTAATTGGTCCTCA |
| Cas9-OsLTPL36-CCAAT-motif | CAGGTAGTGGCCACTAATTGGGA | AACTCCCAATTAGTGGCCACTAC |
