# Supplementary material for: A novel transcription factor OsMYB73 affects grain size and chalkiness by regulating endosperm storage substances' accumulation‐mediated auxin biosynthesis signalling pathway in rice
Source: Plant Biotechnol J. 2024 Dec 26;23(4):1021–38. doi: 10.1111/pbi.14558 (PMC11933829; doi:10.1111/pbi.14558)
Supplement: Supplementary file 8 — Appendix S1 The detailed steps of the methods involved in this study. [file PBI-23-1021-s004.docx]

**Appendix Information (The detailed steps of the methods involved in this study) for**

**A novel transcription factor *OsMYB73* affects grain size and chalkiness by regulating endosperm** **storage substances accumulation mediated auxin biosynthesis signaling pathway in rice**

Song Liu^a,b,1^, Jiamin Wu^a^^,1^, Amos Musyoki Mawia^a,1^, Xiangjin Wei^a,1^, Ruijie Cao^a,1^, Guiai Jiao^a^, Yawen Wu^a^, Jian Zhang^a^, Lihong Xie^a^, Zhonghua Sheng^a^, Shikai Hu^a^, Sanfeng Li^a^, Yusong Lv^a^, Feifei Lu^a^, Yujuan Chen^a^, Sajid Fiaz^a^, Javaria Tabassum^a^, Zhimin Du^a^, Fangyuan Gao^b,^*, Guangjun Ren^b,^*, Gaoneng Shao^a,^*, Peisong Hu^a,^*, Shaoqing Tang^a,^*

**Supplementary material for online publication only:** **The detailed steps of the methods involved in this study.**

**Materials and Methods**

**Plant materials and growth conditions**

T_0_ and T_1_ generations of *cr-myb73* transgenic lines generated using the CRISPR/Cas9 system and wild-type (WT) were grown under natural conditions in the fields of China National Rice Research Institute, Fuyang, Hangzhou, China, respectively. Homozygotes with different mutation types were detected in the T_0_ generation plants, and then planted the stable genetic homozygous for T_1_ generation on May 2019, and some relevant experiments were carried out. ZH11 was selected as the genetic background due to its easy to transform, moderate growth period, seldom chalkiness and high seed-setting rate. Initial sowing was carried out on May, and seedlings were transplanted into the paddy field on June. Fertilizer and water managements were used as standard field production. At least ten mature plants from wild-type and mutants were used to record the agronomic traits, and the seeds were used to examine the grain shape and weight. All experiments consisted of three biological replicates.

**RNA extraction and quantitative real-time PCR (qRT-PCR) analysis**

To investigate the expression of genes associated to grain shape, starch and lipid biosynthesis, total RNA was extracted from 5 DAF seeds (due to most of these genes have a relatively stable and high expression levels at this stage) by using the Trizol reagent (Invitrogen, https://www.thermofisher.com). First-strand cDNA was synthesized with 2 μg total RNA using the ReverTra Ace qPCR RT Kit (Toyobo, http://www.bio-toyobo.cn) according to the manufacturerʼs protocol. qRT-PCR was performed using a Light Cycler 480 device (Roche, <http://www.roche-applied-science.com)>with the SYBR Green Real-time PCR Master Mix (Toyobo, http://www.bio-toyobo.cn) in 20 µL reaction volume. The qRT-PCR conditions were as follows: 95 ºC for 30 s, 40 cycles of 95 ºC for 5 s, 60 ºC for 35 s and 95 ºC for 15 s. Assays included three biological replications. The gene-specific primers related to starch synthesis were those described by She et al (She *et al.*, 2010). The *Ubiquitin* gene (*Os03g0234200*) was used as an internal control, and the relative expression level was calculated by the 2^-ΔΔCT^ method. Primer pairs for RT-PCR amplification are shown in Supplemental Table S1*.*

**Histochemical GUS staining**

The putative promoter region of *OsMYB73* (~2 kb upstream of ATG) was amplified by PCR and cloned into the *EcoR*I/*Nco*I sites of pCAMBIA1305. The resultant construct was transformed into ZH11 calli and independent lines of positive T_0_ transgenic progeny were used to detect histochemical GUS activity. Tissues were submerged in histochemical GUS staining solution (10 mM EDTA, 0.1% Triton X-100, 1 mM 5-bromo-4-chloro-3-indoyl-b-D-glucuronide, 100 mM sodium phosphate (pH 7.0), 2.5 mM K_4_Fe(CN)_6_ and 2.5 mM K_3_Fe(CN)_6_ at 37 °C for 12-15 h. After incubation, tissues were discolored several times in pure ethyl alcohol.

**Subcellular localization**

The *OsMYB73* ORF without a termination codon was constructed into the PAN580-GFP vector, placing the *OsMYB73* gene upstream of the *GFP* coding sequence to create an in-frame OsMYB73-GFP fusion under the control of the cauliflflower mosaic virus (CaMV) 35S promoter. The fusion constructs and an empty control vector were transformed into rice protoplasts separately. GFP fluorescence signals was detected using a confocal laser scanning microscope (Leica TCS SP5, Germany; http://www.leica.com).

**Knockout rice *OsMYB73* through CRISPR/Cas9 gene editing system**

The target site (GCCATGGCTACCGTTCCAGT) in the first exon of *OsMYB73* was designed via CRISPR direct website (http://crispr.dbcls.jp) (Naito *et al.*, 2015). We performed the experimental operation according to the instructions for constructing vector of VK005-01, The gRNA was controlled by the rice U6 promoter, whereas *mpcas9* by the *Ubiqutin* promoter and the *hygromycin* resistance gene (*Hyg*) by the CaMV 35S promoter, all in the VK005-01 vector (Beijing Viewsolid Biotech Co., Ltd., http://www.v-solid.com/Catalog.No.VK005-01, China). Then we sent the successfully sequenced vector (plasmid or bacterial) to Jiangsu BIOGLE Gene Technology Co., Ltd. for *Agrobacterium*-mediated transformation. Then we received the T_0_ generation transgenic plants from company, and we determined the positive plants by testing hygromycin gene (Supplemental Table S1). Later we designed sequencing primers near the knockout target site for PCR amplification and sent to company for sequencing. We determined genotypes and mutation types according to the comparison with the wild-type target sequence and observation of the sequence peak map of the mutation site. The Sqprimer was used to sequence the vector. The construct was introduced into ZH11 wild-type rice using the *Agrobacterium tumifaciens* strain EHA105 (Hiei *et al.*, 1997). Positive transgenic lines were identified by PCR amplification of a *Hyg* fragment (Primers *Hyg*-F and *Hyg*-R), and the analysis of the genotypes was performed by sequencing an amplified PCR product of a specific transgenic fragment (356 bp) near the protospacer adjacent motif (PAM) sequence (with the primers Seq-F and Seq-R). Primer pairs for PCR amplification are shown in Supplemental Table S1.

**Microscopy observation**

The brown rice of ZH11 wild-type and the *cr-myb73* mutants were cut transversely and vertically with the a sharp blade, and the ruptured transverse surface was coated with gold to prepare samples according to Kang *et al.* (2005). The ruptured transverse and vertical surface was observed by scanning electron microscope (SEM) using a scanning electron microscope (S-3400N, Hitachi, Tokyo, Japan). Microscope scanning of the glume outer surfaces of ZH11 and *cr-myb73-46*, *OE-myb73-3* transgenic rice mature seeds. For the ultrastructure observation of amyloplasts, developing seeds (9 DAF) were fixed over 12 h in 0.2 M phosphate buffer (PH7.2) with 2.5% glutaraldehyde. Samples were treated as described by Takemoto *et al**.* (2002)*,* and sectioned using an ultramicrotome (Power Tome-XL; RMC, http://www.rmcproducts.com). A transmission electron microscope (H-7650; Hitachi, http://www.hitachi.com) was used for observation. All these experiments carried out at Bio-ultrastructure analysis Lab. of Analysis center of Agrobiology and environmental sciences, Zhejiang University, Hangzhou, China.

**Analysis of starch physicochemical characteristics in mature grains**

The total starch content of the milled rice powder was measured using a starch assay kit (Megazyme, Wicklow, Ireland; http://www.megazyme.com). Amylose content was determined following the method depicted by (Liu *et al.*, 2009). Lipid and protein contents in the grains were measured according to the method described by Kang *et al.* (2005). To determine the starch pasting properties, 3 g of milled rice power (0.5 mm or less, 14% moisture basis) was transferred into a container with 25 mL of distilled water. The sample was mixed and measured with a Rapid Visco Analyzer (RVA Techmaster, Newport Scientific, Narrabeen, Australia), applying the protocol described by the manufacturer. To determine the chain length distribution of amylopectin, 5 mg of rice flour was digested with Pseudomonas amyloderamosa isoamylase (Megazyme), then analyzed using the capillary electrophoresis (PA800 plus pharmaceutical analysis system, Beckman Coulter, USA; http://www.beckmancoulter.com). The swelling and gelatinization properties of endosperm starch in urea solution were measured according to the method described by Nishi *et al.* (2001). Total soluble sugar content was quantified using the phenol-sulfuric acid method (Nishi *et al.*, 2001). In short, 30 mL ddH2O was added into screw-capped tubes containing about 50 mg brown rice flour. Samples were incubated in a boiling water bath for 20 min, and filtrated, and then the volume was adjusted to 100 mL. One mL each sample and a series of glucose standards were mixed with 5 mL phenol-sulfuric acid, and incubated in boiling water bath for 10 min. Total soluble sugar content was calculated with the absorbance values recorded at 620 nm using a spectrophotometer (DU800, Beckman Coulter, USA). Gel consistency and alkali values were determined and analyzed according to the China Agriculture Industry Standard NY/T147-88 (Agricultural Industry Standard of China, 2002). Thermal characteristics were measured with a Modulated Differential Scanning Calorimeter (MDSC, DSC1 STARe system, METTLERTOLEDO) as described by Kweon *et al.* (2000) with minor modifications. About 5 mg dry grain flour samples were placed in a sample tray, and 10 L ddH2O was added with gently mix. The tray was then sealed and subjected to heat treatment for 5.5 min from 35 ºC to 90 ºC, increasing 10 ºC per minute. An empty tray was used as reference. Differences in thermal characteristics between the ZH11, *cr-myb73-35* and *cr-myb73-46* were shown using MDSC curves. To determine the starch gelatinization properties, the grains of wild-type and cr-myb73 mutants were soaked with 1.7% KOH for 30 ℃ 23 h, analyzed according to the China Agriculture Industry Standard NY/T147-88 (Agricultural Industry Standard of China, 2002). All assays were done with three biological replicates.

**mRNA library construction and sequencing**

Total RNA was extracted using Trizol reagent (Invitrogen, CA, USA) following the manufacturer's procedure. The total RNA quantity and purity were analysis of Bioanalyzer 2100 and RNA 1000 Nano LabChip Kit (Agilent, CA, USA) with RIN number >7.0. Poly(A) RNA is purified from total RNA(5 ug) using poly-T oligo-attached magnetic beads using two rounds of purification. Following purification, the mRNA is fragmented into smallpieces using divalent cations under elevated temperature. Then the cleaved RNA fragments werereverse-transcribed to create the final cDNA library in accordance with the protocol for the mRNASeq sample preparation kit (Illumina, San Diego, USA), the average insert size for the paired-endlibraries was 300 bp (±50 bp). And then we performed the paired-end sequencing on an IlluminaHiseq4000 at the (LC Sceiences,USA) following the vendor’s recommended protocol.

**Sequence and primary analysis**

A cDNA library constructed by technology from the pooled RNA from brain samples of pig was sequenced run with Illumina 4000 sequence platform. Using the Illumina paired-end RNA-seq approach, we sequenced the transcriptome, generating a total of millon paired-end reads of bp length. This yielded gigabases (Gb) of sequence. Prior to assembly, the low quality reads(1,reads containing sequencing adaptors; 2,reads containing sequencing primer;3, nucleotide with q quality score lower than 20) were removed. After that, a total of G bp of cleaned, paired-end reads were produced. The raw sequence data have been submitted to the NCBI Short Read Archive with accession number .

**RNA-seq reads mapping**

we aligned reads of sample A and sample B to the UCSC (http://genome.ucsc.edu/) homo sapiens reference genome using HISAT package, which initially remove a portion of the reads based on quality information accompanying each read and then maps the reads to the reference genome. HISAT allows multiple alignments pe read (up to 20 by default) and a maximum of two mismatchs when mapping the reads to the reference. HISAT build a database of potential splice junctions and confirms these by comparing the previously unmapped reads against the database of putative junctions.

**Transcript abundance estimation and differentially expressed testing**

The mapped reads of each sample were assembled using StringTie. Then, all transcriptomes from Samples were merged to reconstruct a comprehensive transcriptome using perl scripts. After the ﬁnal transcriptome was generated, StringTie and edgeR was used to estimate the expression levels of all transcripts. StringTie was used to perform expression level for mRNAs by calculating FPKM. The differentially expressed mRNAs and genes were selected with log2 (fold change) >1 or log2 (fold change) <-1 and with statistical significance (p value < 0.05) by R package.

**Sample preparation and extraction**

Biological samples are freeze-dried by vacuum freeze-dryer (Scientz-100F). The freeze-dried sample was crushed using a mixer mill (MM 400, Retsch) with a zirconia bead for 1.5 min at 30 Hz. Dissolve 100mg of lyophilized powder with 1.2 ml 70% methanol solution, vortex 30 seconds every 30 minutes for 6 times in total, place the sample in a refrigerator at 4 °C overnight. Following centrifugation at 12000 rpm for 10 min, the extracts were filtrated (SCAA-104, 0.22 μm pore size; ANPEL,Shanghai, China, http://www.anpel.com.cn/) before UPLC-MS/MS analysis.

**UPLC Conditions**

The sample extracts were analyzed using an UPLC-ESI-MS/MS system (UPLC, SHIMADZU Nexera X2, www.shimadzu.com.cn/; MS, Applied Biosystems 4500 Q TRAP, www.appliedbiosystems.com.cn/). The analytical conditions were as follows, UPLC: column, Agilent SB-C18 (1.8 µm, 2.1 mm*100 mm); The mobile phase was consisted of solvent A, pure water with 0.1% formic acid, and solvent B, acetonitrile with 0.1% formic acid. Sample measurements were performed with a gradient program that employed the starting conditions of 95% A, 5 % B. Within 9 min, a linear gradient to 5% A, 95% B was programmed, and a composition of 5% A, 95% B was kept for 1 min. Subsequently, a composition of 95% A, 5.0 % B was adjusted within 1.10 min and kept for 2.9 min. The flow velocity was set as 0.35 ml per minute; The column oven was set to 40 °C; The injection volume was 4 μl. The effluent was alternatively connected to an ESI-triple quadrupole-linear ion trap (QTRAP)-MS.

**ESI-Q TRAP-MS/MS**

LIT and triple quadrupole (QQQ) scans were acquired on a triple quadrupole-linear ion trap mass spectrometer (Q TRAP), AB4500 Q TRAP UPLC/MS/MS System, equipped with an ESI Turbo Ion-Spray in-29terface, operating in positive and negative ion mode and controlled by Analyst 1.6.3 software (AB Sciex). The ESI source operation parameters were as follows: ion source, turbo spray; source temperature 550 °C; ion spray voltage (IS) 5500 V (positive ion mode)/-4500 V (negative ion mode); ion source gas I (GSI), gas II(GSII), curtain gas (CUR) were set at 50, 60, and 25.0 psi, respectively; the collision-activated dissociation(CAD) was high. Instrument tuning and mass calibration were performed with 10 and 100 μmol/L polypropylene glycol solutions in QQQ and LIT modes, respectively. QQQ scans were acquired as MRM experiments with collision gas (nitrogen) set to medium. DP and CE for individual MRM transitions was done with further DP and CE optimization. A specific set of MRM transitions were monitored for each period according to the metabolites eluted within this period.

**PCA**

Unsupervised PCA (principal component analysis) was performed by statistics function prcomp within R (www.r-project.org). The data was unit variance scaled before unsupervised PCA.

**Hierarchical Cluster Analysis and Pearson Correlation Coefficients**

The HCA (hierarchical cluster analysis) results of samples and metabolites were presented as heatmaps with dendrograms, while pearson correlation coefficients (PCC) between samples were caculated by the cor function in R and presented as only heatmaps. Both HCA and PCC were carried out by R package pheatmap. For HCA, normalized signal intensities of metabolites (unit variance scaling) are visualized as a color spectrum.

**Differential metabolites selected**

Significantly regulated metabolites between groups were determined by VIP >= 1 and absolute Log_2_FC (fold change) >= 1. VIP values were extracted from OPLS-DA result, which also contain score plots and permutation plots, was generated using R package MetaboAnalystR. The data was log transform (log2) and mean centering before OPLS-DA. In order to avoid overfitting, a permutation test (200 permutations) was performed.

**KEGG annotation and enrichment analysis**

Identified metabolites were annotated using KEGG Compound database (http://www.kegg.jp/kegg/compound/), annotated metabolites were then mapped to KEGG Pathway database (http://www.kegg.jp/30kegg/pathway.html). Pathways with significantly regulated metabolites mapped to were then fed into MSEA (metabolite sets enrichment analysis), their significance was determined by hypergeometric test’s p-values.

**Yeast two-hybrid assays**

The AD and BD fusion constructs were co-transformed into yeast strain AH109, interactions were tested on SD/-Leu-Trp-Ade-His (QDO) medium with X-α-gal and 50 mM 3-amino-1,2,4-triazole (3-AT) at 30℃ for sevel days after plaques appeared in SD/-Leu-Trp (DDO) medium.

**Yeast one-hybrid assays**

The AD fusions were co-transformed into yeast strain EGY48, with the *LacZ* reporter gene driven by *OsISA2* and *OsLTPL36* promoter fragments and spread on SD/-Trp/-Ura medium. After three-days of incubation at 30℃, positive clones were transferred to SD/-Trp/-Ura medium with X-gal for blue color development. Relative β-galactosidase activity was quantified by hydrolysis of ο-nitrophenyl-β-D-galactopyranoside (N1127, Sigma). The absorbance for the released ο-nitrophenyl compound was measured on a spectrophotometer at 420-nm.

**Dual-luciferase reporter assay for transactivation analysis**

For transcription activation analysis in rice protoplast, the coding regions of *OsMYB73* was inserted into the vector none to construct effector vector, a 2 kb fragment of the *OsNF-YB1, OsISA2, OsLTPL36* promoters were inserted into the vector 190-LUC to construct reporter vectors, respectively. As a control, the renilla luciferase (REN) gene was used.co-transformation luciferase values were measured using the Dual-luciferase reporter assay system (BiYunTian) in a Promega Glomax chemiluminescence detector.

**ChIP experiment and high through-put sequencing and data analysis were conducted by Seqhealth Technology Co., LTD (Wuhan, China).**

**ChIP assay,library preparation and sequencing**

ChIP assay was performed on the filling rice endosperm by SeqHealth (Wuhan, China).The tissue/cell was fixed in 1% formaldehyde for 10 min at room temperature by a vacuum pump, after which 0.125 M glycine was added and the mixture was sat for 5 min to terminate the crosslinking reaction. The tissue was then collected and frozen in liquid nitrogen, and grinded by tissue lyser. The grinded powder/cells was treated with cell lysis buffer and nucleus was collected by centrifuging at 2000g for 5min. Then, nucleus was treated with nucleus lysis buffer and sonicated to fragment chromatin DNA. The 10% lysis sonicated chromatin was stored and named “input”, and 80% was used in immunoprecipitation reactions with anti-OsMYB73 antibody and named “IP”, and 10% was incubated with rabbit IgG (Cell Signaling Technology) as a negative control and named “IgG”，respectively. The DNA of input and IP was extracted by phenol-chloroform method. The high-throughput DNA sequencing libraries were prepared by using VAHTS Universal DNA Library Prep Kit for Illumina V3 (Catalog NO. ND607, Vazyme). The library products corresponding to 200-500 bps were enriched, quantified and finally sequenced on Novaseq 6000 sequencer (Illumina) with PE150 model.

**Data analysis**

Raw sequencing data was first filtered by Trimmomatic (version 0.36), low-quality reads were discarded and the reads contaminated with adaptor sequences were trimmed. The clean reads were used for protein binding site analysis. They were mapped to the reference genome of rice using STAR software (version 2.5.3a) with default parameters. The RSeQC (version 2.6) was used for reads distribution analysis.The MACS2 software (Version 2.1.1) was used for peak calling. The bedtools (Version 2.25.0) was used for peaks annotation and peak distribution analysis. The differentially binding peaks were identified by a python script，using fisher test. The Homer (version 4.10) was used for motifs analysis. Gene ontology (GO) analysis and Kyoto encyclopedia of genes and genomes (KEGG) enrichment analysis for annotated genes were both implemented by KOBAS software (version: 2.1.1) with a corrected P-value cutoff of 0.05 to judge statistically significant enrichment.

**Transcription factor (TF)-centered yeast one-hybrid (Y1H) assay**

**Construction of a random short DNA sequence insertion library**

Y1H assay was performed using Matchmaker One-Hybrid System of Clontech (Clontech™, Palo Alta, CA, USA). For insertion of a random DNA fragments in pHIS2, three single-stranded DNA sequences were synthesized, named as Y1, Y2 and Y3. Y1: “CTCACTATAGGGCGAATTCCCANNNNNNCGGGGAGCTCACGCGTTCGCGA”; Y2: “CTCACTATAGGGCGAATTCCCC(T)NNNNNNCGGGGAGCTCACGCGTTCGCGA”; and Y3: “CGCGAACGCGTGAGCTC”. The underlined ‘Ns’ are random DNA sequences that were used to determine the cis-acting element motifs recognized by a certain TF. The flanking sequences of the underlined DNA sequences are same as the two flanking sequences of the Sma I site in pHIS2. PCR was performed using Y1 and Y2 as templates, and Y3 as the primer. The PCR reaction system included: 2 μL of Y1 or Y2 (10 μM), 0.5 μL of dNTP (10 mM each), 3 μL of Y3 (10 μM), 1 μL PCR buffer, and 0.5 U ExTaq (Takara, Dalian, China) with the volume of 10 μL. The PCR reaction conditions were as follows: 94 °C for 90 s, 55 °C for 15 min, 50 °C for 30 min (one cycle). The pHIS2 vector was Sma I (Promega) digested and the digested vector was purified by agarose gel electrophoresis (0.8 % agarose). For addition of a single ‘T’ base at the linear pHIS2 terminals, the following reagents were included in the PCR: 1 μg of purified pHIS2, 0.5 μL of dTTP (10 mM), 1 μL 10× PCR buffer, and 0.5 U ExTaq (Takara), in 10 μL. The PCR was carried out at 74 °C for 30 min, and was purified by agarose gel electrophoresis, to obtain the T-vector of pHIS2. The T-A ligation system includes 0.2 μg of T-vector of pHIS2, 0.5 μL of PCR production (PCR products of Y1 + Y3 or Y2 + Y3), 1 μL of 10× ligation buffer, 3 U of T4 ligase (Promega), PEG6000 at final concentration of 10 % (w/v). The ligation conditions were 12 °C for 20 h, then 10 U Sma I (Promega) was added, and incubated at 25 °C for 4 h to linearize the empty pHIS2. The two ligations (Y1 + Y3 and Y2 + Y3) were then mixed and the mixture was transformed into DH5α Escherichia coli competent cells using heat shock. After incubation at 42 °C for 90 s, 1 mL of LB medium was added to the transformation mixture, which was incubated at 37 °C for 1 h, then 10, 50 and 100 μL of transformation mixture were plated to evaluate the transformation efficiency. LB medium (4 mL) was added to the remaining reactions. After incubation at 37 °C for 14 h, the culture was used for plasmid isolation. The isolated plasmids formed the random DNA insertion library, which was used for screening the DNA motifs recognized by a definite TF. The ORF of *OsMYB73* was cloned into pGADT7-Rec2 (designated as: pGADT7-*OsMYB73*) at the site between the SMART III Sequence and the CDS III Sequence using the infusion method, following the procedures in the user manual (In-Fusion® HD Cloning Kit).

**Screening the random DNA insertion library**

The following were combined into a sterile 15 mL tube: 2 μg pGADT7-*OsMYB73*, 1.5 μg of the random DNA insertion library, 10 μL of Herring Testes Carrier DNA. The mixture was added to 600 μL of competent Y187 yeast cells. The transformation and selection methods were performed according to the manufacturer’s protocol (BD Matchmaker™ Library Construction & Screening Kits User Manual). The positive clones were selected on the SD/-His/-Leu/-Trp (TDO) medium supplied with 30 mM 3-AT (3-Amino-1, 2, 4-triazole). The positive clones were further selected on the high stringency selection mediums (supplied with 80 mM 3-AT) to select the clones having high binding affinities to *OsMYB73*. For all the Y1H assay in this study, the primary culture of yeast transformants were transferred into fresh medium for further culture, and were grown to about 0.6 at OD600 before spotting. The densities of yeast cultures were measured at OD600, and each transformants was adjusted to equal density for spotting. The positive transformants were further confirmed by spotting yeast cells with serial dilutions (1/1, 1/10, 1/100, 1/1,000) onto TDO medium supplied with 50 mM 3-AT. The transformants grown at SD/-Leu/-Trp (DDO) were used as growth positive controls.

**Analysis of the insertion sequences of positive clones**

The pHIS2 plasmids were rescued from the positive clones identified by TF-centered Y1H analysis, and sequenced. The insertion sequences were analyzed using PLACE (http:// [www.dna.affrc.go.jp/PLACE/)](http://www.dna.affrc.go.jp/PLACE/)) and PlantCARE (http://bioinformatics.psb.ugent.be/webtools/plantcare/html/) to identify whether they were known motifs.

**Determination of the novel motifs recognized by rice *OsMYB73***

A 7 bp random motif library from yeast strain Y187 was purchased from Nanjing Ruiyuan Biotechnology Co., Ltd. (Nanjing, China). A TF-centered Y1H assay was performed as previously described. The yeast motif library was incubated overnight, after which an Ex-Yeast Transformation Kit was utilized to generate competent cells. Then, 35 μg of pGADT7-*OsMYB73* was transformed into competent yeast library cells. The yeast was transferred to plates of SD/-His/-Trp/-Leu media supplemented with 50mM 3-AT and allowed to grow at 28 °C for 5 days. Monoclonal colonies were selected for sequencing, and random motif sequences between “GGG” and “CCC” (the SmaI site) were screened. The insertion sequences were analyzed using PLACE2 and PlantCARE3 to identify whether they were known motifs.

**Data analysis**

Raw sequencing data was first filtered by Trimmomatic (version 0.36), low-quality reads were discarded and the reads contaminated with adaptor sequences were trimmed. The clean reads were used for protein binding site analysis. They were mapped to the reference genome of rice from *oryza sativa japonica* (Nipponbare, IRGSP1.0) using STAR software (version 2.5.3a) with default parameters. The RSeQC (Version 2.6) was used for reads distribution analysis.The MACS2 software (Version 2.1.1) was used for peak calling. The bedtools (Version 2.25.0) was used for peaks annotation and peak distribution analysis. The differentially binding peaks were identified by a python script，using fisher test. The Homer (version 4.10) was used for motifs analysis. Gene ontology (GO) analysis and Kyoto encyclopedia of genes and genomes (KEGG) enrichment analysis for annotated genes were both implemented by KOBAS software (version: 2.1.1) with a corrected P-value cutoff of 0.05 to judge statistically significant enrichment.

**Bimolecular fluorescence complementation (BiFC) assay**

Two pairs of constructs *OsMYB73*-VN173 and *OsNF-YB1*-VC155, were transformed into tobacco leaf cells as described previously. The mixture of modified pUC-SPYNE and pUC-SPYCE vector was used as a negative control. After incubation overnight in the dark at 25 °C, the fluorescence was observed using a FV1000 MP two-photon laser scanning fluorescence microscope (Olympus).

**Promoters analysis and motifs prediction**

The promoters sequences 2.0 kb upstream of the translationstart sites of candidate genes were downloaded from the EnsemblPlants website (http://plants.ensembl.org/). The *OsNF-YB1* binding sites (CCAAT-box) were identified using PLANTPAN v.3.0 (http://plantpan.itps.ncku.edu.tw/) and NEW PLACE (http://www.dna.affrc.go.jp/PLACE/). The *OsMYB73* binding sites (GGTAGGT and CCGTTA) were identified using JASPAR website (https://jaspar.elixir.no/).

**Purification of tag-fused proteins**

For the recombinant protein expression, the CDS of *OsNF-YB1* and *OsMYB73* were amplified and cloned into pET28a (Merck, Darmstadt, Germany) and pGEX-4T-1 (GE Healthcare, Chicago, IL) respectively. HIS-OsNF-YB1, GST-OsMYB73 recombinant proteins were induced in E. coli strain Rossetta, and purified by Glutathione-Sepharose Resin Protein Purification Kit and 6 X His-Tagged Protein Purification Kit (CWBIO, Beijing, China) respectively.

**Electrophoresis mobility shift (EMSA) assay**

A 5'-biotinylated oligonucleotide was used as the probes. The probes were incubated with the nuclear extract at room temperature for 30 min. The entire reaction mixture was run on a non-denaturing 0.5×TBE 6% polyacrylamide gel for 1h at 60 V at 4°C and then transferred onto Biodyne® B nylon membranes (Pall Corporation). Signals were visualized with reagents included in the kit and ChemiDoc XRS (Bio-Rad Laboratories, UAS).

**References:**

LaSarre B, Federle MJ. EMSA Analysis of DNA Binding By Rgg Proteins. *Bio Protoc*. 2013;3(15):e838.

Li W, Wang K, Chern M, et al. Sclerenchyma cell thickening through enhanced lignification induced by *OsMYB30* prevents fungal penetration of rice leaves. *New Phytol*. 2020;226(6):1850-1863.

Li W, Zhu Z, Chern M, et al. A Natural Allele of a Transcription Factor in Rice Confers Broad-Spectrum Blast Resistance. *Cell*. 2017;170(1):114-126.e15.

Rauluseviciute I, Riudavets-Puig R, Blanc-Mathieu R, Castro-Mondragon JA, Ferenc K, Kumar V, Lemma RB, Lucas J, Chèneby J, Baranasic D, Khan A, Fornes O, Gundersen S, Johansen M, Hovig E, Lenhard B, Sandelin A, Wasserman WW, Parcy F, Mathelier A JASPAR 2024: 20th anniversary of the open-access database of transcription factor binding profiles. *Nucleic Acids Res*. in_press; doi: 10.1093/nar/gkad1059

Supplementary sequence for EMSA

Rice *Osmyb73* transcription factor-CDS：

ATGCTTCCAACCTCAAAGAACAAGTTGATGGCGGCTGCAGGTAACTCCTTCCTCAACCAA

GATCCGACGATGATGGGATCTGGGATGCCGCATATGTACTTTGCAAGCTCTAGCCATGGC

ACCGGTGCCCACTACCAAAGTCCTGGTGGTGCTCCCATCACCATGGCAGTCCCGGACATG

GGGTTTCTTGTTGCAGGGATAGGCATAGCTCCATCTAGCTTTGTGATGCCAGAGGGGGCC

CTAGCAGCTAGCTATAGCGCCATGGCTACCGTTCCAGTAGGGGTTGTTGTTCCTCAACAG

CAGTCGTCACGATTTGGGGGCAACAATGGTAACCCTGGGTCGTTCAAAGGAGCATGGACA

AGGCAAGAAGATGAAGTTCTCAAACAAATGGTGATACTCCATGGAGATCGTAAGTGGGCA

ACGATTGCAAAGAGCCTCCCAGGTCGGATCGGGAAGCAGTGTCGCGAAAGATGGACCAAT

CATTTGCGCCCAGACATCAAGAAGGACGTCTGGACTGAGGAGGACGACAGGATGCTGATC

GAAGCACACAAGACTTATGGGAACCGTTGGTCAGTAATCGCAAGATGCCTCCCTGGCCGG

TCGGAGAATGCTGTCAAGAACCATTGGAACGCGACTAAGCGGAGCCTGAAGTCGAAGCGT

CGGATGAAGAAGAAGAGCGTGCAAGTGGTGAATTCACCGCCGGGGCAGCTCTCCCCCCTT

GAAGAGTACATCCGCAGCCAGTACCCGTCGGCCGTTGAGACCACACCGCCGCCACCGGCC

GTGCCTGCACCGCCTTCCGACGTCATTGTGCACGGTGCCGGATCGGTCAGCGCAGGTCCA

ACCGTTGCCACACAGGAGCCCACCGGCACCAACCCATCGGAAATGGGGATCTACCTTGGC

CTGGGTAACCCGGCTGGACCGACCACCCAGCAGCTAGCGGCGATGAACCTGAACATGTCA

CTGGCGCCGGACCTCAACGCCTACAACGATCAGCGAGAGGGGTACTACCTCCCGTTCGTC

CCGCAGGGCAACCTGCACTATGGGATGCATGTGCCGGCACCGCCGGTGCAGCAGCAGCAG

CAGCAAGGGATTAGCGTTGATCAGGGCCTGCATTCTTCTTGCCTGAGCCTGTACCACCCG

TTCCCGGGAACCCACCCGGTTAGCCTGGATTTCGGTTGCCAATCCAGCAACCATGCCAAC

GCAGGTGGCTACTACAGTGAAGCTGGCCCAAGCAGCGGCAGTGGCAGCGGCGACCCTGAC

GACGTCGACGTCATCCAGATGGCCTCCAGGCAGTTCCTGATGCCGTCCGAGGCTGAGGTC

ACTCTCGACCTGACCAGGTTCAAGTGA

MYB-binding motifs (JASPAR website)：GGTAGGT and CCGTTA

Rice *OsISA2*-2kbp-promoter：

TCTTTTTTGGTGGCGTGGTTGGGGTCGGAGGGGAAGTCAATGTCGTCCAAGTCCCGGTTGCCATAGACGGCGGTGTCGGGGAAGGAGAAGGAGGCGGCGGCAGAGGGGTCATCGACGATGGTGGGGAGTGTCAGCGCACGCCTCCATGAGTCTGTGACGGACAAGCTCCCCGACTGACCGACTCCTCTTCACCAATGACTTCCCCACGGCGGTCACTGACGGGGCGGTGAGGGTAGCTTGACACAGAGGACAGAGGCGGGTTGGGCGGAGGAGGCACCCAAAACCCCCCAAGAAAACGCCGCCTTTTCTTGCCTTGCACAGCTCATCGATCCGTCGTCGGTGAGATCCCTTCGGTTTTTGCGTGTGCAAGATTGTGTCCGCAGGATTTATGAGGTGAGAGATAGCGACTGTGAGGGTCTTGATTTGATCTGTTGTTGCTGTTATTGTTGCGGTGGGGGTGGTGGTGGTTGGGCGCCATGGTGGCACTGGGAGGGCCAGAGCAACGGCAGAAGGACAGGTTCTTCTAGGGCACATAGGAGGTGCATGAACAGGGAGGCCTTGGTGACTTGCGAGGAGACAGGGCTCGTGACGAGGCCAACAAGTGCGCCACACATGTTGTGGGCTCTCTAGATGGCCTCTAGGGTGTGGCGGTCGCCGGCGGAGGCGAGCTCACGGAGCACGACGGCGTCGCTCATCCGCGCGGCCAAGATCTTCACGAGCGCACCGAAGACGGCGTTGGCGGCGCCCTCCACGAGCTCGAGGCGGCGGAGCTCGGCTGGGCTCGCAGGCCTCCTAGCTCGCTGAAGAAGAGTAGAGAAGAAAGAAAAGAAAAGAGATGAGAGGAGAAAAAGAAAAGAAAGAAGAGATGCAGTCTCCACTTTACTCACTTATATGTGGGCCCCATAAAAATTTAATTTTTATTTTGCTGACTAGGATGCTACGTTAGCAAAACCAGATATACATACTGCTATAAGACCTAAATTATACGGTTTTATATAGTTTAGGGGTAAAGATTATGGATGTCATACAACTCGATGAAAAGTTGAGGGACGATTTCTGAACTTATTCCAAAAATCTTTGCAATGGCCCAGCCTGTTGATGAAATGGATCGACAACGGTCAGCCCAGCCCAGAAATCCAGTAGACCGTAGACAAATAGCGAAAATCAGCTGAGAGAACTGCTGGCTGTTGACCTGTTATGTTCGCGAACGGCAGAATTTGCAGCTGGAAACTGATCGATCAAAGAGATCCGTGCAACGTGCAGGCTCGAGCGGCACATTGGGCGGACGATAAATCTCCACCCGTTTCTTGGATCTTGTCACTTTTGGAAACCACCATACACCACACCACGTGATCACGTAGCGAGTACATGTGCTACTTGCAGCGGTCTACGCCACCGGACAGCAGGACGACGCCACGATCACATCTCCACTCCACATCGTGACGGGGACGGGGCCCAGTGCGCGCGAACAGCTGCTGTACATGGGACACCCACGCGAAACGGACGGATGCGGCCGCACCACCCCGCCGCGCCGAGCGAGCCTGTGTGCACTCGCACCTGCACTGTGCGGTGACAGCGACACCAACGGTGCCGCTGCGCGACGGCGTCACCTGCGACGCAAATGAACAAACCAACCCCCCCCCCCCCCCCCCTCTTCTTCACGAAATAGCCGTGCGAAAAGCGACGCCCTCCCAACGCAAACGCACGCAACGTCGTGGCACACAACGGATCCCGTGCGCGCCGCCCACCGTCGCCCGCGCGCCCATCCCGCTCTCCGCCTCCACGTGTGCCGCCCACCGCGCGCGCCTCCCCCCTCGCCTCGGCTCCTCCCTCCACTCCACTCCACGCCCCCGCGCGTGCCGCGCGCTCTCTCCCCACCCTACGCGGCCCTTCCCTTACAAGCGACCGGGCCCTGCTGAAACCCCGATGCCCACCACCCGCTCGACGCAATTCCGCGCCCGCTGATCGCACCGTCAGGTCCGGTCAGCCAGCCTCAGCCC

Probe 1: 5’biotin-TCCCCACCCTACGCGGCCCT

Competing probe 1: TCCCCACCCTACGCGGCCCT

Mutation probe 1: 5’biotin-TCCCCACAAAAAGCGGCCCT

Rice *OsNF-YB1*-2kbp-promoter:

ATAGCATTTTGTTTTATTCTCCCCTTTTGAAAACAGTATGCAGGGGTGATGCTTGTTCATTATAAGAGGCAAAGGGTAGCATATGATATATAAGTTTATAATTTATTATAGATGACAACCTCAAAGGTAACTATCGTTGACATATGTAAATTATGCTACATTACAAAAGAAATAATGTGAACATTGACATTGGTACATACGTGGACATTATCTATAATTATAGACTGACATCTACATTCCCTATGGTTATCAATCGGAATGTCGGCTCTTGTAGCCTTGTGAACATACGTACTCTCTCGCACTATATGTGCATTGATGTGAATCCTCCAATTTGCTATAGGAATGTCGTAGGTTTGAGTAGCCTATGTATTCATTGTCAGCAAATGCGCACCGTGAACTCTTTACGTCAGAGGATCTACGTCAGTCCGCTAGTACGCTCATGACATATTCCCCCTGTTTTTGAAGATGCGGTTAGTTACTCCTTCTGTACACTCCATAAAATGTGCTATCCTACAATACATTCCCCGCTTCAATGTATGAGATATTTTTCCCCATTTTGTTAGAAAATACTTAAGCAATATGTTCCAAATATAATGTTATATTGGGTTGATGTTGTGTTAAAATGGAGTGGTTAAGCATGAGCCTTCATTTCCCTCATAACTGTTTTTTTAGAACTCCTCGCAACTGTTGCACTCAACAATTTCATTTTCCTTCCTGTTATTCAATTAAGGTTCTCCTTAAAAATAAGTCTTTCATGAATAATTTAGTATTTTGAAGGTACTATTGAAAGAATTATTTGGAGATGTCTAATGCTTGAATGATAAGTAATTTGGAAGGCATCTACTCCTACAAAGTTGAATCTTCTAAATACTCCTTCGCTTTTAAATATACGGCATTGTTAACTTTTGAATATGACAATATTTAACAATTTATCTTGTACAAAGATTTAGTGCAAATATGCACAATGCGTAAAGTACCTTTAATGTAAAACAAGCCACAACAAAACAAATGATACTTAGAGCATTTTTTAATAAGCCGATTGGTCGCATCATGTTTAAAAGTCAACCATGTCATATATAAAAAAAGGGAGATAGTTTCATACATAGCTCTAATATATTTTGAAGGTACTAAATTTTAAAAATTGTTGTTGCAGATTGTAAGTCTTGCATATAATTTATTGTTTCTTTTACTTAGCTTATTAGCTCTAATTAATCACACCCTATCTCTCCTATATTTATATAATAAAAACATCCCGACACACCAACGTACATTTTCTCATCTATTTCTAAGGTATGGATGGTATTGTCATGTCAACTTATCTTCTTTCTAGTTATGCACCCTCCTTAATTATTGTACCAACTTAAAATAAGCCATATATTTTGAAATTATCATACCAACTCACAATATGCCATATATTTTAAAATTATTATGTCAACTCACAATATGCTATATATTTTGATATTATCGTGCCAACTCACAATATACCATATATTTTGAAATGAAGGTCATATTTAAGATGTAGAGTAAATGTATGAAATCCTCCCATCCATATCCTCATTATTAATTGCTTCCACCTACCCCAAGCATTTATAGTTAGAGTAGAACAGTTGTGCTCTTGCACCGAAGGGATGACATGTAGGAGCCATGCCTACGTCCCCTGGCCACCCAAGCCCGTTGAATTAACATGGTCCCTCAACAGAAACATCCATTTTTGATGGTGTGCTTCCTTCTTGGCGACCTTACTGGTAGGTGACATGCTAACAAAAACTGCACAGTCATGCAAGGCAGGGGCCGGTCAAAATTGAAGTCCCTCCTACCTTGATTGTAACGCTAATACTATGGCTCACCGCCTCTGCGGCAATCTGGCAGCCACTTCTCCTCCTCTTGGTGCCACAGCTGCACCTTTGACGTGGCTGGCTGCTTGCATTTGTCCAAGCCGGCGCAGCCCTGATGTAAGTCCTTTGGTATAAATACTGCCCAAGCATAGAGCGAGAGAGAGCCATCACAATCCAATACAAGTTCACAACATAAGCATAGTACAGGTCATTGAGACTTGAGAGAGC

Probe 2: 5’biotin-CTTACTGGTAGGTGACATGC

Competing probe 2: CTTACTGGTAGGTGACATGC

Mutation probe 2: 5’biotin-CTTACTAAAAAAAGACATGC

Rice *OsYUC11*-2kbp-promoter：

GGGCCAAGCTTCGGTTTCTAATTTTCATCTCCAAAAGTTTCTAACTTAATTTATTAATAGTTAATTCCAGGCCCAAAGCAAGTTTTCTCAAAAAATTAAGTTCAGTTTCCTCTTTAACTTCTGGGGAATGTGTTAGTATATTGTCAAACATGTATCTTTCACGAAAGTATGATGACTAAGAGCTCCCAAGTTACATGTTTGACAATATACTTAAAAATGGAAAGGAATCTAGCTCTCACTTGGAGCAATCTATTGTCCTAGTAGAAGGTAGTGTATGTCCTATCAATTAGTTCAAGAATTGCACATAATATTTAGAAAAACAAGGTATTGCAAAAACAAAAGCAGGACAGTGTGCGTATTATCTAGTTCCTACCCCCTCCACTTTTCTAATATTGCCTAAAAAAATGAAGCAATATGTTATCACAACTCACCATGCGTTTTTATACATTTAGTTGTTAAGATAAAGATTTTCTGTTTTTGTGCAGTAGGCAGATGATACAATAATTAGAACAACATACACAAACATGTTTTGCATTACAAGTAGTACGTCATCTCATTAAATTAGGTCGGATTAAAATCACCAAGCATGCATGGAGTTATAACTAATTAGTGCATCAATTCGTAGTAAATAATAGCTCATACATTCACATGAAACTACAAATAATCAATGGTACTAATACATTAAAATAATTAAAGGATACATATTCAAGACTTTGTTTAATATTGTTTATTTTTATTTATTTATTGATAGTATTACTAATTAATTAAATTTAGAACTAATAGAAAATCTACGGAGTTCAAATGTGTATGCCTTACCATATTCCATAATTTTCTTGAGAGACATGTATAGACCGTTAAGGATAGTATCAAAACCACCAAATTAATATGTTTTGTCGGCTAACCGAAAAGGAATACCTGCCAAGTCTAGAGAGCGAGGGAAAGTCCATTTTGCATTTTGTTGGCGGATAACCGCAAAGGTAACAAGCACTTTTAGTTGTCGGGATTTGGCCGCCAAAAAAACTAACGGGCCACCAAGAAAATTCTAGATTCCAGTAGTGATGCTTGTAGAGTAATAAGTTGATATAATGTTAAGATTGCTAAAACCATATATGTTCACTACCATATATATTTAAAATGGATACATATATAAATTAAGATTTTGAGGATACTGTTATTGAGATGGGGAAAATGCAAACCAAATTCACAGTTTACCACATTTCAGTAGTGAAATGCTTTGGATTCGTGTCCACTGCATACAATAAGTTGAACTTTTCAAACAGCTTCATGCCATATTACAACACTTTATGCACTTTAGACAAGAACCTAGTAGAATCCAATTGGTATACGTGCTCTATTTGCTAGCTAATGTGGTAGTAACTGAAGATAAATAAAACAAATCTCCCACCGAAAGTACTTATAAAGAGACATGTTCCTCAAACAAGGCATCCATGCATAATAAAGTTGGTTTTATCTGAAAAAAGCAAACAGAGGCTCTTCCAGTCATTGTAAAAGGTATTTGGATTTTTTTTTTGGGAAACTCACAACACAATGCCCCCCCTTTTATGTCGTATTCAACACTTCCCAATAATCATGACATATAAGATAAGTATTATATACAAGATAAGTATTTTAAAGTATGAATTAATCAATAATTAAATATACAAGTACCACTTAATGATCATGTTAATGTAGAAAAAAATCATTTGTATATGCTCTTTAATTGAGAGGACATATTAAATAAGAAACCTTGTACTTTTGTCTACATATGTGTCATTCTATCTTGGCCGGTAGCATAAATAATGTACTTAATACATGTGAAGTGAATCTGTCCAAAATACATGTAAGAGGGATATGCATTACAGTCAATAGTAACGGACAAATTTCCGTATAAATAGCATGTGCTCTCCGAGAATTTATACATCTCACCAAGAGAAAAAACAACATATACAGAATAGTGTCCCAACATTAGTTTCACTTCCTTCCCTTTCCTTTTATTTTCTC

Probe 3: 5’biotin-GTATAGACCGTTAAGGATAG

Competing probe 3: GTATAGACCGTTAAGGATAG

Mutation probe 3: 5’biotin-GTATAGAAAAAAAAGGATAG

Rice *OsLTPL36*-2kbp-promoter：

TGGTGGAAACAGGTGAAGGCTAGGAGCGAGTGATGATGTGGTCTTCGACGACGGTTGGTGGCGGCCCGGAGGAGATCGGTAGTGGCCACTAATTGGGAAGGGGAGGGAGCACGGGAAGAAAGGTGGGAGGTGGAAGAGATTAGGAGAGGGAGTGCGTGCATGAATCAGGGGATGTGATCAAGGGACCGTGATTAGGGGATCCAGATCGCTCTCTCTTAAAAGGGTGACCTCGATACAAACTCGGTGACGACAGACGAAAAATCATACGGAAGCCTTACGTATGTTTTAAGTAGGTATAGATGTCATGTATCCTATAGATACAGATCTACTACTACGATATGTATTTAATATTTTAATATGTGACCAAATGTTATATTTTTGGGACATGTTTTGATATATCCAACTGATATGCAACTGATCTATGCAATCATTTAAAGTATGATATAATGATATATATTACCGATATACAATATGTGCATATATCAATACAATTAATAACTTATATGATCTATACAAGCATTTAATATATGAGATACTACTGTATCTTACAGATATGCAATATATGCATATATCAATGCAATTAATAACTTATAAGATCTATGCAAGCATTTAATGTATGAGATACTACTTCTCCCTCCGTCCCAAATTAGATGGCATGTATTTTTCCCCTGTTGTCTCAAAACATAAGGCACATTTTCAATTGTTGCAGCTTCTACCCGGTAATGCCCTCTCATTTACATGCATATGTGAATAAATGCTGATAGAGGGCGAGCTGTGCATGCAGCCGGAACGTGGGCCCGCACCGTGTGGCCGTTTGGATTGACTTATCAGCTTCGTAACAGAGCGCTTCAATCTCTATACGAGATTTGCATGCTTAGTTAGTTGATTTGAATGCCCGTTCACATGGATCCTGCTAAATAGGGGTAAAAATAGATAAAACTAAACTGCATGCAAAGTCTTTGGTCTTAGCAAAATAAATTTCACACCCTCTAATTTGGGACGGAGGGACTACTAGCAAAGATGGATCTGATACCAACATGCTTTACATGATCATGAAAGTTGAAAGGCTGCAACTTTGGTACTCCCTCTGTCCCAGATTGGAGGGCGAGTATTTTTTTTCCCCGTTGTCTCAAAACATAAGGCGCATTTTCAAATTTTACACCTTCTACCCGATAATGCCCTTTCATTTACATGCATATGTGATTAAATGCCGCTAGAGGGCGAGCTGGTGCATGTAGCCGGAACGTGGGCCACACCGTGCTGCCGTTTGGATTGACTTATCAACAGAGTGCTTCAATCTATACAAGATTTGCATGCTTGGTTAGTTGACTTGATTGCCCGTTCGAATGGATCCTATTAAATAGAGGTAAAAATGGATAAAACCAAAGCATGCAAAGTCCTTGGTCTTAGCAAAATAAATTTCACGCCCTCTAATTTGGGACGAAGGGAGTATATCTTATAGATATAGTATATATCAATACAATTAATAACTTCTATGCAAGCATTTAATGTATGAGATAGTGTTATATCTTACAGGTATGCAATATATGTATATATCAATGCAATTAATAATTTCTACACAAGAATTTAATGTATAAGATATTGTTATATCTTACAGATATACAATATAATGTATGAGATGCAATATCTTACAGATATGCAATATATGTATATATCGACGTAATTAATAACTTCTACGCAAGCATTTAATGTATAAGATATTGTTATATCTTACAAATATGCAATATAATGTATGAGATGTTATATCTTACAGATATGCAATATATGTATATATCGACGCAATTAATAACTTCTACGCAAGCATCTAATGTATAAGATACTGTTATATCTAATAAATATGCAATATAATGTATGAGATATTATTATATGTTACATATATGCAATATATGTGTATATATCAATGCAATTAATGACCTATAGATAGTAGCATTCAATTTCCACTATAGACCTCCCCTATTTAAAACCCAAGCATCCTAATCAAACCACTCACCACCACTATCAACACAACTCTTGCATCACCATCTGAGAGAAACCAGGGAGATACACACAAGCAATAGCC

Probe 4: 5’biotin-TTTTAAGTAGGTATAGATGT

Competing probe 4: TTTTAAGTAGGTATAGATGT

Mutation probe 4: 5’biotin-TTTTAAGTAGGTATAGATGT
