# Supplementary material for: A novel transcription factor OsMYB73 affects grain size and chalkiness by regulating endosperm storage substances' accumulation‐mediated auxin biosynthesis signalling pathway in rice
Source: Plant Biotechnol J. 2024 Dec 26;23(4):1021–38. doi: 10.1111/pbi.14558 (PMC11933829; doi:10.1111/pbi.14558)
Supplement: Supplementary file 9 — Appendix S2 Rice OsMYB73‐chip‐Seq data and haplotype analysis (PDF file). [file PBI-23-1021-s001.pdf]

OsMYB73-Chip-seq

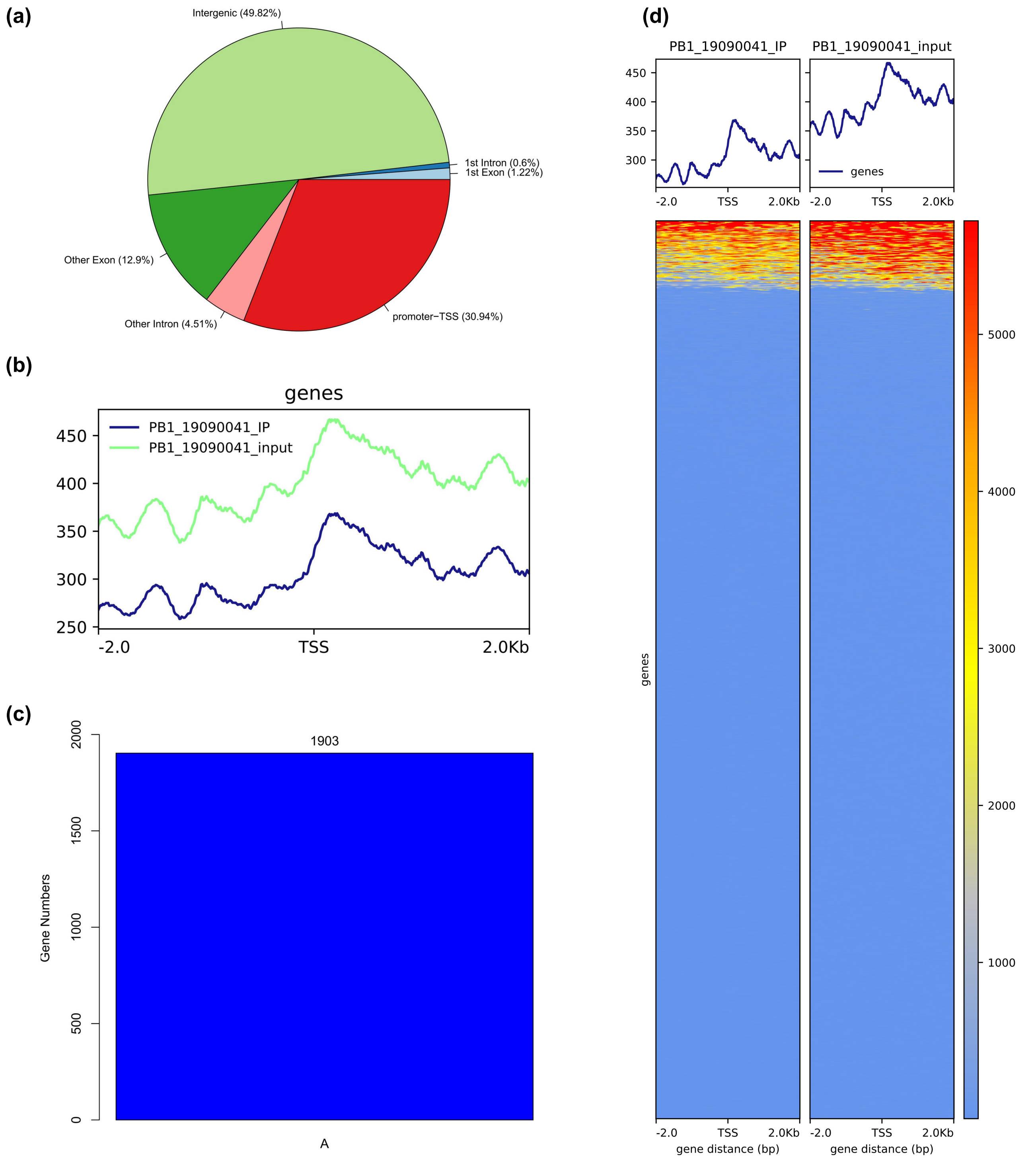

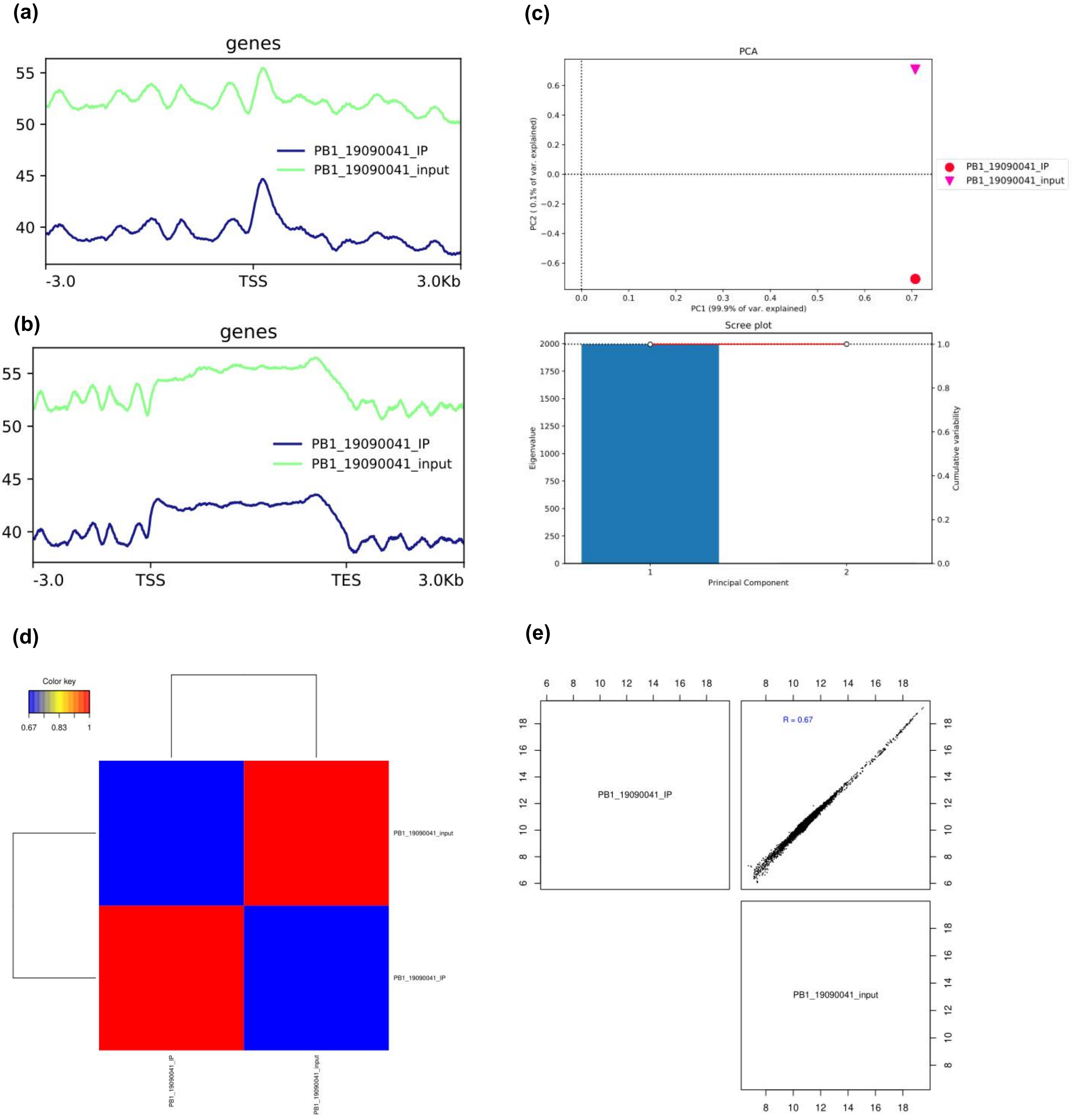
(a)
genes
55
50
45
40
-3.0
TSS
3.0Kb
PB1_19090041_IP
PB1_19090041_input
(b)
genes
55
50
45
40
-3.0
TSS
TES
3.0Kb
PB1_19090041_IP
PB1_19090041_input
(c)
PCA
PC2 (0.1% of var. explained)
PC1 (99.9% of var. explained)
PB1_19090041_IP
PB1_19090041_input
Scree plot
Eigenvalue
Cumulative variability
Principal Component
(d)
Color key
0.67
0.83
1
PB1_19090041_input
PB1_19090041_IP
(e)
R = 0.67
PB1_19090041_IP
PB1_19090041_input

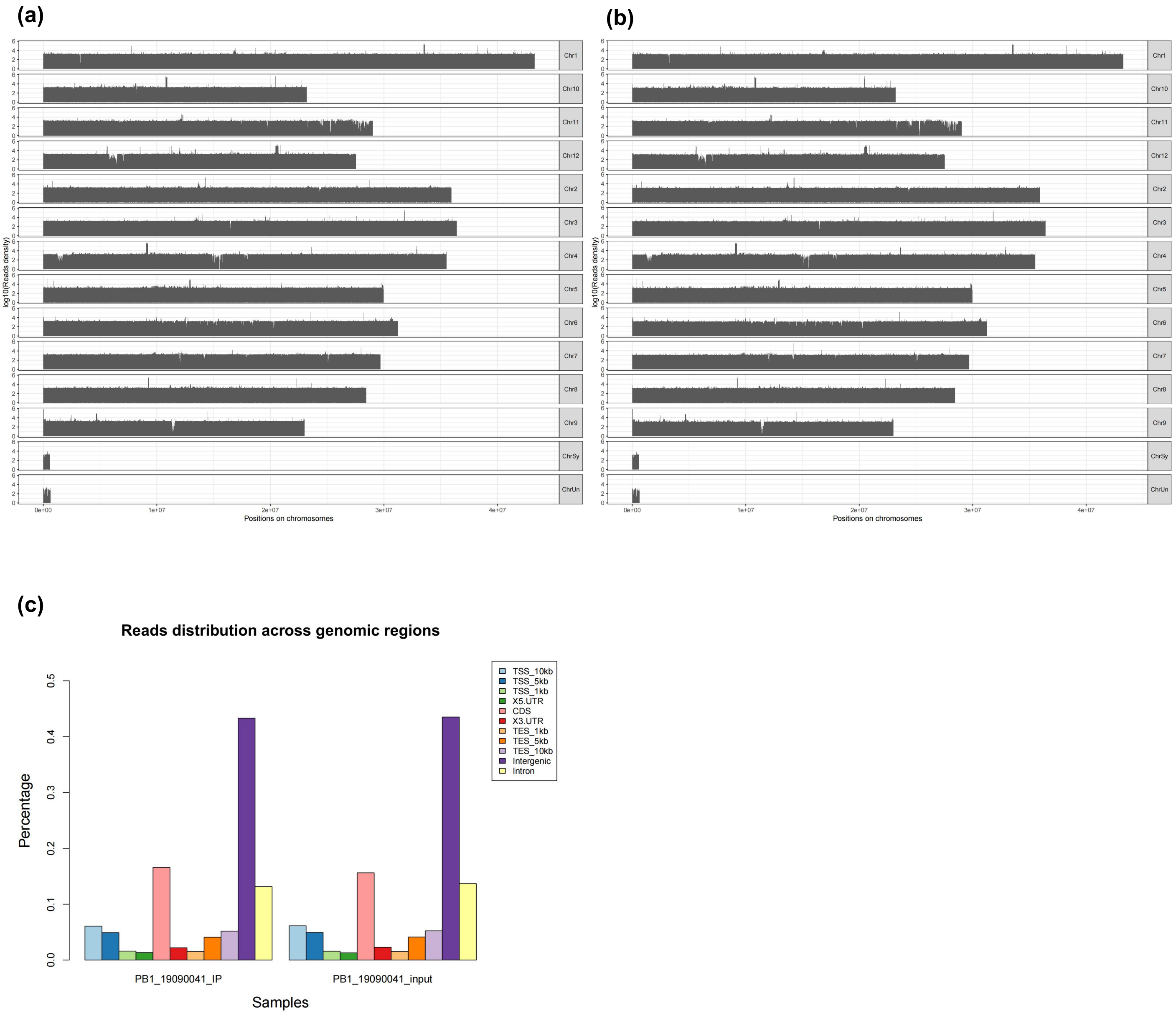

(a)
(b)
log10(Reads density)
Chr1
Chr10
Chr11
Chr12
Chr2
Chr3
Chr4
Chr5
Chr6
Chr7
Chr8
Chr9
ChrSy
ChrUn
0e+00
1e+07
2e+07
3e+07
4e+07
Positions on chromosomes
(c)
Reads distribution across genomic regions
Percentage
0.0
0.1
0.2
0.3
0.4
0.5
TSS_10kb
TSS_5kb
TSS_1kb
X5.UTR
CDS
X3.UTR
TES_1kb
TES_5kb
TES_10kb
Intergenic
Intron
PB1_19090041_IP
PB1_19090041_input
Samples

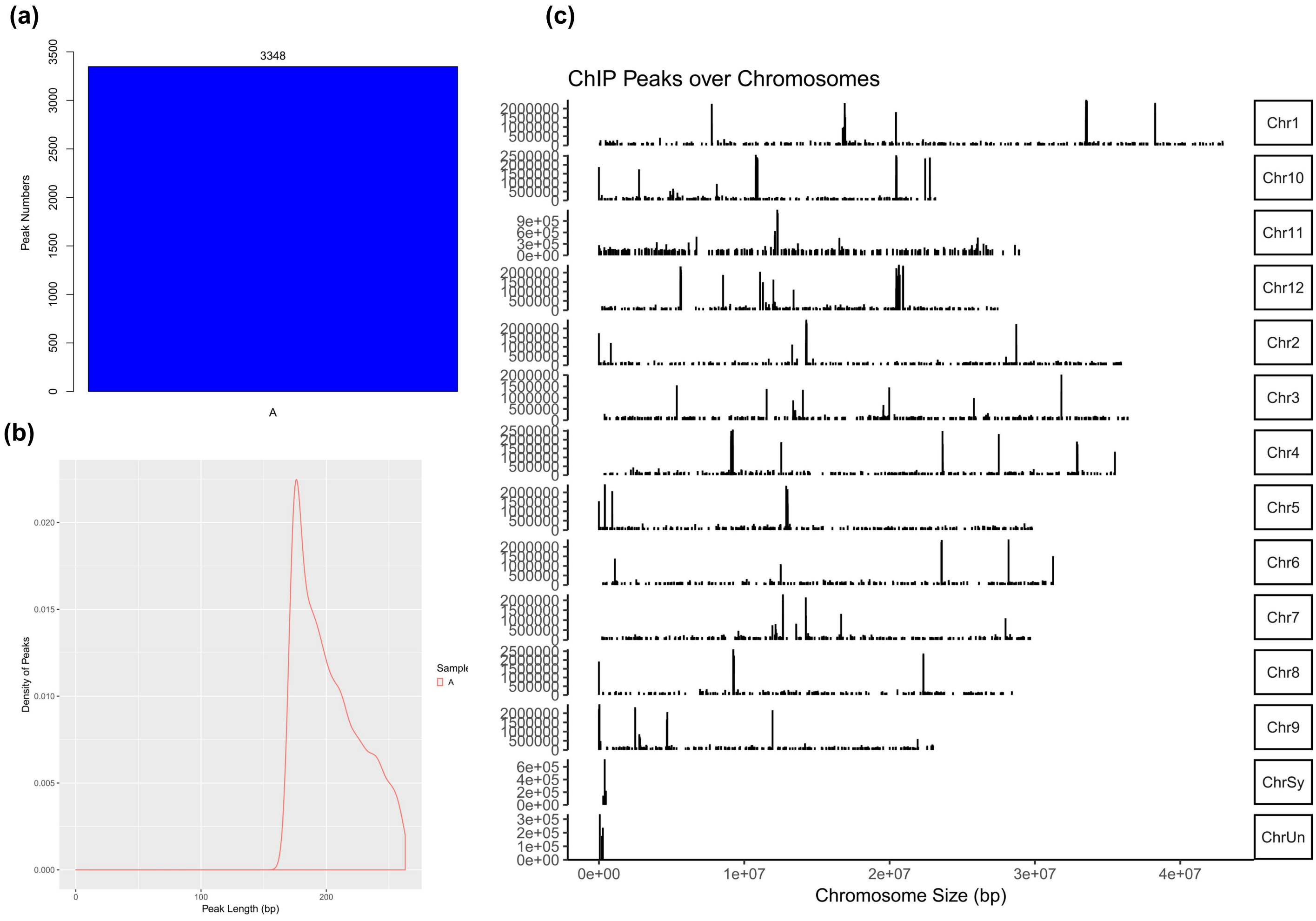
(a)
3348
Peak Numbers
0
500
1000
1500
2000
2500
3000
3500
A
(b)
Density of Peaks
0.000
0.005
0.010
0.015
0.020
0
100
200
Peak Length (bp)
Sample
A
(c)
ChIP Peaks over Chromosomes
Chr1
Chr10
Chr11
Chr12
Chr2
Chr3
Chr4
Chr5
Chr6
Chr7
Chr8
Chr9
ChrSy
ChrUn
0e+00
1e+07
2e+07
3e+07
4e+07
Chromosome Size (bp)

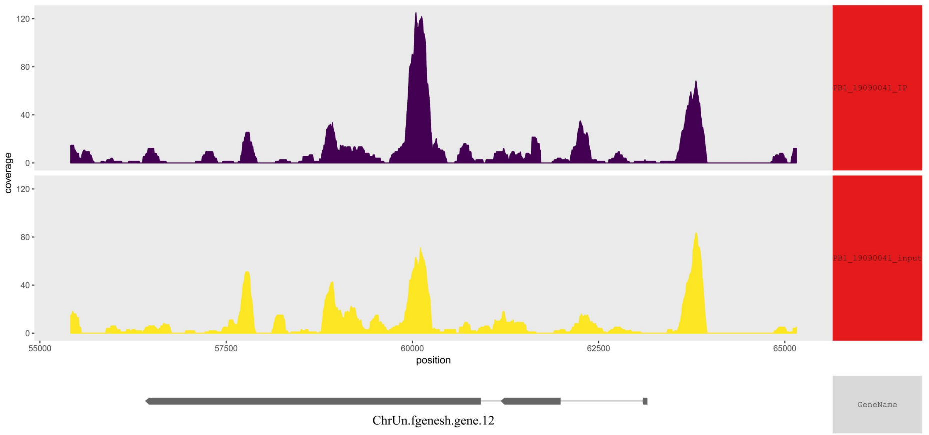
120
80
40
0
coverage
PB1_19090041_IP
PB1_19090041_input
55000
57500
60000
62500
65000
position
GeneName
ChrUn.fgenesh.gene.12

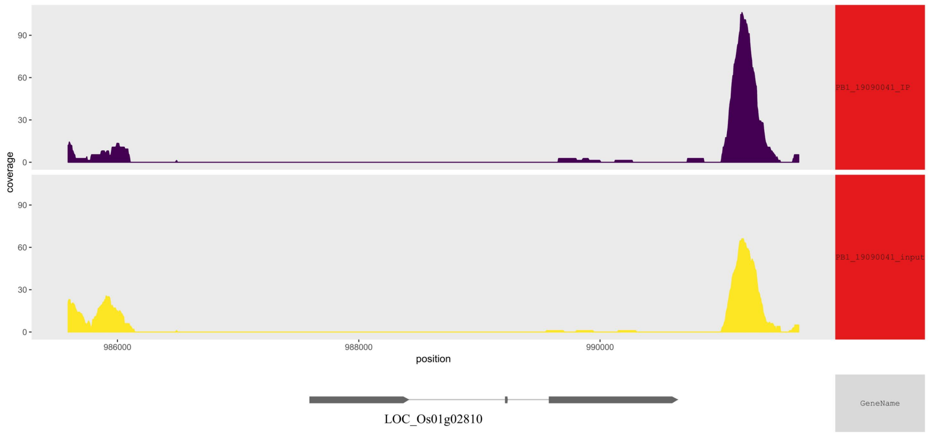
90
60
30
0
coverage
PB1_19090041_IP
PB1_19090041_input
986000
988000
990000
position
GeneName
LOC_Os01g02810

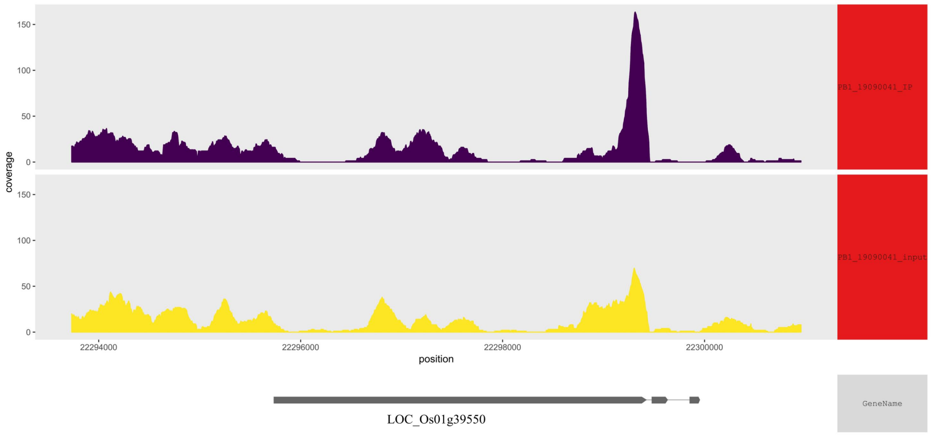
150
100
50
0
coverage
PB1_19090041_IP
PB1_19090041_input
22294000
22296000
22298000
22300000
position
GeneName
LOC_Os01g39550

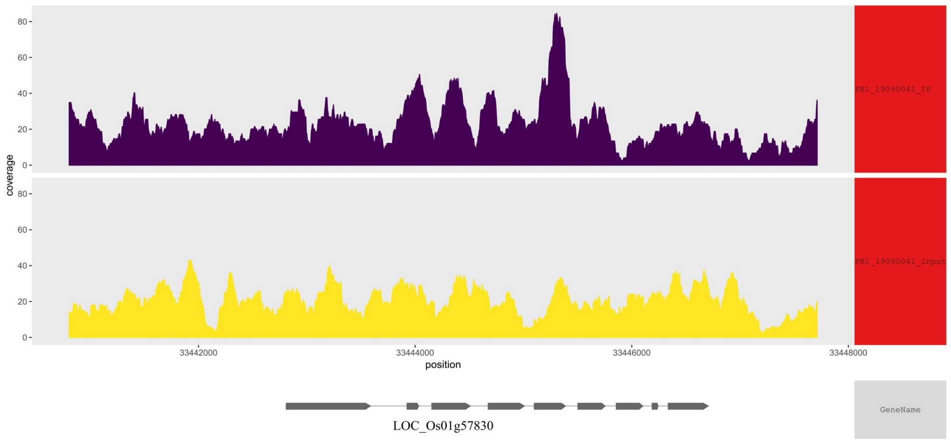
80
60
40
20
0
coverage
PB1_19090041_IP
PB1_19090041_input
33442000
33444000
33446000
33448000
position
GeneName
LOC_Os01g57830

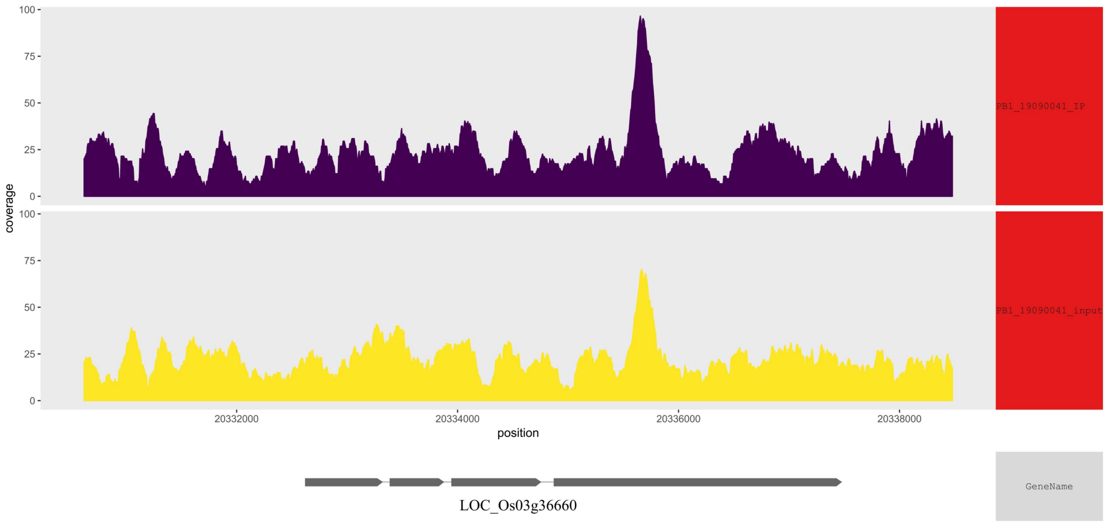
100
75
50
25
0
coverage
PB1_19090041_IP
PB1_19090041_input
20332000
20334000
20336000
20338000
position
GeneName
LOC_Os03g36660

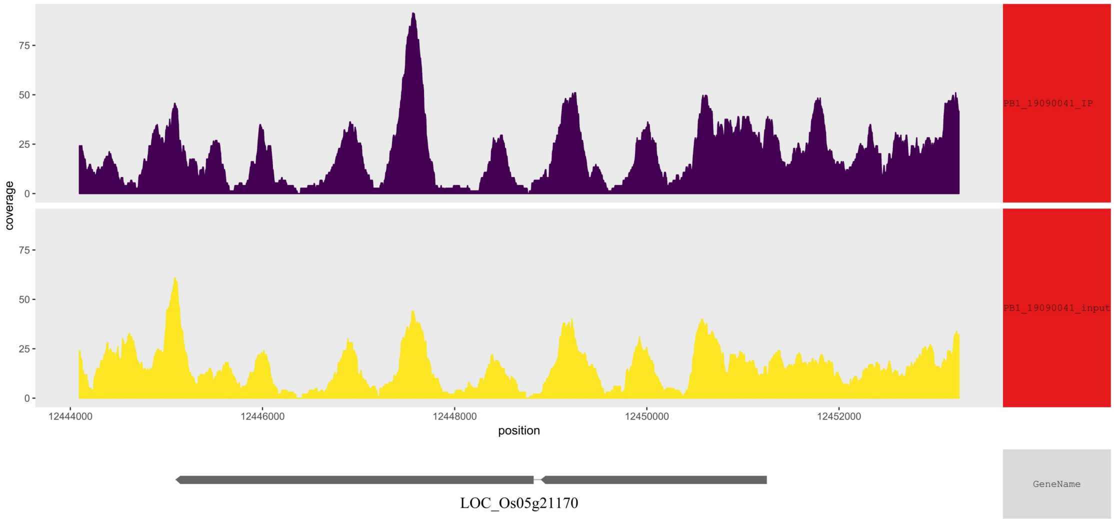
75
50
25
0
coverage
PB1_19090041_IP
PB1_19090041_input
12444000
12446000
12448000
12450000
12452000
position
GeneName
LOC_Os05g21170

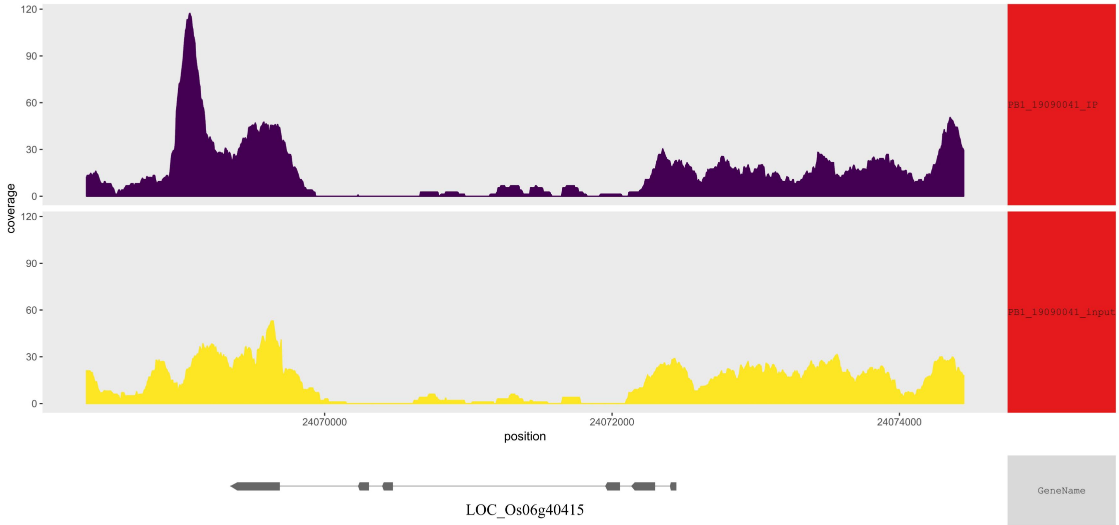
120
90
60
30
0
coverage
PB1_19090041_IP
PB1_19090041_input
24070000
24072000
24074000
position
GeneName
LOC_Os06g40415

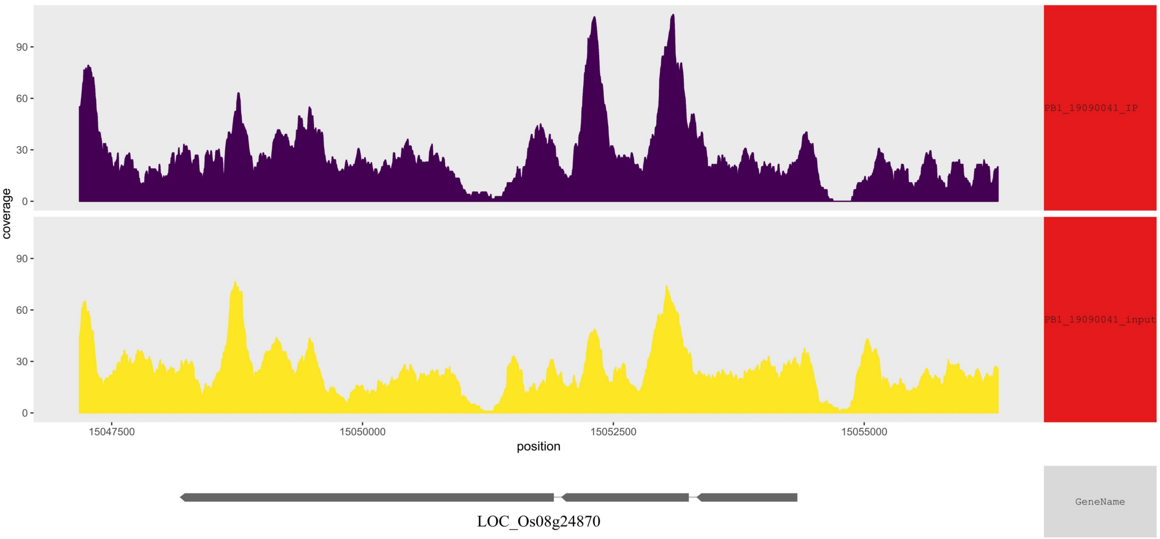
coverage
90
60
30
0
PB1_19090041_IP
PB1_19090041_input
15047500
15050000
15052500
15055000
position
GeneName
LOC_Os08g24870

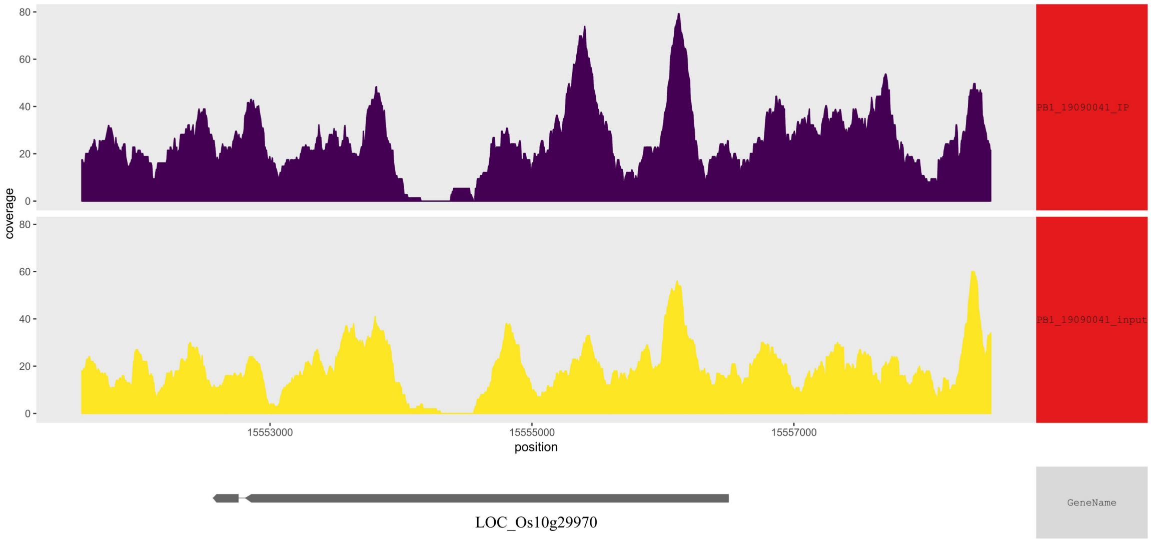
coverage
80
60
40
20
0
PB1_19090041_IP
PB1_19090041_input
15553000
15555000
15557000
position
GeneName
LOC_Os10g29970

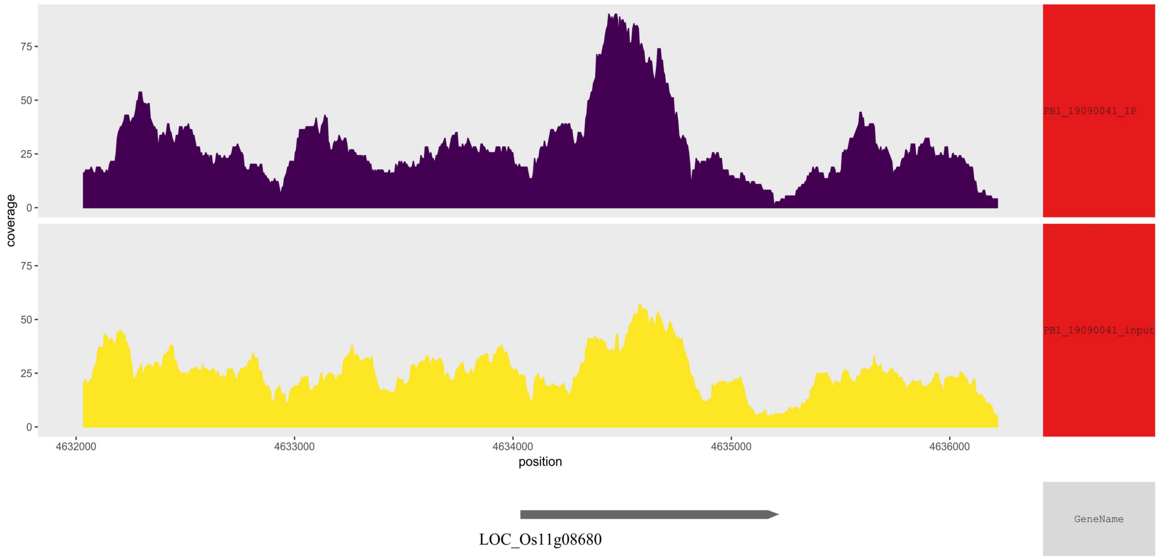
coverage
75
50
25
0
PB1_19090041_IP
PB1_19090041_input
4632000
4633000
4634000
4635000
4636000
position
GeneName
LOC_Os11g08680

Homer Known Motif Enrichment Results (/home/Project/KC2021-F0833/supp/motif/A)

Homer *de novo* Motif Results
Gene Ontology Enrichment Results
Known Motif Enrichment Results (txt file)
Total Target Sequences = 3333, Total Background Sequences = 69000

Rank	Motif	Name	P-value	log P-pvalue	q-value (Benjamini)	# Target Sequences with Motif	% of Targets Sequences with Motif	# Background Sequences with Motif	% of Background Sequences with Motif	Motif File	SVG
1	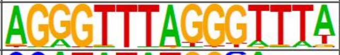	AT4G12670(MYBrelated)/col-AT4G12670-DAP-Seq(GSE60143)/Homer	1e-5	-1.199e+01	0.0057	6.0	0.18%	6.9	0.01%	motif file (matrix)	svg
2	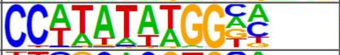	CArG(MADS)/PUER-Srf-ChIP-Seq(Sullivan_et_al.)/Homer	1e-3	-7.954e+00	0.1619	121.0	3.61%	1218.8	2.62%	motif file (matrix)	svg
3	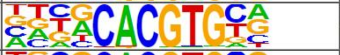	At4g18890(BZR)/col-At4g18890-DAP-Seq(GSE60143)/Homer	1e-3	-7.747e+00	0.1619	87.0	2.60%	827.8	1.78%	motif file (matrix)	svg
4	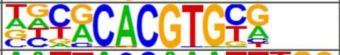	BZR1(BZR)/col-BZR1-DAP-Seq(GSE60143)/Homer	1e-2	-6.877e+00	0.2376	45.0	1.34%	378.4	0.81%	motif file (matrix)	svg
5	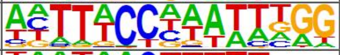	SVP(MADS)/col-SVP-DAP-Seq(GSE60143)/Homer	1e-2	-6.818e+00	0.2376	178.0	5.32%	1956.6	4.20%	motif file (matrix)	svg
6	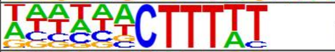	At5g62940(C2C2dof)/col-At5g62940-DAP-Seq(GSE60143)/Homer	1e-2	-6.785e+00	0.2376	1120.0	33.45%	14421.6	30.98%	motif file (matrix)	svg
7	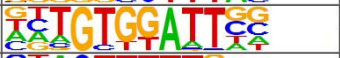	Foxh1(Forkhead)/hESC-FOXH1-ChIP-Seq(GSE29422)/Homer	1e-2	-6.546e+00	0.2376	239.0	7.14%	2736.5	5.88%	motif file (matrix)	svg
8	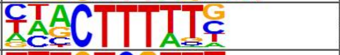	AT1G47655(C2C2dof)/colamp-AT1G47655-DAP-Seq(GSE60143)/Homer	1e-2	-6.524e+00	0.2376	1200.0	35.84%	15542.8	33.39%	motif file (matrix)	svg
9	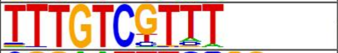	AtIDD11(C2H2)/colamp-AtIDD11-DAP-Seq(GSE60143)/Homer	1e-2	-6.415e+00	0.2376	119.0	3.55%	1249.9	2.69%	motif file (matrix)	svg
10	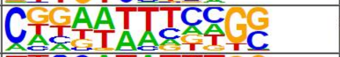	LBD18(LOBAS2)/colamp-LBD18-DAP-Seq(GSE60143)/Homer	1e-2	-6.403e+00	0.2376	960.0	28.67%	12290.2	26.40%	motif file (matrix)	svg
11	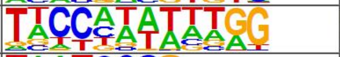	SOC1(MADS)/Seedling-SOC1-ChIP-Seq(GSE45846)/Homer	1e-2	-6.247e+00	0.2376	136.0	4.06%	1463.3	3.14%	motif file (matrix)	svg
12	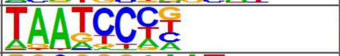	Pitx1(Homeobox)/Chicken-Pitx1-ChIP-Seq(GSE38910)/Homer	1e-2	-6.201e+00	0.2376	1355.0	40.47%	17705.4	38.04%	motif file (matrix)	svg
13	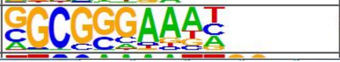	E2F4(E2F)/K562-E2F4-ChIP-Seq(GSE31477)/Homer	1e-2	-6.162e+00	0.2376	322.0	9.62%	3824.3	8.22%	motif file (matrix)	svg

Homer *de novo* Motif Results (/home/Project/KC2021-F0833/supp/motif/A/)

Known Motif Enrichment Results
Gene Ontology Enrichment Results
If Homer is having trouble matching a motif to a known motif, try copy/pasting the matrix file into STAMP
More information on motif finding results: HOMER | Description of Results | Tips
Total target sequences = 3349
Total background sequences = 46571
* - possible false positive

Rank	Motif	P-value	log P-pvalue	% of Targets	% of Background	STD(Bg STD)	Best Match/Details	Motif File
1	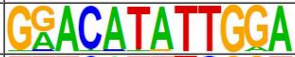	1e-19	-4.513e+01	1.40%	0.25%	56.8bp (57.8bp)	SKO1/MA0382.1/Jaspar(0.669) More Information | Similar Motifs Found	motif file (matrix)
2	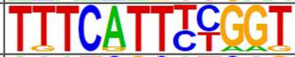	1e-18	-4.283e+01	1.05%	0.14%	50.2bp (52.2bp)	RBM46(RRM)/Homo_sapiens-RNCMPT00054-PBM/HughesRNA(0.670) More Information | Similar Motifs Found	motif file (matrix)
3	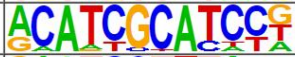	1e-18	-4.248e+01	1.28%	0.22%	51.1bp (49.4bp)	CHA4(MacIsaac)/Yeast(0.767) More Information | Similar Motifs Found	motif file (matrix)
4	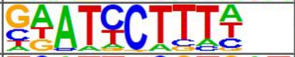	1e-18	-4.230e+01	8.66%	4.99%	54.7bp (58.2bp)	CG5213(RRM)/Drosophila_melanogaster-RNCMPT00010-PBM/HughesRNA(0.743) More Information | Similar Motifs Found	motif file (matrix)
5	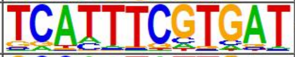	1e-17	-4.108e+01	1.40%	0.28%	55.9bp (60.7bp)	AT2G31460(REMB3)/col-AT2G31460-DAP-Seq(GSE60143)/Homer(0.735) More Information | Similar Motifs Found	motif file (matrix)
6	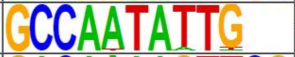	1e-16	-3.862e+01	1.28%	0.25%	54.6bp (56.6bp)	Arid5a/MA0602.1/Jaspar(0.810) More Information | Similar Motifs Found	motif file (matrix)
7	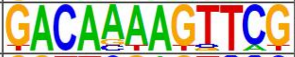	1e-16	-3.792e+01	1.31%	0.27%	53.1bp (62.8bp)	Tb_0217(RRM)/Trypanosoma_brucei-RNCMPT00217-PBM/HughesRNA(0.699) More Information | Similar Motifs Found	motif file (matrix)
8	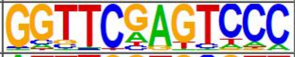	1e-16	-3.726e+01	0.54%	0.03%	44.7bp (45.1bp)	OPI1/Literature(Harbison)/Yeast(0.730) More Information | Similar Motifs Found	motif file (matrix)
9	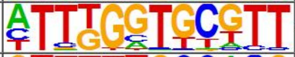	1e-15	-3.631e+01	1.55%	0.39%	59.1bp (59.6bp)	ceh-10::ttx-3/MA0263.1/Jaspar(0.719) More Information | Similar Motifs Found	motif file (matrix)
10	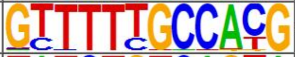	1e-15	-3.459e+01	1.55%	0.40%	57.2bp (63.0bp)	RPN4(MacIsaac)/Yeast(0.739) More Information | Similar Motifs Found	motif file (matrix)
11	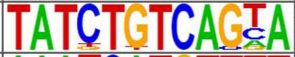	1e-15	-3.458e+01	0.33%	0.01%	46.6bp (36.3bp)	CUP9/MA0288.1/Jaspar(0.735) More Information | Similar Motifs Found	motif file (matrix)
12	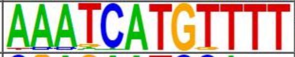	1e-14	-3.323e+01	1.16%	0.24%	55.7bp (49.6bp)	Pp_0229(RRM)/Physcomitrella_patens-RNCMPT00229-PBM/HughesRNA(0.710) More Information | Similar Motifs Found	motif file (matrix)
13	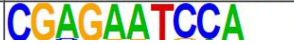	1e-14	-3.308e+01	5.47%	2.93%	53.9bp (58.2bp)	PB0139.1_Irf5_2/Jaspar(0.744) More Information | Similar Motifs Found	motif file (matrix)

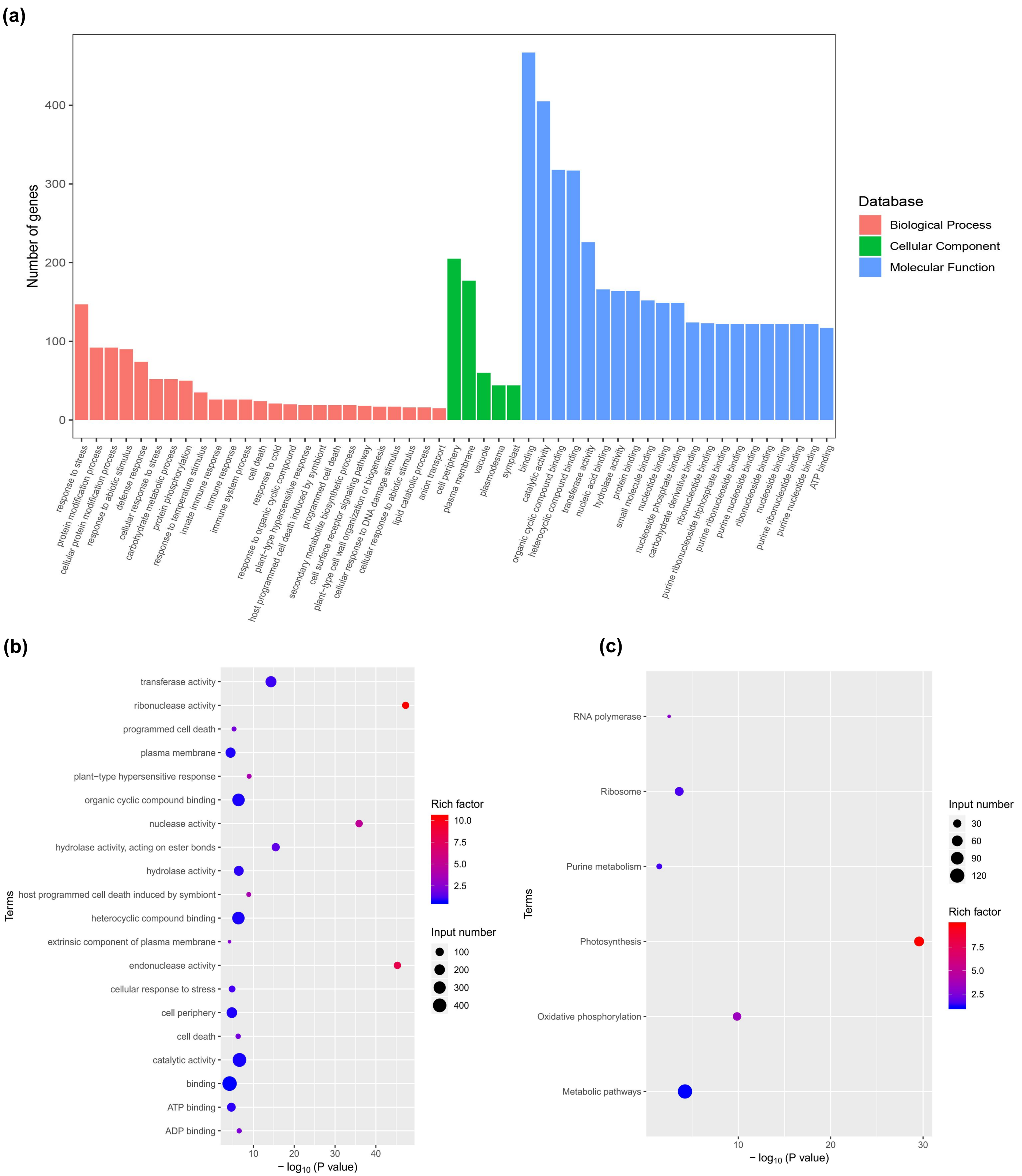

(a)
Number of genes
0
100
200
300
400
Database
Biological Process
Cellular Component
Molecular Function
response to stress
protein modification process
cellular protein modification process
response to abiotic stimulus
defense response
cellular response to stress
carbohydrate metabolic process
protein phosphorylation
response to temperature stimulus
innate immune response
immune response
immune system process
cell death
response to cold
response to organic cyclic compound
plant−type hypersensitive response
host programmed cell death induced by symbiont
programmed cell death
secondary metabolite biosynthetic process
cell surface receptor signaling pathway
plant−type cell wall organization or biogenesis
cellular response to DNA damage stimulus
cellular response to abiotic stimulus
lipid catabolic process
anion transport
cell periphery
plasma membrane
vacuole
plasmodesma
symplast
binding
catalytic activity
organic cyclic compound binding
heterocyclic compound binding
transferase activity
nucleic acid binding
hydrolase activity
protein binding
small molecule binding
nucleotide binding
nucleoside phosphate binding
carbohydrate derivative binding
ribonucleotide binding
purine ribonucleoside triphosphate binding
purine ribonucleoside binding
purine nucleoside binding
ribonucleoside binding
nucleoside binding
purine ribonucleotide binding
purine nucleotide binding
ATP binding
(b)
Terms
transferase activity
ribonuclease activity
programmed cell death
plasma membrane
plant−type hypersensitive response
organic cyclic compound binding
nuclease activity
hydrolase activity, acting on ester bonds
hydrolase activity
host programmed cell death induced by symbiont
heterocyclic compound binding
extrinsic component of plasma membrane
endonuclease activity
cellular response to stress
cell periphery
cell death
catalytic activity
binding
ATP binding
ADP binding
10
20
30
40
− log10 (P value)
Rich factor
10.0
7.5
5.0
2.5
Input number
100
200
300
400
(c)
Terms
RNA polymerase
Ribosome
Purine metabolism
Photosynthesis
Oxidative phosphorylation
Metabolic pathways
10
20
30
− log10 (P value)
Input number
30
60
90
120
Rich factor
7.5
5.0
2.5

Rice SNP-Seek Database

Home Search Browse My Lists Order Seeds Download Help

By using SNP-Seek, you abide by the data use license stated here , and development here

OsMYB73-Haplotype analysis

(a)

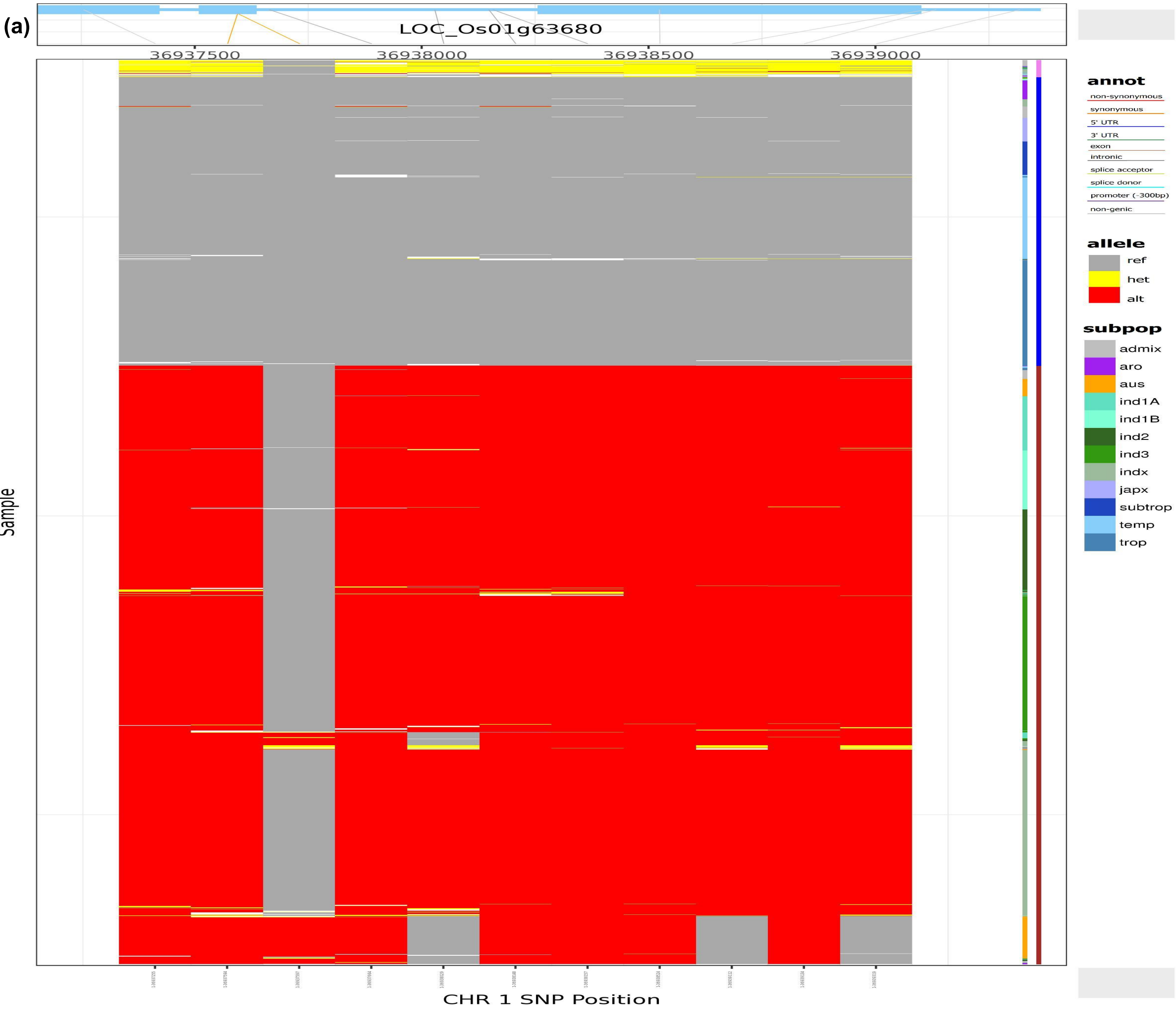

(b)

KGROUP	SUBPOPS:COUNT	VARIETIES	FREQUENCY	chr01	chr01	chr01	chr01	chr01	chr01	chr01	chr01	chr01	chr01	chr01
1	indx:580/ind1B:199/au	2002	66.20	G	A	T	G	A	G	G	T	T	T	C
2	ind3:4/admix:23/indx:1	56	1.85	A	G	T	T	C	G	C	C	G	C	G
3	admix:38/trop:359/tem	966	31.94	A	G	T	T	C	T	C	C	G	C	G

(c)

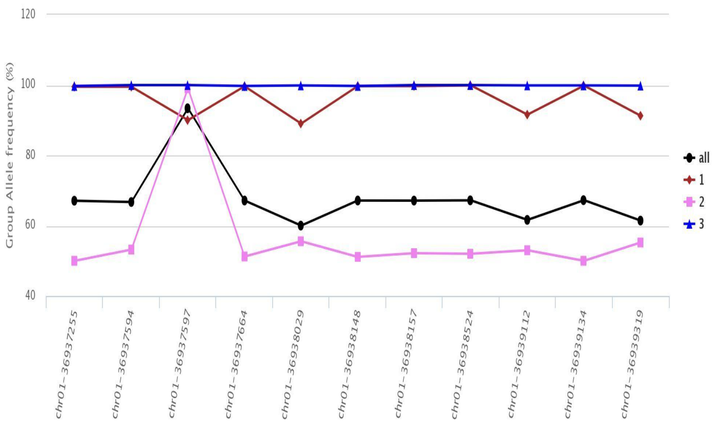

RFGB for Rice Functional Genomics & Breeding

Version 3.0

Home | Search | Analyze | Tool | Datacenter | About

Home > Analyze > Haplotype

haplotype

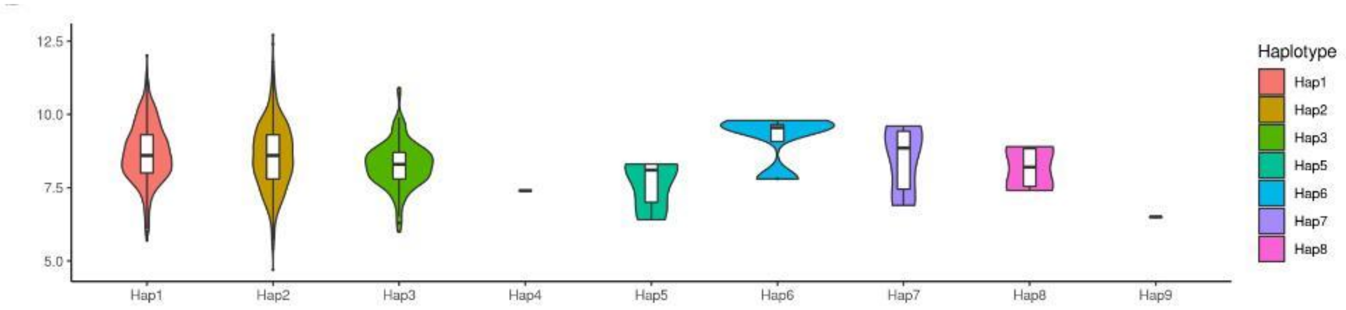

Download - GL

GLWR

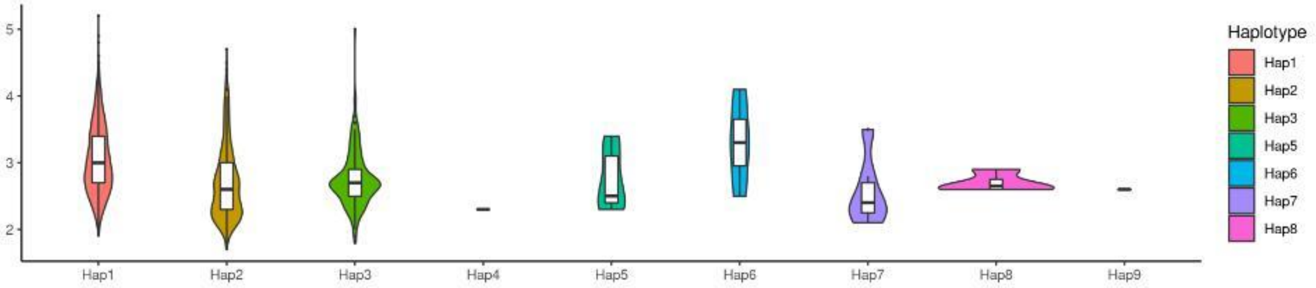

Download - GLWR

TGW

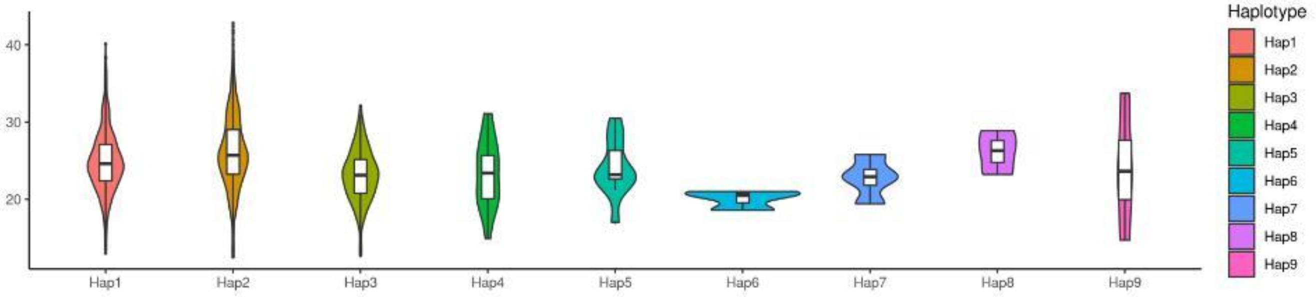

Download - TGW

Haplotypes	SNPs	Sample List	Group	Grain Length (2013)	Grain Length/Width Ratio (2013)	Thousand Grain Weight (1847)
Hap1	CCCGAAGTGGCCTGCCGG	1346	Aus: 1<br>Bas: 1<br>GJ: 12<br>XI: 1317<br>admix: 14<br>na: 1	(914 of 1346)<br>mean: 8.742	(914 of 1346)<br>mean: 3.081	(789 of 1346)<br>mean: 25.012
Hap2	ACAGGTACTGTTTTTTAT	786	Aus: 1<br>Bas: 59<br>GJ: 665<br>XI: 33<br>admix: 28	(525 of 786)<br>mean: 8.559	(525 of 786)<br>mean: 2.696	(519 of 786)<br>mean: 26.001
Hap3	CCCGAAGTGATCTGCCGG	317	Aus: 55<br>GJ: 1<br>XI: 249<br>admix: 12	(252 of 317)<br>mean: 8.389	(252 of 317)<br>mean: 2.895	(177 of 317)<br>mean: 24.287
Hap4	CGCGAAGTGGTCTGCCGG	193	Aus: 132<br>Bas: 3<br>GJ: 1<br>XI: 50<br>admix: 7	(146 of 193)<br>mean: 8.269	(146 of 193)<br>mean: 2.761	(136 of 193)<br>mean: 23.037
Hap5	ACAGGTACTGTTCTTTAT	72	GJ: 68<br>admix: 4	(61 of 72)<br>mean: 8.905	(61 of 72)<br>mean: 2.798	(40 of 72)<br>mean: 27.173
Hap6	ACAAGTACTGTTTTTTAT	35	GJ: 34<br>admix: 1	(18 of 35)<br>mean: 7.961	(18 of 35)<br>mean: 2.417	(25 of 35)<br>mean: 28.472
Hap7	CCCGAAGTGRYCTGCCGG	17	XI: 17	(1 of 17)<br>mean: 8.300	(1 of 17)<br>mean: 2.700	(11 of 17)<br>mean: 23.327
